# Stochastic pausing at latent HIV-1 promoters generates transcriptional bursting

**DOI:** 10.1038/s41467-021-24462-5

**Published:** 2021-07-23

**Authors:** Katjana Tantale, Encar Garcia-Oliver, Marie-Cécile Robert, Adèle L’Hostis, Yueyuxiao Yang, Nikolay Tsanov, Rachel Topno, Thierry Gostan, Alja Kozulic-Pirher, Meenakshi Basu-Shrivastava, Kamalika Mukherjee, Vera Slaninova, Jean-Christophe Andrau, Florian Mueller, Eugenia Basyuk, Ovidiu Radulescu, Edouard Bertrand

**Affiliations:** 1 Institut de Génétique Moléculaire de Montpellier, University of Montpellier, CNRS, Montpellier, France; 2 Equipe labélisée Ligue Nationale Contre le Cancer, University of Montpellier, CNRS, Montpellier, France; 3 Institut de Génétique Humaine, University of Montpellier, CNRS, Montpellier, France; 4 LPHI, UMR CNRS 5235, University of Montpellier, Montpellier, France; 5 Unité Imagerie et Modélisation, Institut Pasteur and CNRS UMR 3691, Paris, France; 6 Present Address: Microbiology Fundamental and Pathogenicity CNRS UMR 5234, University of Bordeaux, Bordeaux, France

**Keywords:** Gene expression, Transcription, Cellular noise, Single-cell imaging

## Abstract

Promoter-proximal pausing of RNA polymerase II is a key process regulating gene expression. In latent HIV-1 cells, it prevents viral transcription and is essential for latency maintenance, while in acutely infected cells the viral factor Tat releases paused polymerase to induce viral expression. Pausing is fundamental for HIV-1, but how it contributes to bursting and stochastic viral reactivation is unclear. Here, we performed single molecule imaging of HIV-1 transcription. We developed a quantitative analysis method that manages multiple time scales from seconds to days and that rapidly fits many models of promoter dynamics. We found that RNA polymerases enter a long-lived pause at latent HIV-1 promoters (>20 minutes), thereby effectively limiting viral transcription. Surprisingly and in contrast to current models, pausing appears stochastic and not obligatory, with only a small fraction of the polymerases undergoing long-lived pausing in absence of Tat. One consequence of stochastic pausing is that HIV-1 transcription occurs in bursts in latent cells, thereby facilitating latency exit and providing a rationale for the stochasticity of viral rebounds.

## Abstract (web summary)

The ability of HIV to alternate between acute and latent forms is thought to rely on a transcriptional feedback loop where polymerase pausing is released by the viral protein Tat. Here, the authors show that viral genome transcription can occur in a burst-like stochastic manner in the absence of Tat.

## Introduction

Transcription initiation is a complex process that comprises chromatin opening, assembly of a pre-initiation complex (PIC), polymerase recruitment and its maturation into an elongation-competent form (see ref. ^1^ for review). In *Drosophila* and mammals, this last step is highly regulated and appears to be a key point in the control of gene expression (ref. ^2^ for review). RNA polymerase II (RNAPII) is recruited by the PIC in a hypo-phosphorylated form and is then loaded on a short stretch of single-stranded DNA, which is melted by TFIIH. The initiating polymerase starts elongating about a dozen of nucleotides and must undergo a number of modifications before leaving the promoter and entering productive elongation^3^. First, the TFIIH-associated CDK7 kinase phosphorylates the Serine 5 of the heptad repeats of the C-terminal domain (CTD) of RNAPII, thereby disrupting the interaction with Mediator and facilitating promoter escape (refs. ^4,5^ for reviews). The S5 phosphorylated CTD also recruits the RNA capping enzymes that access the RNA 5′-end when it emerges from the polymerase^6^. The polymerase then transcribes an additional 10–80 nucleotides and typically enters a paused state. Two factors appear particularly important to trigger pausing, in relation to TFIID^7^: DSIF (DRB sensitivity-inducing factor), which is composed of SPT4 and SPT5, and NELF (negative elongation factor), a four subunit complex that also interacts with the cap-binding complex (CBC^8^). A recent structure of the pausing complex indicates that the RNA-DNA hybrid adopts a tilted conformation within the polymerase that prevents further nucleotide addition^9^. This structure is stabilized by NELF and DSIF, which also prevent binding of TFIIS, a factor that can trigger cleavage of the RNA at the active site to restart backtracked polymerases^10^. Release from the paused state requires the positive transcription elongation factor b (P-TEFb), which is composed of Cyclin T1 or T2 associated with the kinase CDK9^11^, sometimes in association with the super-elongation complex (SEC^12,13^). P-TEFb is activated by CDK7^4,5,14^ and it phosphorylates a number of components of the pausing complex to enable the formation of an elongation-competent polymerase^9,15,16^. Phosphorylation of NELF triggers its dissociation from the polymerase, and this frees a binding site for PAF, an elongation factor that is required for transcription through chromatin. P-TEFb also phosphorylates the RNA polymerase CTD on its Serine 2, as well as the linker between the polymerase core and the CTD, creating a binding site for the elongation factor SPT6^9^. DSIF functions both as a repressor and activator of elongation, and it is also phosphorylated by P-TEFb (^17^ and ref therein). The structures of the paused and active elongation complex show that DSIF adopts different conformations in the two complexes. In particular, phosphorylated DSIF frees the nascent RNA and allows the polymerase to clamp around the DNA, promoting elongation while preventing the release of the polymerase from DNA. Overall, P-TEFb mediated phosphorylation thus disrupts the pausing complex and triggers formation of an active elongation complex comprising the polymerase associated with DSIF, SPT6, and PAF.

While pausing is thought to be a key regulatory point for many cellular promoters in mammals and *Drosophila*, it is often revealed by a peak of RNAPII near the promoter that can in fact correspond to different molecular processes such as slow elongation, polymerase arrest, or defective processivity (i.e. abortive initiation^18^). Recent efforts have been made to clarify these mechanisms by measuring pausing duration. These studies indicated that pausing time varies from less than a minute up to an hour in *Drosophila* and mammals, depending on the promoter^19–23^. This revealed a surprising variability in pausing kinetics, with widely different regulatory potential.

Another major finding of the last 15 years is that transcription is a discontinuous process in vivo (^24^ see^25,26^ for reviews), with “active” genes going through active and inactive periods in a stochastic manner, a phenomenon also called transcriptional noise or gene bursting. In particular, recent evidences suggest that for many genes, expression levels are dynamically encoded in the time domain by controlling the periods during which a gene is active, rather than by regulating the initiation rate^27–29^. Major efforts have been made to decipher the causes of gene bursting and in particular the molecular status of the postulated ON and OFF states. Indeed, the transitions between these states are kinetically rate limiting and therefore represent key regulatory checkpoints. However, despite these efforts and the importance of pausing in regulating gene expression, how pausing affects gene bursting remains not characterized.

An important implication of gene bursting is that it creates cell-to-cell heterogeneity and this has multiple consequences on the phenotypes of single cells or multicellular organisms. For instance, stochasticity in the expression of Heat-Shock genes in yeasts is thought to help a fraction of the yeast population to survive sublethal stresses^30^, while in C. Elegans, mutations in a small gene regulatory network create a high expression variability, ultimately leading to variable phenotypic penetrance of the mutation^31^. In the case of HIV-1, transcriptional noise is thought to play a crucial role in the control of latency. Indeed, HIV-1 infection generates latent cells that can persist in the body for decades and can re-establish viral propagation when antiviral treatments are interrupted. Previous studies from the Siliciano and Weinberger labs have shown that latency exit is stochastic and possibly linked to random fluctuations of viral transcription^32–34^. How the viral promoter creates bursts of gene expression in latent cells is not understood, but nevertheless fundamental as it is triggering latency exit. A better knowledge of mechanistic and quantitative aspects of the reactivation dynamics is indeed essential for the development of new strategies in combinatorial anti-retroviral therapies such as “shock and kill” and “block and lock”.

**Fig. 1:**
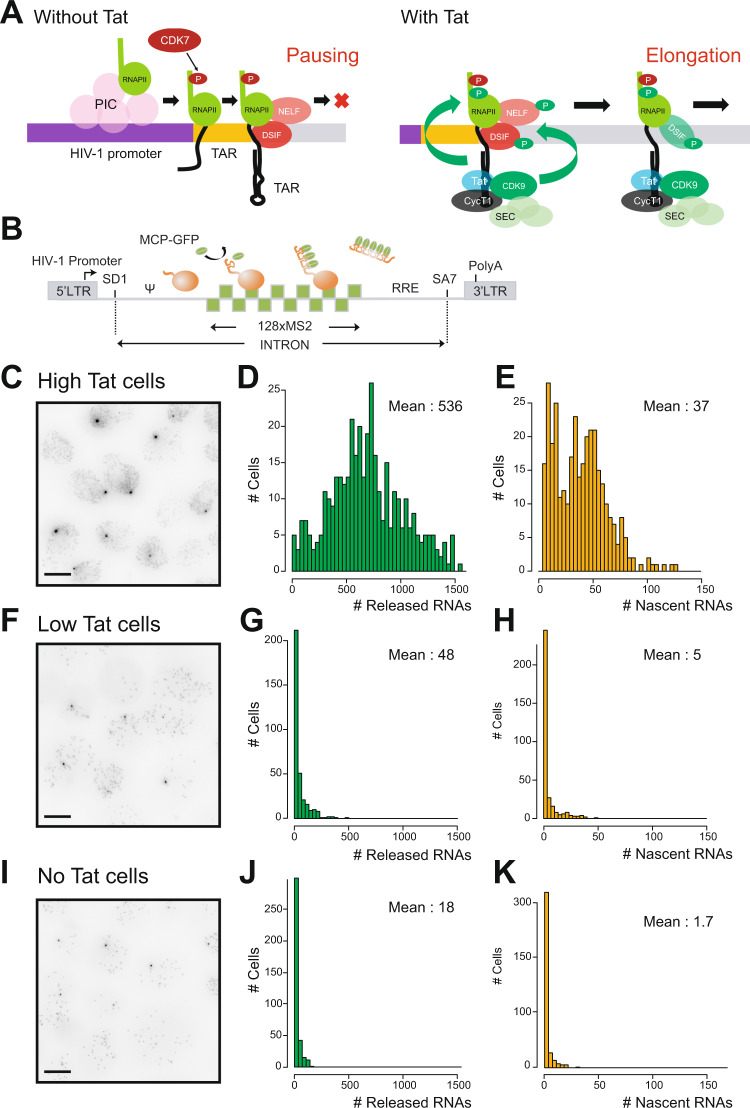
Single cell characterization of HIV-1 gene expression, with and without Tat. **A** Schematic of HIV-1 transcriptional regulation. Left: in the absence of Tat, pTEFb is not recruited and polymerases binds NELF and DSIF and pause near the promoter. Right: in the presence of Tat, pTEFb, composed of Cyclin T1 and Cdk9 associated with the super-elongation complex, is recruited to the nascent TAR RNA. Cdk9 phosphorylates NELF, DSIF, and RNA polymerase II, thereby triggering pausing exit and processive elongation. **B** Schematic of the HIV-1 reporter construct. SD1: major HIV-1 splice site donor; SA7: last HIV-1 splice site acceptor; ψ: packaging signal; RRE: Rev-responsive element; LTR: long terminal repeat. **C**–**E**. Expression of the 128xMS2 HIV-1 tagged reporter in cells expressing high levels of Tat. C- microscopy images of High Tat HeLa cells where the unspliced HIV-1 pre-mRNA is detected by smFISH with probes against the 128xMS2 tag. Cells bear a single copy of the reporter gene integrated with the Flp-in system. The bright spots in the nuclei correspond to nascent RNA at their transcription sites, while the dimmer spots correspond to single pre-mRNA molecules. Scale bar: 10 μm. This experiment has been done three times with similar results. **D** distribution of the number of released HIV-1 pre-mRNAs per cell, in High Tat cells. Experimental RNA distributions are from smFISH data. X-axis: number of HIV-1 pre-mRNA molecules per cell; y-axis: number of cells; inset: mean number of HIV-1 pre-mRNAs per cell. **E** distribution of the number of nascent HIV-1 pre-mRNAs per transcription site, in High Tat cells. Experimental RNA distribution is from smFISH data. *X*-axis: number of nascent HIV-1 pre-mRNA molecules per transcription site; *y*-axis: number of transcription sites; inset: mean number of nascent HIV-1 pre-mRNAs per cell. **D**, **E** source data are provided as a Source Data file. **F**–**H** Expression of the 128xMS2 HIV-1 tagged reporter in cells expressing low levels of Tat. Legend as in (**C**–**E**), except that experiments are from Low Tat cells. This experiment has been done three times with similar results. **G**, **H** source data are provided as a Source Data file. **I**–**K** Expression of the 128xMS2 HIV-1 tagged reporter in cells not expressing Tat. Legend as in (**C**–**E**), except that experiments are from No Tat cells. Image contrast adjustment is identical for panels **C**, **F**, and **I**. This experiment has been done four times with similar results. **J**, **K** Source data are provided as a Source Data file.

The ability of the virus to alternate between acute and latent forms lies in a positive transcriptional feedback loop established by the viral protein Tat (^32^, see^35,36^ for reviews). In latent cells, Tat levels are very low and viral transcription remains low or silent. In acutely infected cells, Tat levels are elevated, strongly inducing viral transcription. It is well established that in the absence of Tat or when Tat levels are low, P-TEFb is limiting for viral transcription and the polymerases that initiate transcription enter a paused state after transcribing about 60 nucleotides, and fail to enter productive elongation (reviewed in^35,36^; Fig. 1A, left). Tat alleviates this block by binding both P-TEFb and the TAR stem-loop at the 5′-end of nascent HIV-1 RNAs, leading to the formation of a ternary complex that promotes elongation by recruiting P-TEFb and its associated super-elongation complex to paused polymerases (^11–13^; Fig. 1A, right). The HIV-1 promoter is thus strictly regulated at the level of pausing and P-TEFb recruitment, and these steps are controlled by Tat, which overall can activate viral transcription by more than 100 fold. These properties make HIV-1 an attractive model to decipher how pausing affects gene bursting, with direct relevance for HIV-1 latency and pathogenesis^37,38^.

Here, we imaged HIV-1 transcription in live cells at the level of single polymerases. We characterized the effect of pausing on gene bursting by modulating the levels of Tat, which controls pausing at the HIV-1 promoter. We provide the first fully quantitative description of the stochastic activity of the HIV-1 promoter in basal and induced conditions, on timescales ranging from second to tens of hours. Surprisingly, we found that promoter-proximal pausing is a stochastic event that generates large viral bursts even in cells that do not express Tat. In HIV-1 latent cells with a functional but inactive Tat loop, stochastic pausing may be a key phenomenon that determines latency exit.

## Results

### Single molecule imaging of HIV-1 transcription with different levels of Tat

We previously developed an improved MS2 tagging system based on a 128xMS2 tag, designed for long-term tracking of single RNAs^28^. To image HIV-1 transcription, we inserted this tag in the intron of an HIV-1 vector that had all the viral sequences responsible for transcription and RNA processing (Fig. 1A, B). The corresponding pre-mRNA splices entirely post-transcriptionally, enabling imaging of transcription independently of splicing^28,39^. The high number of MS2 stem-loops present in this reporter allows for a 5-fold increase in signal as compared to our original 24xMS2 repeat^40^. This enables the use of a low illumination power to limit photobleaching, allowing to capture five times more images while still detecting single RNA molecules. By using the 128xMS2 tag and monitoring the brightness of the transcription site over time, it is possible to measure promoter activity with a temporal resolution in the second range and for hours.

It has been demonstrated by numerous studies that the HIV-1 promoter is regulated at the level of promoter-proximal pausing (see refs. ^35,36^ for reviews). Indeed, latent cells do not express a significant amount of Tat and in this case, polymerases that start transcribing are blocked ~60 nucleotides downstream the transcription start site and do not enter productive elongation. This block is relieved by Tat, which directly alleviates pausing by recruiting P-TEFb to the nascent viral RNAs and allowing polymerases to elongate throughout the entire viral genome. To characterize how pausing affects HIV-1 transcription, we therefore created isogenic cell lines expressing different levels of Tat. These lines all contained the 128xMS2 reporter integrated at the same chromosomal location. We previously generated a HeLa cell line that expressed in *trans* a saturating amount of Tat (*High Tat* cells). In these cells, a further increase in the amount of Tat did not lead to more viral transcription^28^ and pausing was therefore not rate limiting. Indeed, transcription was very strong in *High Tat* cells, and we moreover found that Mediator was important for the rapid successive polymerase firing by a reinitiation mechanism (every 3-4 s), while the TATA box was required to continuously maintain the HIV-1 promoter in an active state^28^. To determine the effect of pausing on bursting dynamics, we thus created two new reporter cell lines with low levels of Tat to mimic the situation of latent cells where Tat is not expressed or only at very low levels^35,36^. The first cell line expresses Tat from the second cistron of a bicistronic vector (referred to as *Low Tat* cells), and Tat was not detected by Western blot although it promoted HIV-1 transcription by 2.7 fold (Figs. 1C–K and Fig. S1A). The second cell line entirely lacked Tat (referred to as *No Tat*). We first determined the expression levels of the HIV-1 reporter by performing smFISH experiments with probes binding the 128xMS2 repeat. We found that expression of the HIV-1 reporter depended on Tat as expected (Fig. 1C–K), as the number of pre-mRNA molecules present in the nucleoplasm dropped from ~500 copies per nucleus in *High Tat* cells, to ~50 and ~20 in *Low Tat* and *No Tat* cells, respectively. This was mirrored by a similar decrease in the level of the nascent RNAs present at the transcription sites, with a mean of 37 copies for the *High Tat* cells, and only 5 and 1.7 for the *Low Tat* and *No Tat* cells, respectively (Fig. 1C–K).

Next, we aimed at confirming that pausing was limiting viral transcription in *No Tat* cells. To this end, we overexpressed the two subunits of P-TEFb, Cdk9 and Cyclin T1, by transient transfection. We observed that this increased viral transcription as previously reported in other cellular systems (Fig. S1B;^41^). Then, we fused CDK9 to a fluorescent catalytically inactive Cas9 variant (dCas9-tagBFP), and we transfected the resulting construct in *No Tat* cells together with vectors expressing three Cas9 guide RNAs targeting the HIV-1 promoter. By performing smFISH with probes against the 128xMS2 repeat, we found that expressing dCas9-CDK9-tagBFP alone increased HIV-1 RNA levels by 4 fold, while targeting it to the HIV-1 promoter with three guide RNAs led to a 10 fold increase in expression (Fig. S1C). Moreover, the basal HIV-1 transcriptional activity in *No Tat* cells was blocked when P-TEFb was inactivated with KM05283, a drug that specifically inhibits CDK9 kinase activity (Fig. S2A). This indicated that in *No Tat* cells, P-TEFb was both required for basal transcription and was also limiting viral expression, providing functional indications that pausing was limiting in the absence of Tat. Next, we tested whether the basal viral transcription observed without Tat was due to sporadic activation of the NF-κB pathway, as it is a well-known activator of the HIV-1 promoter that can recruit P-TEFb^42,43^. We treated cells with BAY11-7082, a drug that inhibits the IKK kinase and traps NF-κB subunits in the cytoplasm. No difference in HIV-1 expression was seen after 16 h of treatment, indicating that the basal viral transcription was independent of NF-κB (Fig. S2B, C). Taken together, these data indicate that in our cellular system, the basal HIV-1 transcription occurring in the absence of Tat is P-TEFb dependent, and that the recruitment of this factor is a key step limiting viral transcription, as expected from a large body of previous studies.

### The absence of Tat does not affect the formation of polymerase convoys but creates long inactive periods

**Fig. 2:**
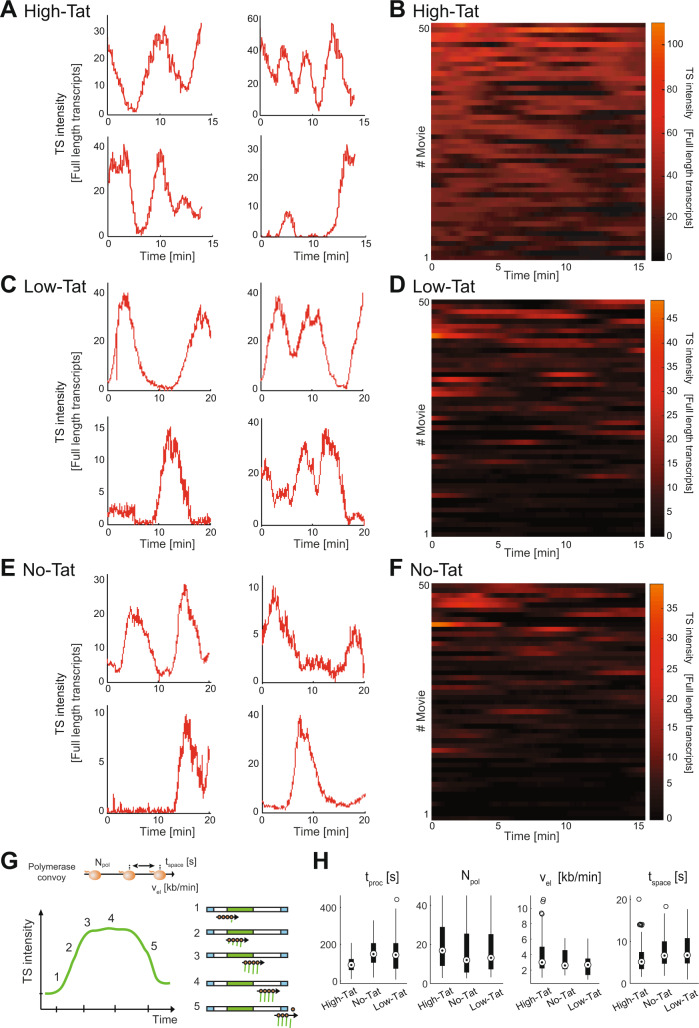
Fluctuation of HIV-1 transcription over short time periods, with and without Tat. **A**–**F** Fluctuations of HIV-1 transcription over 15–20 min periods, with one image stack recorded every 3 s. **A**, **C**, **E** Each graph is a single transcription site; the *x*-axis represents the time (in minutes) and *y*-axis represents the intensity of transcription sites, expressed in equivalent numbers of full-length pre-mRNA molecules. **B**, **D**, **F** Each line is a cell and the transcription site intensity is color-coded (scale on the right). **A**, **B** High-Tat cells. **C**, **D** Low-Tat cells. **E**, **F** No-Tat cells. Source data are provided as a Source Data file. **G** Schematic of a polymerase convoy. Top: a polymerase convoy, with polymerases in orange and the gene represented as a black horizontal arrow. *N*_pol_: number of polymerases; *t*_space_: spacing between successive RNA polymerases (in seconds); *v*_el_: elongation rate. Bottom: schematics describing the different phases of a transcription cycle (left) and the position of the polymerase convoy on the MS2 tagged gene (right; the green box is theMS2 tag). **H** Box-plots representing the parameters values of the best-fit models, measured for a set of isolated transcription cycles in each cell line (*n* = 89, 36, and 59 for *High Tat*, *No Tat,* and *Low Tat*, respectively). *t*_proc_ is the 3′-end RNA processing time; *N*_pol_ is the number of polymerases in the convoy; *V*_el_ is the elongation rate (in kb/min); *t*_space_ is the spacing between successive polymerase (in seconds). The bottom line displays the first quartile, the box corresponds to the second and third quartile, the top line to the last quartile, and the double circle is the median. Small circles are outliers (1.5 times the inter-quartile range above or below the upper and lower quartile, respectively). Source data are provided as a Source Data file.

When Tat is in excess, HIV-1 transcription occurs in the form of polymerase convoys, i. e. sets of closely spaced polymerases that transcribe the gene together (see schematic in Fig. 2G;^28^). In average, the Tat-activated HIV-1 promoter produces convoys of 19 polymerases, each polymerase spaced every ~4 s, with a convoy being fired every ~2 min. In order to characterize how a limiting amount of Tat affects the viral transcriptional output, we performed live-cell imaging using MCP-GFP and monitored the brightness of transcription sites over time. The single molecules of unspliced pre-mRNA present in the nucleoplasm were used to calibrate the signal at the transcription site, which could then be expressed as an absolute number of RNA molecules (Fig. 2). We previously showed that the Tat-activated HIV-1 promoter fluctuates on time scales ranging from minutes to hours, and we therefore recorded two types of movies to cover the entire temporal range of transcriptional fluctuations^28^. ‘Short movies’ capture one image stack every 3 s for 15–20 min, and they allow a detailed characterization of rapid transcriptional fluctuations such as polymerase convoys. ‘Long movies’ last for 8 h with a rate of one image stack every 3 min, and they allow us to measure the frequency and duration of long inactive periods. Note that since a nascent RNA resides 2.8 min at the transcription site^28^, this frame rate ensures that most initiation events are detected in the long movies.

In the short movies, we observed transient increases in the brightness of transcription sites for all three cell lines: *High Tat*, *Low Tat,* and *No Tat* (Fig. 2A–F). They were in the minute range and quantification of the signals indicated that they corresponded to the synthesis of multiple RNA molecules (Fig. 2A–F). Thus, viral transcription occurred in large bursts even in the absence of Tat, resulting in the formation of polymerase convoys. To better characterize these rapid fluctuations, we focused on transcription cycles in which an inactive transcription site transiently turned on, and we fitted these data with a model of polymerase convoys (^28^; see schematic in Fig. 2G). Surprisingly, the convoys formed in the *Low Tat* and *No Tat* cells were roughly similar to those formed when Tat was saturating: convoys had 19 polymerases in average in *High Tat* cells, with a polymerase initiating every 4 s, while convoys had 14 and 12 polymerases in *Low Tat* and *No Tat* cells, respectively, with initiation events occurring every 6 and 8 s (Fig. 2H). This result was unexpected because decreasing Tat levels should increase pausing, which should increase the lag time between successive polymerases, possibly until convoys are no longer formed. It is also interesting to note that the differences observed at this rapid timescale were small and could not account for the 30 fold difference in expression induced by Tat (Fig. 1C–K).

**Fig. 3:**
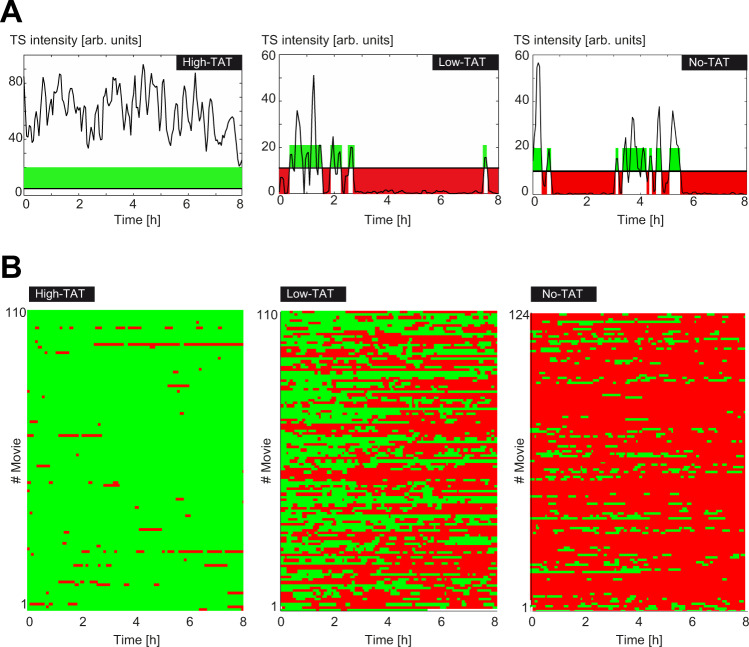
Fluctuation of HIV-1 transcription over long time periods, with and without Tat. **A** Fluctuations of HIV-1 transcription over 8 h, with one image stack recorded every 3 min. The *x*-axis represents the time (in hours) and *y*-axis represents the intensity of transcription sites, expressed in arbitrary units. Periods of HIV-1 promoter activity are colored in green, and periods of inactivity in red. **B** Active and inactive periods of the HIV-1 promoter, for the indicated cell lines. Each line is a cell and the activity of the HIV-1 promoter is color-coded (green: active; red: inactive), using the threshold shown in panel **A**. *x*-axis: time in hours. Source data are provided as a Source Data file.

Next, we analyzed fluctuations on slow time scales using long movies. The HIV-1 promoter was almost always active in cells expressing an excess of Tat (Fig. 3A, B, left panels). In contrast, *No Tat* and *Low Tat* cells displayed long inactive periods that lasted for hours (Fig. 3A, B, middle and right panels). In addition, active periods were brief and rare, yet yielded initiation of multiple polymerases in the form of convoys as for *High Tat* cells (see Fig. 3A). The activity of the HIV-1 promoter in the absence of Tat thus occurs mainly in the form of sparse, yet large bursts, with long inactive periods explaining most of the difference in promoter activity with and without Tat.

### Development of an analysis pipeline to characterize the fluctuations of promoter activity on multiple timescales

**Fig. 4:**
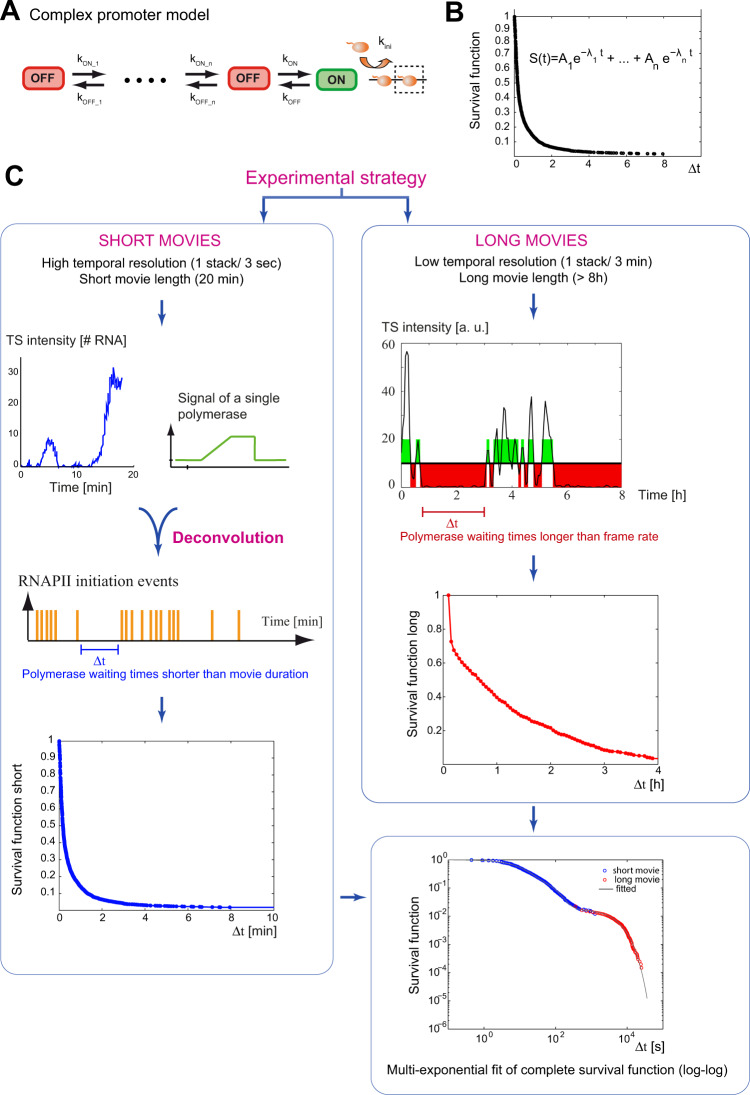
Analysis and modeling strategy for the live cell transcriptional data. **A**, **B** Determination of models for transcription initiation. **A** example of a complex multiple state promoter model, describing the different steps leading to transcription initiation and their kinetic relationship. OFF: inactive promoter state; ON: active promoter state; orange ball: RNA polymerase. **B** the survival function (equal to one minus the cumulative function) describes the distribution of polymerase waiting times (delay between two successive initiation events). For multiple state models such as the one depicted on the left, the survival function can be fitted by a sum of exponentials, with the number of exponentials being equal to the number of promoter states. **C** Experimental and machine learning strategy to determine the survival function of polymerase waiting times. Left: signals of short movies made at high temporal resolution result from the convolution of the signal from a single polymerase and the sequence of temporal positions of initiation events. The sequence of initiation events can thus be reconstructed by a deconvolution numerical method (see Supplementary Note 2), provided that the signal of a single polymerase is known. This allows us to estimate the distribution of waiting times for waiting times shorter than the movie duration (i.e. a conditional distribution). Right: long movies made with a lower temporal resolution, in the order of the residency time of RNA polymerase on the gene (3 min), allow us to estimate the distribution of polymerase waiting times for waiting times greater than the temporal resolution. The two conditional survival functions, short and long, can then be combined to reconstitute the complete, multiple time scale survival function. The reconstitution uses affine transformations of the conditional survival functions, defined by two parameters *p*_*s*_ and *p*_*l*_*. p*_*l*_ is the probability that the waiting time is larger than the frame rate of the long movie. It is proportional to the number of waiting times hidden within active periods of the long movie, and is estimated from the number of inactive intervals and the cumulative duration of active periods of the long movie (see Supplementary Note 3). *p*_*s*_ is the probability that the waiting time is larger than the short movie length and is fitted to minimize the distance between short and long parts of the distribution. Finally, the complete survival function is fitted with a sum of exponentials to determine the number of promoter state, the kinetics of transitions between them, and the initiation rate. Multiple models can be easily fitted to the same survival function and the most appropriate one is selected based on parsimony, parametric indeterminacy and consistency with complementary experiments.

Intrinsic fluctuations of promoter activity arise from stochastic transitions between active and inactive promoter states (^25,26^; Fig. 4A). These transitions correspond to steps that are kinetically rate limiting, and the characterization of these promoter states can thus yield important information on how promoters function and are regulated. To better understand how pausing and Tat control the activity of the HIV-1 promoter, we turned to machine learning and modeling approaches, with the aim of elucidating how the promoter switches between active and inactive states. The analysis of the fluctuations of transcription site brightness can be done by autocorrelation strategies^44,45^. This gives a direct measurement of the dwell time of the nascent RNAs and allows us to estimate the elongation and 3′-end processing rates. However, there is currently no theoretical framework that can easily extend autocorrelation methods to models containing multiple promoter states besides a simple ON/OFF switch. In addition, correlation approaches are difficult to use when fluctuations are slow and approach the recording time of the movies. Other analysis strategies hypothesize a theoretical transition model and infer parameters using Bayesian or maximum likelihood approaches^29,46–48^. These strategies however rarely compare several models. To circumvent these difficulties and develop a flexible approach, we turned to the analysis of polymerase waiting times, i.e. the lag time between two successive initiation events. Indeed, transcription can be modeled as a continuous time Markov chain in which a promoter stochastically switches between various non-productive states, until it reaches an active state where it can initiate transcription (Fig. 4A). In this case, waiting times between successive initiation events are interesting to consider because their distribution directly relates to transition rates of the Markov chain (see Supplementary Notes). Indeed, distinct promoter models produce predictable distributions of polymerase waiting times and we obtained for many different models closed-form equations expressing the distribution of waiting times as a function of the model parameters (for full solutions to this direct problem, see Supplementary Notes 4.1 and 4.2). In addition, for a number of promoter models, we also obtained closed-form equations allowing us to compute the model parameters directly from the distribution of waiting times, the so-called inverse problem (for full solutions, see Methods and Supplementary Note 4). In particular, if we consider a class of models containing several consecutive OFF states and one ON state that can initiate transcription (Fig. 4A), the survival function of polymerase waiting times (which is one minus their cumulative distribution), is the sum of several exponentials with the number of exponentials corresponding to the number of promoter states (Fig. 4B; see also Supplementary Note 3.3). Thus, by fitting the survival function with various sums of exponentials, one can directly determine the number of states in the promoter model. In addition, the rates of promoter switching can be directly calculated from the coefficients of the fitted exponentials (see Methods and Supplementary Note 4). Hence, if the distribution of waiting times can be extracted from the experimental data, it is straightforward to determine both the number of promoter states, as well as the rates of switching between these states.

### Calculation of polymerase waiting times from short and long movies

We first reasoned that the inactive periods seen in the long movies correspond to long polymerase waiting times (Fig. 3A, B). Since the frame rate is 3 min while a nascent RNA remains 2.8 min at the transcription site^28^, these movies should detect most initiation events and should thus measure the polymerase waiting times that are longer than 3 min. The short waiting times could be calculated from the short movies, which have a much higher frame rate (3 s). However, a difficulty is that the signal generated by a polymerase persists several minutes after it initiated, as the labeled nascent RNA leaves the transcription site only after it is transcribed to the end of the gene and 3′-end processed (see schematic in Fig. 2G). Consequently, if the next polymerase appears before the nascent RNA disappears, the transcription site remains continuously fluorescent and it is not possible to directly calculate the polymerase waiting times. To circumvent this difficulty, we reasoned that the intensity of transcription sites over time is the result of the convolution of two functions: the signal produced by a single polymerase and the time sequence of firing events (see ref. ^25^ and Fig. 4C, left panels). The signal produced by a single polymerase depends on the polymerase elongation rate and the rate of 3’-end formation, which we determined previously for this HIV-1 reporter gene^28,39^. If we assume that all polymerases behave identically, it is thus possible to calculate the temporal position of polymerase initiation events by finding the best sequence of events that reproduces the experimental transcriptional fluctuations (Fig. 4C). It should therefore be possible to extract polymerase waiting times from the short movies, keeping in mind that the waiting times longer than the movie duration will be truncated and require a correction (see Supplementary Note 3.1).

Altogether, the long movies give access to waiting times longer than the frame rate (waiting times in the 3 min–10 h range), and the short movies provide waiting times shorter than the movie length (in the 3 s–20 min range). The combination of these movies thus allows us to reconstruct and estimate the distribution of polymerase waiting times over 4 logarithms, i.e. 3 s–10 h (see Supplementary Note 3 for the reconstruction procedure). This analysis pipeline has three advantages. First, by determining the number of exponentials required to fit the survival function, one can directly determine the number of promoter states in the model. Second, given that the theoretical distribution of waiting times can be obtained for a number of models, it is straightforward to fit many models and compare them. Finally, this pipeline enables to combine data acquired at multiple timescales, from seconds to ten hours, and therefore provides an ideal framework to quantify transcriptional dynamics in live cells.

### Validation of the analysis pipeline by simulations

**Fig. 5:**
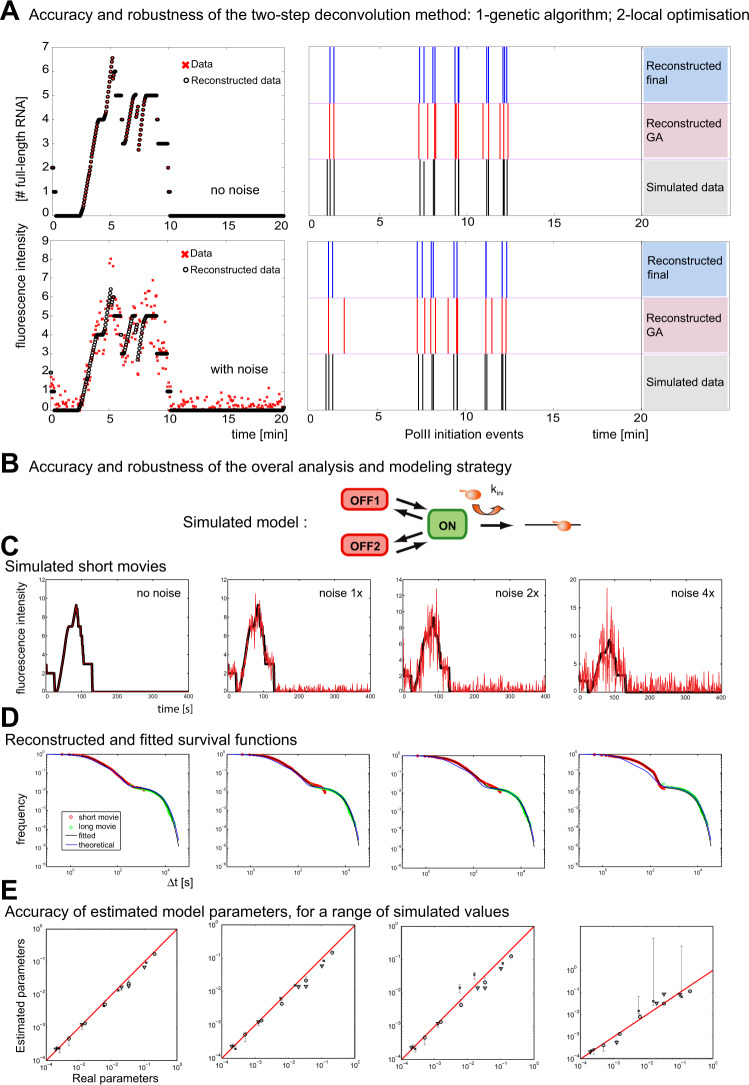
Accuracy and robustness of the analysis and modeling pipeline. **A** Accuracy and robustness of the deconvolution method. Left panels: simulation of short movies for an artificial set of polymerase initiation events, with noise added (bottom), or without (top). *x*-axis is time in minutes; *y*-axis is the intensity of transcription sites (expressed in number of RNA molecules). Right panels: positions of the transcription initiation events (vertical bars), for the original artificial data (black; bottom lines), the reconstructed data from the simulated short movies after the genetic algorithm (GA, red, middle lines), or the final reconstruction after both the GA and the local optimization (blue; top lines). *x*-axis is time in minutes. **B**–**E**. Accuracy and robustness of the overall analysis pipeline. **B** The linear three-state promoter model used for Monte Carlo simulations. **C** Examples of artificial short movies (black lines), with various levels of noise added (red lines). Note that the experimentally measured noise level (resulting from the fitting deviations) corresponds to the 1x condition. *x*-axis is time in seconds; *y*-axis is the intensity of transcription sites expressed in number of RNA molecules. **D** Survival functions reconstructed from artificial short and long movies (red and green circles, respectively), and fitted to a sum of three exponentials (black line). The theoretical survival function obtained with the model parameters used for the simulation is shown for comparison (blue line). *x*-axis: time intervals between successive initiations events, in seconds and in log_10_ scale. *y*-axis: probability of Δ*t* > *x* (log_10_ scale). **E** Accuracy of determining the model parameters. Graphs plot the parameters used to generate the artificial data (*x*-axis), against the parameter measured by the deconvolution and fitting procedure (*y*-axis). Vertical bars: confidence intervals estimated during the fitting procedure (see Methods). Three parameter sets were used, corresponding to the values obtained with the experimental data from the High Tat cells (circles), Low Tat cells (crosses), and No Tat cells (triangles).

To evaluate the precision and reliability of the analysis pipeline, we first tested the performance of the deconvolution algorithm on simulated datasets. The initiation times of several polymerases were simulated and the signal of an imaginary transcription site was calculated using experimentally measured elongation and 3′-end processing rates^28,39^. We then added a realistic amount of noise and tested the ability of the deconvolution algorithm to reconstruct the proper initiation timing from the noisy signal (Fig. 5A and Supplementary Note 8). This algorithm is composed of two parts: a genetic algorithm to obtain the rough position of initiation events, and a local optimization to refine the position of initiation events. In both presence and absence of noise, the deconvolution algorithm allowed an accurate positioning of the initiation events (Fig. 5A).

Next, we validated the entire analysis pipeline by simulating a three-state branched promoter model with the Gillespie algorithm (Fig. 5B), using several realistic sets of parameters (i.e. corresponding to values obtained with our cell lines, see below). We computed the brightness of many imaginary transcription sites as above and added different amounts of noise (1x, 2x, and 4x), with the 1x condition corresponding to the noise observed in our experimental data (Fig. 5C, see also Supplementary Note 8). The intensities of the simulated transcription sites were then resampled to create artificial short and long movies, which were treated exactly as real data. Simulated short movies were deconvolved and the distribution of waiting times was computed separately for the short and long movies. These distributions were then combined to reconstruct the entire distribution of waiting times (Fig. 5D), which was fitted to a sum of three exponentials to calculate the parameters of the promoter model. In the absence of noise, all the model parameters were recovered accurately and with high precision (i.e. a small error interval, see Methods and Supplementary Notes 3.3 and 8), for the three sets of parameter values used to generate the artificial data (Fig. 5E). With the 1x and 2x amount of noise, parameter recovery was still accurate, while for the 4x noise condition, some parameters were recovered with a low precision, in particular those corresponding to rapid transition rates. Overall, these simulations indicated that our analysis pipeline worked well, even with complex promoter models, and was robust with respect to noise.

### Modeling indicates that pausing is stochastic and that pauses are long-lived

**Fig. 6:**
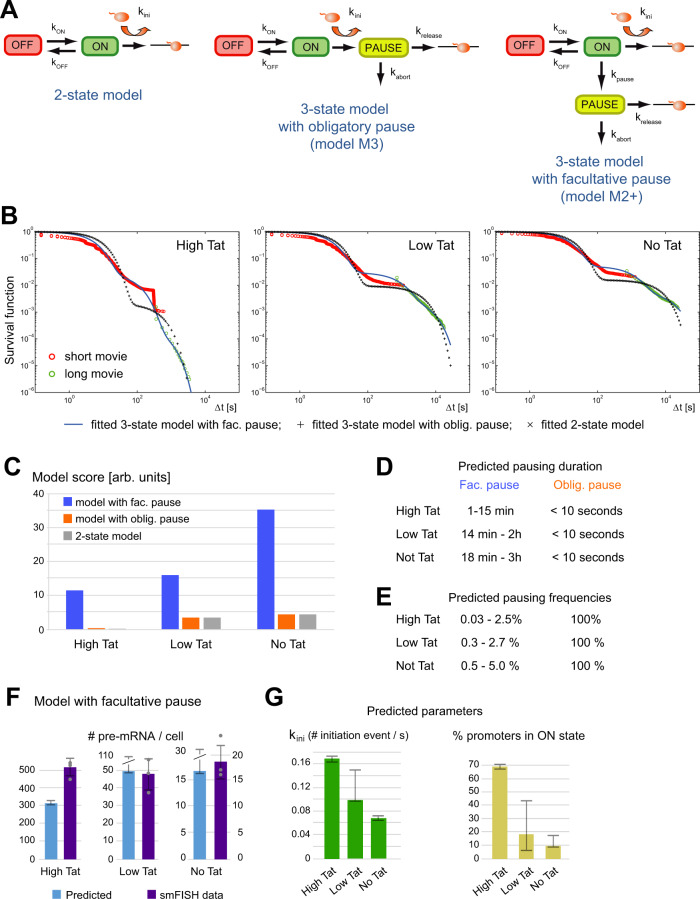
A facultative pausing model reproduces the live cell transcription data and predicts a long-lived pause. **A** Schematics of the different models used to fit the live cell HIV-1 transcriptional data. Polymerases are represented by small orange balls. **B** Fits of the experimental survival functions. Graphs represent the survival functions reconstructed from the live cell data for the High Tat, Low Tat, and No Tat conditions, with the part deriving from the short and long movies in red and green, respectively. Blue line: fit of the 3-state model with a facultative pause; “+“: fit of the 3-state model with an obligatory pause; “x”: fit with a facultative pause. *x*-axis: time intervals between successive initiations events, in seconds and in log_10_ scale. *y*-axis: probability of Δ*t* > *x* (log_10_ scale). **C** Model scores. The graph depicts the score of each model (inverse of the minimal value of the fitted Objective Function), for each of the model and cell line. **D**, **E** Pausing characteristics predicted by the models. **D** Predicted pausing times, for the relevant models and cell lines (see text for details). **E** Predicted pausing frequencies (in %), for the indicated cell line and model. For the model with the facultative pause and systematic abortion, the two indicated values come from the two branches of the model that could each correspond to the paused state (see the symmetric representation of the model M2 when *k*_release_ = 0 in Supplementary Notes Fig. S5). **F**, **G** Features of the model with the facultative pause. **F** The graphs represent the number of mRNA per cell measured by smFISH experiments (violet bars), or predicted from the model parameters (blue bars, with the center being the best fit value predicted from the model). Error bars are the standard deviation for the smFISH data (estimated from independent measurements; *n*= 3 for *High Tat* and *Low Tat* cells, and *n* = 4 for *No Tat* cells) and the confidence intervals for the prediction from the model (see Methods). Source data are provided as a Source Data file. **G** Estimated initiation rate (in s^−1^), for the three cell lines (left), and the fraction of the cells with the promoter in the ON state (in %; right). The center is the best fit value predicted from the model and error bars are confidence intervals estimated during the fitting procedure (see Methods and Supplementary Note 3.3). Source data are provided as a Source Data file.

We analyzed the movies produced from cells expressing different amounts of Tat and created several models describing how the HIV-1 promoter may operate. The simplest model has two promoter states, ON and OFF as shown in Fig. 6A, and assumes that once initiated, RNAPII enters directly into productive elongation without a pausing step. This would likely be the case when the expression of Tat is high and pausing is not rate limiting, but not when Tat is limiting or absent. We thus created a model that included a pausing step. It consisted of the same simple model with two promoter states (OFF and ON), but with initiating polymerases undergoing an obligatory pause (PAUSE), before either progressing into elongation or aborting (Fig. 6A; model M3). Note that once the polymerase exits the pause or aborts, the promoter goes immediately back in the ON state. A large body of work indicates that Tat is promoting elongation by recruiting P-TEFb and in agreement, P-TEFb is limiting for HIV-1 transcription in the *Low Tat* and *No Tat* cells used here (Fig. S1B, C). Therefore, we expected to have a high abortion rate (*k*_abort_) and/or a low rate of pause release (*k*_release_) in the absence of Tat, and the opposite when Tat is abundant. Conversely, the rates of switching between the ON and OFF states should not be much affected by the amount of Tat.

For the obligatory pausing model, the symbolic solution describing the distribution of polymerase waiting times is the sum of three exponentials, but with one of the five parameters being constrained and expressed as a function of the others (see Supplementary Note 4.6). After fitting the experimental distributions of polymerase waiting times using this symbolic solution, we estimated the quality of the fit with three criteria: (i) the sum of squared residuals, evaluated from the function minimized during the fit (i.e. the objective function, with the inverse of its minimal value giving the fit score); (ii) the certainty of the value of the fitted model parameters, evaluated by their error intervals; (iii) the realistic nature of the parameter values, and in particular the pausing times and the effects of Tat. According to these considerations, the fit of the three-state model with an obligatory pause was poor, and this was the case of all the Tat cell lines. First, the model scores were low and not better than the simple 2 state model without pause, even in the *Low Tat* / *No Tat* cell lines where P-TEFb recruitment limits viral transcription (Fig. 6B, C). Second, the uncertainty in some parameter was high, as shown by the large error intervals of the parameters of the fitted exponentials (see Supplementary Notes Table S5). Third, the pausing time, estimated from the rates of pause release and transcription abortion, was short (Fig. 6D; less than 10 s whether Tat was present or not), while most of the regulation induced by Tat occurred at the transition between the ON and OFF state and not pausing (see Supplementary Notes Fig. S21). It is also interesting to note that the fitted abortion rate was found to be > 100 fold faster than the rate of pause release (Fig. S21). Because the promoter goes directly to the ON state upon abortion or pause release, a high abortion or release rate creates a collapse between the ON and PAUSE states and therefore simplifies the three-state model with pause into a simple 2 state ON/OFF model without pause. This explains why these two models have identical scores and fitted survival functions (Fig. 6B, compare curves with ‘+‘ and ‘x’). In order to try improving the model with obligatory pause, we made a four-state model having two successive OFF states, one ON state and an obligatory pause (Model M4; see Supplementary Notes Fig. S22). This model fitted the data better and had a better overall score (see the value of the objective function in Supplementary Notes Table S6). However, it suffered from similar flaws as the previous model (Supplementary Notes Fig. S24): (i) short pausing time whether Tat was present or not (<10 s); and (ii) high abortion rates, which similarly collapsed the four-state model with an obligatory pause into a three-state model without pause. Overall, increasing the number of OFF states in the model with an obligatory pause still yields short pausing times not regulated by Tat. Thus, an obligatory pause does not provide a benefit over a model without pause, with most of the effect of Tat occurring at the level of transitions between OFF and ON states. It is important to realize that given the high degree of bursting without Tat, with polymerases rapidly succeeding one another during periods of gene activity (Fig. 2), an obligatory pause necessarily means that pausing is short. In addition, since Tat mainly affects long inactive periods (Fig. 3B), short pauses mean that the regulation by Tat cannot be on pausing, but rather on other steps able to produce long OFF periods. Hence, the occurrence of polymerase convoys in the absence of Tat implies that an obligatory pause cannot be the step regulated by Tat to increase transcription.

This questioned the validity of the model and we thus sought for alternatives. In the previous models, pausing is an obligatory step, but it could be imagined that pausing is a facultative step, for instance if entry into the pause is stochastic. In this case, initiating polymerases have the choice of either directly progressing into productive elongation or entering a paused state, from which they can exit by either aborting or entering elongation (Fig. 6A, model M2+). To test this model, we first used a simplified variant of this model, in which polymerases systematically abort when exiting a facultative pause (*k*_release_ = 0 in model M2+; thereafter referred to as model M2; see Supplementary Notes Fig. S5). This model could fit the data from all the three cell lines, *High Tat*, *Low Tat,* and *No Tat* (Fig. 6B), with scores higher than the three- or four-state models with an obligatory pause (Fig. 6C; Supplementary Notes Tables S3 and S6). Moreover, all parameters had a high precision with small error intervals (Table S3; Fig. S4), and the model correctly predicted the number of pre-mRNA per cell (Fig. 6F), with only a slight under-estimation for the *High Tat* cells. The fitted parameters indicate that pausing is infrequent, even in cells lacking Tat (Fig. 6E). This implies that the fate of the paused polymerase will only marginally affect the promoter output, indicating that models M2+ in which the paused polymerase enters productive elongation would give similar results. Because the simplified model M2 is symmetrical (Supplementary Notes Fig. S5), it is not possible to determine with certainty which parameters correspond to the ON–OFF transition, and which correspond to the ON-facultative pause. Nevertheless, both possibilities indicate a long pausing time from 15 min to 3 h in *No Tat* cells, which is regulated by Tat as it decreases to either 1 or 15 min in *High Tat* cells. Pausing is also always predicted to be infrequent, varying from one every 20–180 polymerases in *No Tat* cells, down to one every 40–3900 in *High Tat* cells (Fig. 6E).

### Measurement of pausing duration by biochemical approaches

**Fig. 7:**
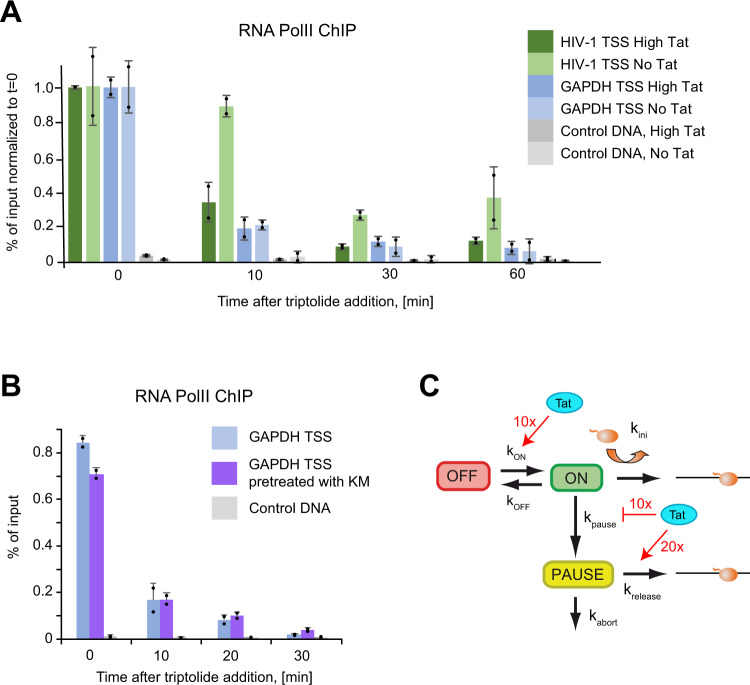
Biochemical measurements indicate a long-lived paused state at the HIV-1 promoter. **A** Residency time of RNA polymerase II at the HIV-1 promoter. The graph depicts the RNA polymerase II ChIP signals at the HIV-1 and GAPDH promoters during a Triptolide time course experiment, for the High Tat and No Tat cell lines. GAPDH TSS: transcription start site of the human GAPDH gene; HIV-1 TSS: transcription start site of the HIV-1 promoter; Control DNA: a non-transcribed genomic locus. ChIP signals were measure by qPCR and values are expressed as percent of input and normalized to the zero time point. For the control genomic regions (Control DNA), values are normalized to that of GAPDH TSS at time zero. Values are averaged from two independent experiments (+/- standard deviation) and source data are provided as a Source Data file. **B** Effect of pTEFb inhibition on the residency time of RNA polymerase II at the GAPDH promoter. Legend as in panel A, except that the KM sample was pretreated with the Cdk9 inhibitor KM05382 for 2 h before triptolide addition. Values are averaged from two independent experiments (±standard deviation) and source data are provided as a Source Data file. **C** Model depicting the dynamics of the HIV-1 promoter and highlighting the positive and negative effects of Tat. The numbers are from the facultative pausing model fitted to the High Tat and No Tat data (see Fig. 6C and S4; Supplementary Notes Table S4). The model with facultative pausing has two symmetrical branches (see model M2 in the Supplementary Notes Figure S5), and each branch of the model could correspond to the paused state. The values indicated attribute the pause state to the branch that is most affected by the presence of Tat.

To further assess models with obligatory or stochastic pausing, we attempted to test their most discriminative prediction. Obligatory pausing predicts a pausing time in the second range, while facultative pausing predicts a duration in the hour or sub-hour range (Fig. 6D). Pausing duration can be estimated by measuring RNAPII residency time at promoters, and this can be achieved by performing chromatin immunoprecipitation (ChIP) during a time-course with Triptolide, a drug that inhibits TFIIH and prevents loading new polymerases without removing the ones that already initiated. We treated *High Tat* and *No Tat* cells with Triptolide for up to an hour and performed an RNAPII ChIP experiment. We analyzed the HIV-1 promoter as well as the GAPDH promoter as a constitutively active control gene (Fig. 7A). In the *High Tat* cells, similar levels of RNAPII were found on both the GAPDH and the viral promoters, while about 6-fold less polymerases were found on the HIV-1 promoter in the absence of Tat, consistent with previous results (Fig. S3;^49^). Most importantly, treatment with Triptolide led to the rapid disappearance of RNAPII at the GAPDH promoter, with only ~20% of the signal remaining after 10 min of treatment (Fig. 7A). Interestingly, the kinetics observed at the HIV-1 promoter was dependent on Tat. In *High Tat* cells, the RNAPII signal also decreased rapidly and this was consistent with the rapid succession of polymerase firing that we measured in live cells (one every 4–6 s ^28^). In contrast, the polymerases remained associated a much longer time with the viral promoter in the absence of Tat, with 88% of the signal remaining after 10 min of treatment (Fig. 7A). Extrapolation of the half-life of the promoter-associated polymerases indicated 10 min for the GAPDH promoter and for the HIV-1 promoter when Tat levels are high. However, this half-life raised to 38 min for the HIV-1 promoter when Tat was absent, consistent with a long pause. These long values may moreover be underestimated as hour-long treatment with Triptolide were shown to cause degradation of RNA polymerase II in human cells^50^. Altogether, these data verify a key discriminative prediction of the facultative pausing model, namely that paused polymerases exhibit a half-life in the sub-hour range and not in the second range as expected from an obligatory pausing scenario.

Next, we wished to determine whether long pausing time requires a specific feature of the HIV-1 promoter or could be induced at any promoter by depleting P-TEFb. We thus repeated the GAPDH RNAPII ChIP time course, but pretreated cell with the Cdk9 inhibitor KM05382 for 2 h before performing the Triptolide time course. The residency time of RNAPII at the GAPDH promoter was similarly short whether cells were pretreated with KM05382 or not (Fig. 7B), indicating that the lack of P-TEFb activity is not sufficient in itself to induce long pauses. This suggests that the HIV-1 promoter likely has additional features that specify this property.

### Latently infected HeLa cells display bursts of viral transcription

**Fig. 8:**
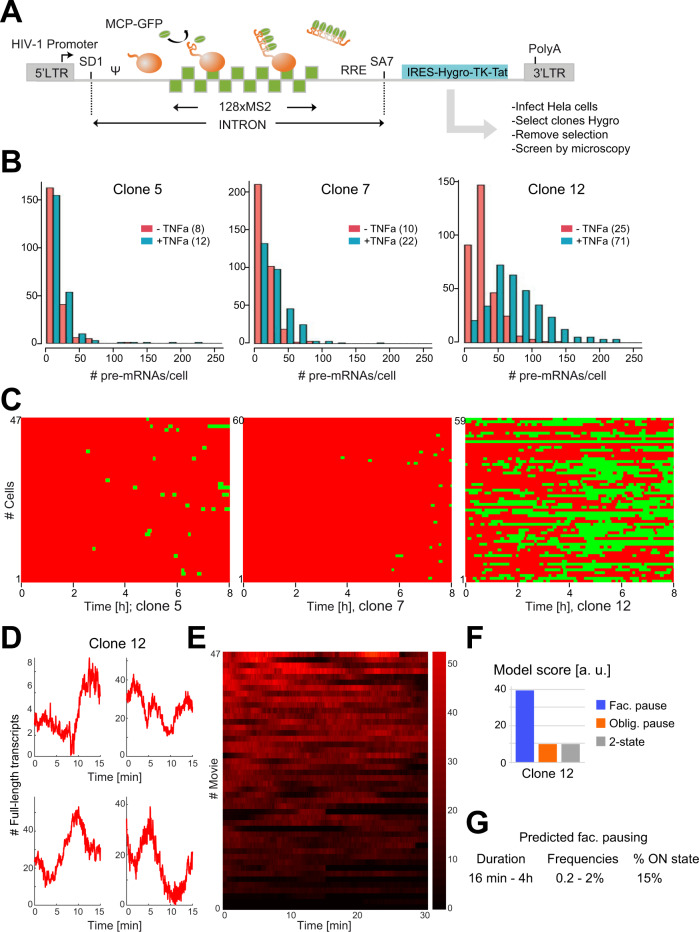
Bursting of the HIV-1 promoter in latently infected HeLa cells. **A** Schematic of the HIV-1 reporter construct used to generate latent cells. SD1: major HIV-1 splice site donor; SA7: last HIV-1 splice site acceptor; ψ: packaging signal; RRE: Rev-responsive element; LTR: long terminal repeat; IRES: internal ribosome entry site; Hygro: hygromycin selectable marker; TK: herpes simplex thymidine kinase counter selectable marker. **B** Expression of HIV-1 in three latently infected HeLa clones. The histograms represent the distribution of the number of released HIV-1 128xMS2 pre-mRNAs per cell, in each of the three clones. Experimental RNA distributions are from smFISH data. *x*-axis: number of HIV-1 pre-mRNA molecules per cell; y-axis: number of cells. Red bars: untreated cells; blue bars cells incubated with TNFα (50 ng/ml for 30 min); inset: cell treatment, with the mean number of HIV-1 pre-mRNAs per cell indicated in parenthesis. Source data are provided as a Source Data file. **C** Active and inactive periods of the HIV-1 promoter, for the indicated cell lines. Each line is a cell and the activity of the HIV-1 promoter is color-coded (green: active; red: inactive), using the threshold shown in Fig. S4. *x*-axis: time in hours. **D**, **E** Fluctuations of HIV-1 transcription over 15–30 min periods, with one image stack recorded every 3 s in cells from the clone 12. **D** each graph is a single transcription site; the x-axis represents the time (in minutes) and *y*-axis represents the intensity of transcription sites, expressed in equivalent numbers of full-length pre-mRNA molecules. **E** Each line is a cell and the transcription site intensity is color-coded (scale on the right). Source data are provided as a Source Data file. **F** Model scores. The graph depicts the score of each model (inverse of the minimal value of the fitted Objective Function), for clone 12 and for each of the model of Fig. 6A. **G** pausing characteristics predicted by the model of facultative pausing for the clone 12. The two indicated values come from the two branches of the model that could each correspond to the paused state (see Fig. S4).

In order to test the generality of the findings made with *Low Tat* and *No Tat* cells, we generated HeLa cells latently infected with a 128xMS2-tagged HIV-1 virus. The HIV-1 reporter was modified to introduce an Hygro-TK-Tat cassette in the second exon, which was translated from an internal ribosome entry site (IRES; Fig. 8A). This way, transduced clones can be selected on hygromycin and acutely infected cells can be counter-selected on ganciclovir, which kills TK expressing cells. We transduced HeLa cells with a VSV-G pseudotyped 128xMS2 HIV-1 reporter and selected three clones (see Methods) that were further characterized in detail: clones 5, 7, and 12. Tat expression was not detected in either clone, confirming their latent status (Fig. S4). SmFISH experiments indicated a basal level of viral transcription in all clones (Fig. 8B). In clone 12, the basal expression level was similar to the one observed in No Tat cells, and treatment with TNFα indicated that viral transcription was nicely induced by this cytokine (3 fold activation in 30 min), which is known to trigger viral expression. In clone 5 and 7, basal transcription was 2.5-3 fold lower and viral transcription was poorly activated by TNFα, with 1.5 and 2 fold activation, respectively. Previous studies in T cells have shown that latent HIV-1 virus display large variations in induction by TNFα and that this correlates with chromatin status and RNAPII regulation^51^. Clones 5, 7, and 12 therefore represent a diversity of situations as can be found in latent T cells. Next, we imaged HIV-1 transcription in these three clones using the conditions for long movies (8 h with an image stack every 3 min; Fig. 8C). We found that clone 12 was very similar to *No Tat* and *Low Tat* cells, with long periods of viral inactivity interspersed with bursts of viral transcription lasting tens of minutes (30 min. on average, Fig. S4A, B). Clone 5 and 7 also displayed bursts of viral transcription. However, these were much less frequent and shorter (10 min on average) and the promoter was mostly inactive (Fig. 8C and Fig. S4A, B). This shows that latent cells display bursts of viral transcription in the absence of any stimulation, and that different integration sites can display a diversity of bursting dynamics. We then imaged latent cells using the short movie conditions (one stack every 3 s for 30 min), focusing on clone 12 because the rarity of viral transcription in clone 5 and 7 limited data acquisition. Clone 12 displayed transient increases of transcription site brightness that were characteristic of polymerase convoys (Fig. 8D, E). We then subjected the short and long movies of clone 12 to our entire data analysis and modeling pipeline (Fig. 8F, G and S4C-S4E). As in the case of *Low Tat* and *No Tat* cells, the model with facultative pausing was the best fitting model. In addition, the model parameters of clone 12 were also very similar to the ones of *No Tat* and *Low Tat* cells, with pausing time ranging from 16 min to 4 h, and pausing frequencies from 0.2 to 2%. Altogether, these data demonstrate that HeLa cells latently infected with HIV-1 display rare bursts of viral transcription, which are best explained by stochastic pausing.

## Discussion

Cells latently infected with HIV-1 prevent patients from clearing the virus, as the stochastic activation of these cells can re-establish viral propagation^32,34^. Latent cells do not express the viral genome and pausing of RNA polymerases at the viral promoter is a key block that prevents HIV-1 transcription^35–38^. Pausing thus plays a fundamental role in HIV-1 biology, and yet, how it contributes to bursting and stochastic reactivation of the virus is not known. Here, we harnessed the power of single-molecule transcriptional imaging and modeling to study how pausing affects HIV-1 transcription in single cells. We found that pausing is a stochastic process, and modeling as well as biochemical experiments indicate that it is long-lived inhibitory state that impacts only a small fraction of the initiating polymerases. Stochastic pausing therefore generates viral transcriptional bursts in the absence of Tat. Similar bursts are observed in latently infected HeLa cells, which also express little or no Tat. In patients, this may cause stochastic viral reactivation, latency exit, and viral rebounds.

Single-molecule transcription imaging is a powerful technique that becomes indispensable for understanding transcriptional regulation in vivo. However, the signals produced by this technique integrate processes with widely distributed timescales, not directly accessible by simple data processing. Hence, new modeling methods are needed to cope with the multiscale nature of transcription. To this end, we developed a machine learning and modeling method. Using numerical deconvolution, this approach generates a time map of transcriptional initiation events indicating, for each transcription site, when RNAPII molecules start producing an RNA. This feature is unique to our analysis pipeline and not available in other approaches that directly fit a particular transcription model to experimental data^29,45–48^. Our method generates a multiscale cumulative distribution function of polymerase waiting times (i.e., times that separate two successive transcription initiation events). This distribution function has the unique advantage of integrating temporal information on transcriptional processes with an unprecedented dynamic range from seconds to days. Moreover, we have analytically solved the inverse problem consisting in computing the model parameters as a function of the waiting time distribution, for a large number of models. By allowing easy and quick comparison of many different models of promoter dynamics, this method removes a bottleneck that is essential for hypothesis testing in gene regulation studies.

P-TEFb is an essential elongation factor that is required for both the basal and Tat-induced activity of the HIV-1 promoter^11,35,36^. By default, the HIV-1 promoter leads to pausing and inefficient elongation, and Tat functions as a promoter-specific elongation factor by recruiting P-TEFb to the nascent viral RNAs. When Tat is present in saturating concentrations, we observe that polymerases initiate rapidly one after another (every 4–6 s in average^28^). This indicates that the maturation of initiating polymerases into a processive complex is rapid, in agreement with the fact that P-TEFb recruitment is not rate limiting when Tat is abundant. When Tat is limiting or absent, we observe a biphasic behavior. HIV-1 promoters are mostly inactive, and yet sometimes transcribe the viral genome in brief pulses containing tens of polymerases. These polymerases are fired in rapid succession (one every 7–15 s), and they form convoys resembling the ones observed when Tat is saturating. Modeling the imaging data in *Low Tat* and *No Tat* cells confirms this biphasic behavior and further indicates that in average, 20–35 polymerases initiate during active periods of 5 min, followed by inactive periods of 20 min to 3 h. Given that pausing is limiting transcription in the absence of Tat, these long inactive periods are likely caused by long-lived pauses at the HIV-1 promoter. Indeed, direct measurements of RNAPII residency time at the HIV-1 promoter indicate that the absence of Tat generates long pauses, in the sub-hour range, which are therefore responsible for some of the long periods without viral transcription.

Recent genome-wide data obtained in *Drosophila* and mammalian cells with Triptolide time-course experiments indicate that the half-life of polymerases at cellular promoters varies from less than a minute to about an hour^19–23^. Analysis of a series of promoter variants further indicates that an initiator element with a G at position +2 is a key determinant of long pausing time (>40 min ^52^). It is not known whether this rule also applies to vertebrates, but it is worth noting that the HIV-1 promoter has an unusual initiator element required for Tat activation that contains a G at +2^53^. Moreover, inhibiting P-TEFb does not generate long pauses at the GAPDH promoter, suggesting that some promoter-specific features exist. In the future, it will be interesting to determine whether long-lived and short-lived paused polymerases have a similar 3D structure. Indeed, recent data in NELF KO cells suggests that polymerases can have several pausing sites and states^54^. Because of their half-life, long-lived paused polymerases may display additional features such as backtracking or other stabilizing properties, and in this regard it is interesting to note that backtracking was indeed shown to occur at the HIV-1 pause site^55^. Long-lived paused polymerases are especially interesting because of their properties, which effectively limit transcription but maintain the promoter in an open state^22^.

In the traditional model of transcription initiation, polymerase pausing is an obligatory step during the formation of the elongation complex^2,56^. In contrast, our live cell data on HIV-1 transcription suggest that pausing is a stochastic event that occurs rarely: 1 every 20–180 polymerase in the absence of Tat and down to 1 every 40–3900 when Tat is abundant. A model reconciling these views would be the existence of two fates during pausing: a pause could lead to either rapid enzyme maturation, or to a long-lived inactive state that would inhibit further transcription. In this scenario, the polymerases initiating at the HIV-1 promoter would mature into a processive elongation complex but would have a low probability of entering a long-lived paused state (Fig. 7C). A key feature of this model is that long-lived pauses are stochastic, and this changes the nature of this process as long-lived pauses would not be a step required for proper polymerase maturation but an inhibitory state preventing transcription. In essence, stochastic long-lived pauses are analogous to an inactive promoter state (Fig. 7C). In the case of HIV-1, long-lived pauses would be a key regulatory step in transcriptional regulation. By ensuring an efficient recruitment of P-TEFb, Tat would drastically reduce the probability of long inhibitory pauses (see model in Fig. 7C). This is consistent with the fact that the HIV-1 promoter is fully occupied in a model of latent cells^57^, even if in some cases Tat can slightly enhance PIC occupancy^49^. It is also consistent with the known function of Tat as a P-TEFb/SEC recruiting factor, with a major function in reducing pausing.

The basal activity of the HIV-1 promoter requires P-TEFb and it is surprising that the factors responsible for P-TEFb recruitment in the absence of Tat allow for the firing of a series of polymerases before switching back to a long inactive state. Indeed, the HIV-1 promoter is active for periods of ~ 5 min in the absence of Tat, firing 20 polymerases on average. A possibility to explain this behavior would be a switching mechanism, in which P-TEFb would be present and active for several minutes at the HIV-1 promoter, and then leave for long time periods. Our data show that NF-κB is not involved in the basal transcriptional activity of HIV-1 in our cellular system, and we can thus rule out sporadic activation of this pathway as a cause of transcriptional bursts in the absence of Tat. Another possibility would involve the diffusion dynamics of P-TEFb. Indeed, it has been shown that P-TEFb is a local explorer that repetitively visits the same location^58^, and recent data further suggest that P-TEFb undergoes transient liquid-liquid phase transitions^59^. FRAP studies showed that the residency time of P-TEFb is 11 s at the HIV-1 promoter in the absence of Tat^60^, and 55 s at the transcription site of a CMV-based reporter^59^. While this is too short to explain the 5 min active periods without Tat, single-particle tracking of P-TEFb subunits indicate a wide range of binding times^59^. Moreover, P-TEFb also might exchange rapidly from long-lasting liquid condensates. It is also possible that other phenomena are responsible for P-TEFb recruitment, or that long pauses arise from an inherently stochastic and inefficient process.

The stochastic nature of long-lived polymerase pausing and their low probability has important consequences for HIV-1 pathogenesis. There are evidences that the stochastic activation of the viral promoter is responsible for the stochasticity of latency exit, at least in part^32–34,37^. Moreover, latent viruses do not express Tat or at very low levels^35,36^, and we show that in these conditions the spontaneous release of a long-lived pause leads to the synthesis of a large series of viral RNAs. In some cases, this may be sufficient to activate the viral promoter and to initiate the Tat positive feedback loop, leading to acute viral replication. The stochastic nature of long-lived pausing may thus be an important feature of HIV-1 regulation that favorizes spontaneous latency exit^34,37,38^. It is also possible that even if the viral RNAs produced do not initiate the Tat-feedback loop, they may still produce a small amount of viral particles, which may infect naive cells and could thus participate in the viral rebounds or viremia blips seen in patients. It is also important to note that quiescent memory T cells have a low P-TEFb activity^35,36,61,62^, possibly leading to very long periods without HIV-1 transcription. In the future, it will be essential to characterize the bursting dynamics of the HIV-1 promoter in latent T cells to better understand viral latency. It will be especially interesting to compare quiescent and dividing T cells as they differ in their levels of P-TEFb activity. Finally, stochastic pausing has also been reported in developing *Drosophila* embryos, where it may finely tune gene expression after zygotic genome activation^63^. Stochastic pausing may be a general property of cellular promoters important for gene regulation.

## Methods

### Cell culture and drug treatments

HeLa Flp-in H9 cells (a kind gift of S. Emiliani) were maintained in DMEM supplemented with 10% fetal bovine serum, penicillin/streptomycin (10 U/ml) and glutamine (2.9 mg/ml), in a humidified CO_2_ incubator at 37 °C. Cells were transfected with the indicated plasmids with JetPrime (Polyplus), following manufacturer recommendations. Drugs were used at the following concentrations: Triptolide, 1 μM; KM05382 100 μM; BAY11-7082, 2 μM.

Stable expression of MCP-GFP was achieved by lentivirus-mediated transduction of a self-inactivating vector containing an internal ubiquitin promoter driving MCP-GFP expression. The MCP contained the deltaFG deletion, the V29I mutation, and an SV40 NLS. MCP-GFP expressing cells were grown as a pool of clones and FACS-sorted to select cells expressing low levels of fluorescence. Isogenic stable cell lines expressing the 128xMS2 HIV-1 reporter gene were created using the Flp-In system, with a HeLa H9 strain expressing various levels of Tat (see below) and MCP-GFP. Flp-In integrants were selected on hygromycin (150 μg/ml). For each construct, several individual clones were picked and analyzed by in situ hybridization.

*No Tat* cells expressed the 128xMS2 HIV-1 reporter gene but did not express any Tat protein. To obtain low level of Tat expression, a Tat-Flag fused to an Auxin-inducible degron (AID) and cloned as a second cistron after auxin receptor F-box protein AFB2 and instead of GFP in the vector AAV-CAGGS-eGFP^64^. The resulting vector was integrated in the genomic AAVS1 site using CRISPR-Cas9 and clones were selected using puromycin as described^64^. Cells were not treated with Auxin.

*High Tat* cells^28^ were created using the plasmid pSpoII-Tat. In this plasmid, the CMV promoter transcribes a Tat-Flag cDNA followed by an IRES-Neo selectable marker. Following Neomycin selection (400 μg/ml), expression levels of individual clones were verified by western blotting and by immunofluorescence to ensure homogeneity both between clones and between cells of a clone.

### Production of latently infected HeLa cells and immunofluorescence

To produce VSV-G pseudotyped viral stocks, 293T cells were transfected with plasmids pHDM-Hgpm2, pHDM-Tat1b, pRC-CMV-Rev, pHDM-VSV-G, and HIV-1 reporter containing 128xMS2 and IRES-hygro-TK-Tat. The viral supernatants were collected 24 and 48 hours after transfection and used to infect Hela flp-in cell line in the presence of 6 μg/ml of polybrene. Three days after infection, hygromycin at concentration 100 μg/ml was added to select transduced clones. The selected clones were then cultivated without selection to let the HIV-1 promoter switch to a latent state and clones further screened by smFISH. Several clones with low expression of the 128xMS2 HIV-1 RNAs and undetectable levels of Tat were found, and we therefore did not use the TK/ganciclocir counter-selection. These clones were then transduced with the MCP-GFP encoding lentiviral vector and used for live cell imaging.

Immunofluorescence against Tat-FLAG was performed by fixing cells grown on coverslips in 4% formaldehyde/PBS for 20 min at room temperature. Cells were washed in PBS and permeabilized in Triton 0.5%/PBS at RT for 20 min, and washed thrice. Slides were blocked for 30 min at RT in BSA 1% in PBS, and immunolabeling was performed with anti-FLAG antibodies (Sigma F7425), diluted 1/300 in PBS/BSA1%, for 1 h at RT, and washed for 3 times 10 min in PBS at RT. Secondary antibody labeling was done by incubating slides with anti-rabbit-Cy5 antibodies (1/800; Jackson ImmunoResearch 711-175-152) for 2 h at RT, and washing in PBS for 3 times 10 min at RT.

### Plasmids

Sequences of the plasmids are available upon request. The 128xMS2 HIV-1 reporter and High Tat expression vector were described previously^28^. The 128xMS2-IRES-Hyrgo-TK-Tat vector was generated by cloning an IRES-Hygro-TK-Tat cassette in the second exon of the 128xMS2 HIV-1 vector. For the AAV-CAGGS-eGFP vector used to obtain low Tat cells, Cas9 encoding vector and AAVS1-site targeting RNA guides were obtained from Dr. G. M. Church^64^. pcDNA3-CDK9-GFP and pcDNA3-CyclinT1-GFP plasmids were obtained by Gateway technology, CDK9 and Cyclin-T1 were amplified by PCR from the vectors provided by Dr. L. Lania^65^. pHR-SFFV-dCas9-BFP plasmid used for CDK9 cloning is #46911 from Addgene. The RNA guides were cloned in a home-made U6 expression vector with an optimized guide RNA scaffold^66^.

### dCas9 tethering and pTEFb overexpression

For P-TEFb overexpression, Hela 128xMS2 HIV-1 No Tat cells without MCP-GFP were plated on coverslips and the next day transfected with CDK9-GFP, Cyclin-T1-GFP, or both, using jetprime (polyplus). pBluescript was used as a negative control and GFP-Tat as a positive control. 24-h after transfection cells were fixed and the reporter RNA was detected by smFISH with Cy3-labeled fluorescent probes against MS2 repeats, the RNA expression was scored in transfected GFP-positive cells.

For CDK9 tethering, RfB gateway cassette was cloned in pHR-SFFV-dCas9-BFP between dCas9 and BFP. CDK9 was next introduced by LR recombination. The resulting plasmid pHR-SFFV-dCas9-CDK9-BFP was transfected in Hela *No Tat* cells without MCP-GFP together with three RNA guides encoding plasmids as described above. pHR-SFFV-dCas9-CDK9-BFP without guides and pHR-SFFV-dCas9-BFP were used as controls. 24 h after transfection cells were fixed and subjected to smFISH with probes against 128xMS2. The numbers of RNA molecules in BFP-positive cells were counted using FISH-QUANT^67,68^. The sequences of RNA guides were as follows CCGCCTAGCATTTCATCACG, CCACGTGATGAAATGCTAGG, TGCTACAAGGGACTTTCCGC.

### SmFISH and RNA quantification

For smFISH cells were rinsed in PBS, fixed in 4% paraformaldehyde for 30 min, permeabilized in 70% ethanol O/N at 4 °C, and hybridized with a mix of 10 fluorescent oligos directed against the 32xMS2 repeats. Each oligo contained four molecules of Cy3. Hybridization was performed O/N at 37 °C in a humidified chamber, in the following buffer: 40% formamide, 1xSCC, 150 ng/μl tRNA *E. coli*, 0.5 ng/μl of oligo probes (total weight), Dextran sulfate 10%. Washing was performed for 3 times 30 min at 37 °C in 40% formamide, 2xSSC, and then once for 10 min in PBS at RT. Slides were mounted in Vectashield (Vectorlabs). The following set of ten Cy3 conjugated probes were used (5′–3′, X = aminoallyl-modified T). Probe 1: (nt 19–65) AXCGAGCGCATAAACCCXAATGGTGTTTACAAATGGXGGTAGTCCTACCXA;

Probe 2 (nt 86–130):

AXAAACGACCAGAGXGTATTTCTCTCTGATACGCXGCGTACTCGTCAXA;

Probe 3 (nt 136–180):

AXATTGTGCGGTCGCXGACTGATACTTCTAGXCATCCGTTTGTCTAGXA;

Probe 4 (nt 186–231):

AXGCTTGTAGTCAXAGCCTTAGCTTGGGTTATTACXCCAAGATCACCGXA;

Probe 5 (nt 238–282):

AXGGGTGGAAGCCTTACXGATGCTTCCGGTCCATXCTAATACTATGGXA;

Probe 6 (nt 293–340):

AXCCAGTAGTCTTGGACCCCXTGAATACTACTGTTATTCXAATCCGTCACXA;

Probe 7 (nt 29–74):

AXCCTAGTGGTACGCAGAXCATACCGTATTCGTGTAXGATTACATGGGXA;

Probe 8 (nt 80–124):

AXAATCATTCTAGTGAXATGATTCTGTGCCGCXACTGCTGGCACCGTXA;

Probe 9 (nt 130–175):

AXCGTCCTGATAGGCXGTACTCATGCCTACAACCXTCGATAATTCTGAXA;

Probe 10 (nt 184–229):

AXGTGTATTCATCTXAGCTGAGTGCTCTAAXGATGCACTACAGGACGCXA.

To obtain the number of nascent and released pre-mRNAs per cell and the distribution of this parameter in the cell population, cells processed for smFISH were imaged on a ZEISS Axioimager Z1 wide-field microscope (63X, NA 1.4; 40X, NA 1.3), equipped with an sCMOs Zyla 4 .2 camera (Andor) and controlled by MetaMorph (Universal Imaging). To quantify the number of released pre-mRNA in *High Tat* cells, cells were imaged on an OMX microscope in SIM mode. 3D image stacks were collected with a Z-spacing of 0.3 μm. Figures were prepared with Image J, Photoshop and Illustrator (Adobe), and graphs were generated with R or MatLab.

Raw, 3D smFISH images were analyzed to count the number of pre-mRNA per nuclei, using populations of >300 cells per experiment. Briefly, nuclei were segmented using the DAPI signal with Imjoy^69^, and transcription sites (TS) were identified manually. Isolated pre-mRNA molecules located in the nucleoplasm were then detected with *FISH-quant*^67,68^, after manual thresholding of Laplacian on Gaussian filtered image. This defined the PSF and the total light intensity of single molecules, which were averaged to obtain an average PSF. The average PSF of single RNA molecule was used to determine the number of nascent pre-mRNA molecules at the TS.

### Live cell imaging

Cells were plated on 25 mm diameter coverslips (0.17 mm thick) in non-fluorescent media (DMEM gfp-2 with rutin; Evrogen). Coverslips were mounted in a temperature-controlled chamber with CO_2_ and imaged on an inverted OMXv3 Deltavision microscope in time-lapse mode. A 100x, NA 1.4 objective was used, with an intermediate 2X lens and an Evolve 512 × 512 EMCCD camera (Photometrics). Stacks of 11 to 21 planes with a z-spacing of 0.6 μm were acquired. This spacing still allowed accurate PSF determination without excessive oversampling. Illuminating light and exposure time were set to the lowest values that still allowed visualization of single molecules of pre-mRNAs (laser at 1% of full power, exposure of 15 ms per plane). This minimizes bleaching and maximizes the number of frames that can be collected. Yet, it guarantees that transcription can be detected early on, when one or a few nascent chains are in the process of being transcribed. For short movies, one stack was recorded every 3 s for 15 to 20 min. For long movies, one stack was recorded every 3 min for 8 h.

### Quantification of short movies

To extract the TS signal in the short movies, we manually defined the nuclear outline and the region within which the TS is visible. The stack was corrected for photobleaching by measuring the fluorescence loss of the entire nucleus and fitting this curve with a sum of three exponentials^28^. This fitted curve was then used to renormalize each time-point such that its nuclear intensity was equal to the intensity of the first time-point. We then filtered the image with a 2-state Gaussian filter. First, the image was convolved with a larger kernel to obtain a background image, which was then subtracted from the original image before the quantification is performed. Second, the background-subtracted image was smoothened with a smaller Kernel, which enhances the SNR of single particles to facilitate spot pre-detection.

We then pre-detected the position of the TS in each frame of the filtered image by determining in the user-specified region the brightest pixel above a user-defined threshold. If no pixel was above the threshold, the last known TS position was used. Pre-detected position was manually inspected and corrected. Then the TS signal was fitted with a 3D Gaussian estimating its standard deviation *σ*_*xz*_ and *σ*_*z*_, amplitude, background, and position. We performed two rounds of fitting: in the first round all fitting parameters were unconstrained. In the second round, the allowed range was restricted for some parameters, to reduce large fluctuations in the estimates especially for the frames with a dim or no detectable TS. More specifically, the *σ*_*xz*_ and *σ*_*z*_ were restricted to the estimated median value ± standard deviation from the frames where the TS could be pre-detected, and the background was restricted to the median value. The TS intensity was finally quantified by estimating the integrated intensity above background expressed in arbitrary intensity units.

With the live cell acquisition settings, the illumination power was low and we could not reliably detect all individual molecules. We therefore collected right after the end of the movie one 3D stack—termed calibration stack—with increased laser intensity (50% of max intensity, compared to 1% for the movie), which allowed reliable detection of individual RNA molecules. We also collected slices with a smaller z-spacing for a better quantification accuracy (21 slices every 300 nm). Quantification of TS site intensity in the calibration stack was done with *FISH-quant* as follows: (a) when calculating the averaged image of single RNA molecules, we subtracted the estimated background from each cell to minimize the impact of the different backgrounds; (b) when quantifying the TS in a given cell, we rescaled the average image of single RNA molecules such that it had the same integrated intensity as the molecules detected in the analyzed cell.

To calibrate the TS intensities in the entire movie, i.e. to express the TS intensity as a number of equivalent full-length transcripts, we used the fact that the last movie frame was acquired at the same time as the calibration stack. We then normalized the extracted TS intensity in the movies, *I*_MS2_, to get the nascent counts *N*_nasc;calib_:

1 $${N}_{{\rm{nasc;calib}}}(t)={I}_{MS2}(t)\;\times\; ({N}_{{\rm{nasc,final}}}/{I}_{{\rm{final}}}),$$

where *N*_nasc,final_ stands for the estimated number of nascent transcripts in the calibration stack and *I*_final_ for the averaged intensity of the last four frames. Note that the approach was limited to movies where the TS was active at the movie end since otherwise its intensity could not be quantified. More than 100 cells were used in each condition.

### Quantification of long movies

To quantify the long movies acquired at low frames rate (one 3D stack per 3 min), we used *MS2-quant*^28^, an analysis tool that identifies the ON and OFF periods and measures their duration. This did not require an absolute quantification of the number of nascent pre-mRNAs and we therefore defined an intensity threshold, based on the mean intensity of single molecules, under which a TS is considered to be silent, and above which a TS is considered to be active. This threshold corresponded to the intensity of 1.5 pre-mRNA. For each cell line between 50 and 150 cells were analyzed.

### Mathematical modeling of fluctuations of promoter activity and deconvolution pipeline

A detailed description of the deconvolution algorithm and modeling pipeline can be found in the Supplementary Notes. The deconvolution code is available at Zenodo DOI: 10.5281/zenodo.4811566) and Github (https://github.com/oradules/Deconvolution_short_long for the complete version using both short and long movies; and at https://github.com/oradules/Deconvolution_short for the version using only short movies).

*Deconvolution and RNAPII Positioning*—The RNAPII positions were found by using a genetic algorithm followed by a local optimization procedure. Before initiation of the analysis algorithm, several key parameters were established. The RNAPII elongation speed was fixed at 67 bp/s^28^. The reporter construct transcript was divided into three sections consisting of the pre-MS2 fragment (PRE = 700 bp), 128xMS2 loops (SEQ = 2900 bp), and post-MS2 fragment (POST = 1600 bp). An extra time *P*_poly_ = 100 s was added to POST, corresponding to the time required for cleavage/polyadenylation (during this time the polymerase has finished transcription of the gene and continues in neighboring sequences to wait for RNA 3′-end processing). The temporal resolution of short movies was 3 s/frame. This frame rate is sufficient to detect processes that occur on the order of seconds.

The possible polymerase positions were discretized using a step of 30 bp. This step was chosen as it is smaller than the minimum polymerase spacing and large enough to have a reasonable computation time. For a movie of 20 min length this choice corresponds to a maximum number of 2680 positions. The deconvolution algorithm was implemented in Matlab (Matlab, 2020, version 9.8 (R2020a)). Natick, Massachusetts: The Mathworks Inc.) using Global Optimization and Parallel Computing Toolboxes, for optimizing RNAPII positions in parallel for all nuclei in a collection of movies. The resulting positions are stored for analysis in the further steps of our computational pipeline. The deconvolution step developed here is also used in another MS2 data analysis pipeline that uses only short movies and that we designed for the study of time-limited processes in developmental biology^63^.

*Long movies waiting time distribution*—For long movies, the low resolution (3 min) does not allow RNAPII positioning. In this case we binarize the signal by considering that the transcription site is active or inactive if the measured intensity is above or below a threshold level, respectively. The inactive intervals indicate long waiting times between successive polymerases. The active intervals are used to estimate the probability that waiting times are larger than the movie resolution, needed for the reconstruction of the multiple time scale distribution from the short and long movies distributions (see Supplementary Note 3).

*Multi-exponential regression fitting of the survival function and model reverse engineering using the survival function*—Data from several short movies corresponding to the same cell lines were first pooled together. Waiting times were extracted as differences between successive RNAPII positions from all the resulting traces and the corresponding data was used to estimate the nonparametric cumulative short movie distribution function by the Meyer-Kaplan method. Data from long movies and the same cell lines were also pooled to generate the nonparametric cumulative long movie distribution function. The two conditional distribution functions are fitted together into a multiscale cumulative distribution function using the total probability theorem and estimates of two parameters *p*_*l*_ and *p*_*s*_, representing the probabilities that waiting times are longer than the long movie resolution, and longer than the length of the short movie, respectively (see Fig. 4 and Supplementary Note 3 for details).

Then, a multi-exponential regression fitting of the multiscale distribution function produced a set of 2*N*−1 distribution parameters, where *N* is the number of exponentials in the regression procedure (3 for *N* = 2 and 5 for *N* = 3). We performed the regression using a sum of squared residuals objective function. The regression procedure was initiated with multiple log-uniformly distributed initial guesses and followed by local gradient optimization. It resulted in a best-fit solution with additional suboptimal solutions (local optima with objective function value larger than the best fit).

The 2*N*−1 distribution parameters can be computed from the 2*N*−1 kinetic parameters of a *N* state transcriptional bursting model. Conversely, a symbolic solution for the inverse problem was obtained, allowing computation of the kinetic parameters from the distribution parameters and reverse engineering of the transcriptional bursting model. In particular, it is possible to know exactly when the inverse problem is well-posed, i.e. there is a unique solution in terms of kinetic parameters for any given distribution parameters.

The transcriptional bursting models used in this paper are as follows:

For *N* = 2, there were three distribution parameters and three kinetic parameters.

The distribution parameters are ${A}_{1},\, {\lambda }_{1}$, ${\lambda }_{2}$, defining the survival function

2 $$S(t)={A}_{1}{\rm{e}}^{{\lambda}_{1}t}+({1-A}_{1}){{\rm{e}}}^{{\lambda }_{2}t}.$$

The solution of the inverse problem for the ON–OFF telegraph model (Fig. 6A) is

3 $${k}_{2} 	=-{S}_{1},\,{k}_{1}^{-}={S}_{1}-\frac{{S}_{2}}{{S}_{1}},\, {k}_{1}^{+}=\frac{{S}_{3}{S}_{1}-{S}_{2}^{2}}{{S}_{1}\left({S}_{1}^{2}-{S}_{2}\right)}, {S}_{1} ={A}_{1}{\lambda }_{1}+{A}_{2}{\lambda }_{2},\,\\ {S}_{2}	={A}_{1}{\lambda }_{1}^{2}+{A}_{2}{\lambda }_{2}^{2},\, {S}_{3}={A}_{1}{\lambda }_{1}^{3}+{A}_{2}{\lambda }_{2}^{3},\,{A}_{2}={1-A}_{1},$$

where the kinetic parameters ${k}_{2},\, {k}_{1}^{+},\, {k}_{1}^{-}$ are the initiation rate, the OFF to ON and ON to OFF transition rates, respectively.

For *N* = 3, there were five distribution parameters and five kinetic parameters.

The distribution parameters are ${A}_{1},\, {{A}_{2},\, \lambda }_{1}$, ${\lambda }_{2},{\lambda }_{3}$, defining the survival function

4 $$S\left(t\right)={A}_{1}{{\rm{e}}}^{{\lambda }_{1}t}+{A}_{2}{{\rm{e}}}^{{\lambda }_{2}t}+(1-{A}_{1}-{A}_{2}){{\rm{e}}}^{{\lambda }_{3}t}.$$

The inverse problem has a unique solution for the three-state model (with facultative pause and systematic abortion) with one OFF state, one PAUSE state and one ON state (Fig. 6A and model M2 of Supplementary Notes Fig. S5). The kinetic parameter of this model are denoted as follows in Fig. 6 and Supplementary Note 4.5: $k_{\mathrm{ini}}=k_3;\, k_{\mathrm{pause}}=k^{-}_{2};\, k_{\mathrm{abort}}=k_{2}^{+};\,k_{\mathrm{release}}={\it{0}};\,k_{\mathrm{ON}}=k_{1}^{+};\,k_{\mathrm{OFF}}=k_{1}^{-}$ with

5 $${k}_{3}= -{S}_{1},\, {k}_{2}^{+}=\frac{1}{2}\left[-{L}_{1}+\frac{{S}_{2}}{{S}_{1}}-\frac{\sqrt{{\left({S}_{1}{L}_{1}-{S}_{2}\right)}^{2}-4{L}_{3}{S}_{1}}}{{S}_{1}}\right],\\ {k}_{2}^{-}= \frac{1}{2}\left[{S}_{1}-\frac{{S}_{2}}{{S}_{1}}+\frac{-{S}_{1}^{2}{L}_{1}+{S}_{1}{S}_{2}+{S}_{1}{L}_{2}-{L}_{3}+\frac{{S}_{2}^{2}}{{S}_{1}}-{S}_{3}}{\sqrt{{\left({S}_{1}{L}_{1}-{S}_{2}\right)}^{2}-4{L}_{3}{S}_{1}}}\right],\\ {k}_{1}^{+}= \frac{1}{2}\left[-{L}_{1}+\frac{{S}_{2}}{{S}_{1}}+\frac{\sqrt{{\left({S}_{1}{L}_{1}-{S}_{2}\right)}^{2}-4{L}_{3}{S}_{1}}}{{S}_{1}}\right],\\ {k}_{1}^{-}= \frac{1}{2}\left[{S}_{1}-\frac{{S}_{2}}{{S}_{1}}-\frac{-{S}_{1}^{2}{L}_{1}+{S}_{1}{S}_{2}+{S}_{1}{L}_{2}-{L}_{3}+\frac{{S}_{2}^{2}}{{S}_{1}}-{S}_{3}}{\sqrt{{\left({S}_{1}{L}_{1}-{S}_{2}\right)}^{2}-4{L}_{3}{S}_{1}}}\right],$$

where ${S}_{1}={A}_{1}{\lambda }_{1}+{A}_{2}{\lambda }_{2}+{A}_{3}{\lambda }_{3},\, {S}_{2}={A}_{1}{\lambda }_{1}^{2}+{A}_{2}{\lambda }_{2}^{2}+{A}_{3}{\lambda }_{3}^{2},\, {S}_{3}={A}_{1}{\lambda }_{1}^{3}+{A}_{2}{\lambda }_{2}^{3}+{A}_{3}{\lambda }_{3}^{3},\, {A}_{3}=1 - {A}_{1}-{A}_{2}, \, {L}_{1}={\lambda }_{1}+{\lambda }_{2}+{\lambda }_{3},\, {L}_{2}={\lambda }_{1}^{2}+{\lambda }_{2}^{2}+{\lambda }_{3}^{2},\, {L}_{3}={\lambda }_{1}^{3}+{\lambda }_{2}^{3}+{\lambda }_{3}^{3},\,{\mathrm{and}}\,{k}_{3},\, {k}_{1}^{+},\, {k}_{1}^{-},\, {k}_{2}^{+},{k}_{2}^{-}$ are the transcription initiation, OFF to ON, ON to OFF, PAUSE to ON, and ON to PAUSE rates, respectively.

The durations of the ON, OFF, and PAUSE states can be calculated as such:

6 $$T\left({{\rm{PAUSE}}}\right)=\frac{1}{{k}_{2+}},\, T\left({{\rm{OFF}}}\right)=\frac{1}{{k}_{1+}},\, T\left({{\rm{ON}}}\right)=\frac{1}{{k}_{1-}+{k}_{2-}}.$$

For this model, the steady-state probability to be in a given promoter state is

7 $${p}_{{{\rm{OFF}}}}= \frac{{{k}_{1}^{-}k}_{2}^{+}}{{{k}_{1}^{+}k}_{2}^{+ }+{{k}_{1}^{-}k}_{2}^{+ }+{k}_{1}^{+}{k}_{2}^{-}},\, {p}_{{{\rm{PAUSE}}}}=\frac{{{k}_{1}^{+}k}_{2}^{-}}{{{k}_{1}^{+}k}_{2}^{+}+{{k}_{1}^{-}k}_{2}^{+}+{k}_{1}^{+}{k}_{2}^{-}},\\ {p}_{{{\rm{ON}}}}= \frac{{{k}_{1}^{+}k}_{2}^{+}}{{k}_{1}^{+}{k}_{2}^{+}+{{k}_{1}^{-}k}_{2}^{+}+{k}_{1}^{+}{k}_{2}^{-}}.$$

The alternative three-state model with obligatory pause (Fig. 6A, also model M3 of Supplementary Notes Fig. S5) satisfies the following relation among distribution parameters (see Supplementary Note 4.6 for a proof):

8 $${A}_{1}{\lambda }_{1}+{A}_{2}{\lambda }_{2}+\left(1-{A}_{1}-{A}_{2}\right){\lambda }_{3}=0.$$

This means that only 4 and not 5 distribution parameters are free, which further constrains the three exponential fitting. In order to infer this model, a constrained fitting was performed but the bad quality of fitting recommended rejection of the model (Fig. 6B, C; see Results).

*Testing the method with artificial data*—The entire computational pipeline was tested for accuracy and robustness using artificial data. Artificial traces were generated by simulating the model using the Gillespie algorithm with parameter sets similar to those identified from data. The simulations generated artificial polymerase positions, from which a first version of the signal was computed by convolution. We have added to this version white noise whose standard deviation is a multiple of that of residuals obtained by fitting real data. The results are provided in Fig. 5 and Supplementary Note 8.

*Error intervals*—Distribution parameters result from multi-exponential regression fitting using gradient methods with multiple initial data. These optimization methods provide a best fit (global optimum) but also suboptimal parameter values. Using an overflow ratio (a number larger than one, in our case 2, defined as the ratio of the maximal allowed to optimal objective function values) to restrict the number of suboptimal solutions, we define boundaries of the error interval as the minimum and maximum parameter value compatible with an objective function less than the best fit times the overflow. Although a confidence level can be computed for the overflow ratio provided a uniform or log-uniform parametric prior, we considered that the goodness of fit derived overflow ratio is more informative than a confidence level that depends on the prior.

*mRNA levels*—Steady-state mRNA levels can be computed from the parameters of the multi-exponential fit. We showed in the Supplementary Note 7 that:

9 $${\rm{mRNA}}=-\frac{{T}_{{{\rm{mRNA}}}}}{ \mathop{\sum }\limits_{i=1}^{N} \frac{{A}_{i}}{\lambda_{i}} },$$

where *T*_mRNA_ is the mean lifetime of the mRNA. The formula is valid for all *N* and we have used *T*_mRNA_ = 45 min^28^.

### Chromatin immunoprecipitation

*High Tat* and *No Tat* HeLa cells were treated with 1 μM of triptolide at 0, 10, 30 and 60 min. *High Tat* HeLa cells were treated with 100 μM of KM05382 during 1 h followed by 1 μM of triptolide at 0, 10, 20, and 30 min. Cells were cross-linked by adding crosslinking solution (11% formaldehyde, 100 mM NaCl, 1 mM EDTA pH 8, 0.5 mM EGTA pH 8, 50 mM Hepes pH 7.8) directly to cultures (1% final) and incubated for 10 min at room temperature. Then, 250 mM final glycine was added, and cultures were incubated for 5 min at room temperature. Cells were then washed four times with cold PBS, scraped in cold PBS with Protease Inhibitor cocktail and centrifuged at 1350 × *g* for 10 min. Crude nuclei were prepared by hypotonic lysis. The pellet was resuspended in 5 mL of BufferA (50 mM Hepes pH 8.0, 85 mM KCl, 0.5% Triton-X-100, Protease Inhibitor cocktail, 1 mM PMSF), incubated on ice for 10 min and centrifuged at 1350 × *g* for 10 min. Then, the pellet was resuspended in 5 mL of BufferA′ (50 mM Hepes pH 8.0, 85 mM KCl, Protease Inhibitor cocktail, 1 mM PMSF) and centrifuged at 1350×*g* for 10 min. Finally, the pellet was resuspended in 0.9 mL of Buffer B (50 mM Tris-HCl pH 8, 1% SDS, Protease Inhibitor cocktail, 1 mM PMSF), incubated on ice for 10 min and then stored at the −80°. Pellets were sonicated at 4 °C using a Bioruptor (Diagenode) to shear the chromatin to a mean length of 300 bp by repeated cycles (16 cycles of 30 s ON and 30 s OFF). After sonication cellular debris was removed by centrifugation at 20,000 × *g* for 10 min. The chromatin solution was diluted 10-fold in FA/SDS Like buffer (50 mM Hepes KOH pH 7.5, 150 mM NaCl, 1% Triton-X-100, 0.1% Na deoxycholate, Protease Inhibitor cocktail, 1 mM PMSF) and precleared for 1 hour at 4 °C with 25 μl of protein G Dynabeads (Invitrogen). The precleared chromatin solution (1.5 × 106 cells) was incubated overnight with 50 μL of BSA-blocked protein G Dynabeads (previously bound with 3 μg of the corresponding antibody, POLII F-12 sc-55492 Lot K1516 Santacruz, during 1 h at 4 °C). Samples were washed once with FA/SDS buffer (50 mM Hepes KOH pH 7.5, 150 mM NaCl, 1% Triton-X-100, 0.1% Na deoxycholate, 1 mM EDTA, 0.1% SDS, Protease Inhibitor cocktail, 1 mM PMSF), three times with FA/SDS Buffer supplemented with 300 mM NaCl, once with washing Buffer (10 mM Tris-HCl pH 8, 0.25 M LiCl, 1 mM EDTA, 0.5% NP40, 0.5% Na deoxycholate) and once with TE Buffer. Elution was performed adding 125 μl of Elution Buffer (25 mM Tris-HCl pH 7.5, 5 mM EDTA, 0.5% SDS) and incubating at 65 °C for 25 min. The eluates were digested with 50 μg/mL of RNase A at 37 °C for 30 min and with 50 μg/ml of proteinase K at 50 °C for 1 h. Then, they were incubated at 65 °C overnight to reverse cross-links. DNA was recovered by phenol extraction followed by a Qiaquick purification (PCR purification columns, Qiagen, Germany). Specific sequences in the immunoprecipitates were quantified by real-time PCR using the primers listed below. The signal of each sample was normalized with the average signal obtained from the input of the same sample with each pair of primers used. Each experiment was done analyzing two independent biological replicates. The primers used are listed in Supplementary Table S1.

### Statistical information

Statistical information are provided in the main text, method section, figure legend, and Supplementary notes.

### Reporting summary

Further information on research design is available in the Nature Research Reporting Summary linked to this article.

## Supplementary information

Supplementary Information

Reporting Summary

## Data Availability

The data that support this study are available upon reasonable request from the corresponding authors. Source data are provided with this paper.

## Source data

Source Data

## Code availability

The deconvolution code is available at Zenodo (10.5281/zenodo.4811566) and at https://github.com/oradules/Deconvolution_short_long for the complete version using both short and long movies and at https://github.com/oradules/Deconvolution_short for the version using only short movies.

## References

[1] Schier A, Taatjes D (2020). Structure and mechanism of the RNA polymerase II transcription machinery. Genes Dev..

[2] Jonkers I, Lis J (2015). Getting up to speed with transcription elongation by RNA polymerase II. Nat. Rev. Mol. Cell Biol..

[3] Harlen KM, Chrchman LS (2017). The code and beyond: transcription regulation by the RNA polymerase II carboxy-terminal domain. Nat. Rev. Mol. Cell Biol..

[4] Fisher R (2019). Cdk7: a kinase at the core of transcription and in the crosshairs of cancer drug discovery. Transcription.

[5] Rimel J, Taatjes D (2018). The essential and multifunctional TFIIH complex. Protein Sci..

[6] Ghosh A, Shuman S, Lima C (2011). Structural insights to how mammalian capping enzyme reads the CTD code. Mol. Cell.

[7] Fant C (2020). TFIID enables RNA polymerase II promoter-proximal pausing. Mol. Cell.

[8] Narita T (2007). NELF interacts with CBC and participates in 3’ end processing of replication-dependent histone mRNAs. Mol. Cell.

[9] Vos S, Lucas Farnung L, Henning Urlaub H, Patrick Cramer P (2018). Structure of paused transcription complex Pol II-DSIF-NELF. Nature.

[10] Cheung A, Cramer P (2011). Structural basis of RNA polymerase II backtracking, arrest and reactivation. Nature.

[11] Wei P, Garber M, Fang S, Fischer W, Jones K (1998). A novel CDK9-associated C-type cyclin interacts directly with HIV-1 Tat and mediates its high-affinity, loop-specific binding to TAR RNA. Cell.

[12] He N (2010). HIV-1 Tat and host AFF4 recruit two transcription elongation factors into a bifunctional complex for coordinated activation of HIV-1 transcription. Mol. Cell.

[13] Sobhian B (2010). HIV-1 Tat assembles a multifunctional transcription elongation complex and stably associates with the 7SK snRNP. Mol. Cell.

[14] Nilson K (2015). THZ1 reveals roles for Cdk7 in co-transcriptional capping and pausing. Mol. Cell.

[15] Vos S (2018). Structure of activated transcription complex Pol II-DSIF-PAF-SPT6. Nature.

[16] Wada T, Takagi T, Yamaguchi Y, Watanabe D, Handa H (1998). Evidence that P-TEFb alleviates the negative effect of DSIF on RNA polymerase II-dependent transcription in vitro. EMBO J..

[17] Yamada T (2006). P-TEFb-mediated phosphorylation of hSpt5 C-terminal repeats is critical for processive transcription elongation. Mol. Cell.

[18] Ehrensberger AH, Kelly GP, Svejstrup JQ (2013). Mechanistic interpretation of promoter-proximal peaks and RNAPII density maps. Cell.

[19] Henriques T (2013). Stable pausing by RNA polymerase II provides an opportunity to target and integrate regulatory signals. Mol. Cell.

[20] Jonkers I, Kwak H, Lis JT (2014). Genome-wide dynamics of Pol II elongation and its interplay with promoter proximal pausing, chromatin, and exons. Elife.

[21] Buckley MS, Kwak H, Zipfel WR, Lis JT (2014). Kinetics of promoter Pol II on Hsp70 reveal stable pausing and key insights into its regulation. Genes Dev..

[22] Shao W, Zeitlinger J (2017). Paused RNA polymerase II inhibits new transcriptional initiation. Nat. Genet..

[23] Krebs AR (2017). Genome-wide single-molecule footprinting reveals high RNA polymerase II turnover at paused promoters. Mol. Cell.

[24] Chubb J, Trcek T, Shenoy S, Singer R (2006). Transcriptional pulsing of a developmental gene. Curr. Biol..

[25] Pichon X, Lagha M, Mueller F, Bertrand E (2018). A growing toolbox to image gene expression in single cells: sensitive approaches for demanding challenges. Mol. Cell.

[26] Rodriguez J, Larson D (2020). Transcription in living cells: molecular mechanisms of bursting. Annu. Rev. Biochem..

[27] Lionnet T (2011). A transgenic mouse for in vivo detection of endogenous labeled mRNA. Nat. Methods.

[28] Tantale K (2016). A single-molecule view of transcription reveals convoys of RNA polymerases and multi-scale bursting. Nat. Commun..

[29] Rodriguez J (2019). Intrinsic dynamics of a human gene reveal the basis of expression heterogeneity. Cell.

[30] Blake W (2006). Phenotypic consequences of promoter-mediated transcriptional noise. Mol. Cell.

[31] Raj A, Rifkin S, Andersen E, van Oudenaarden A (2010). Variability in gene expression underlies incomplete penetrance. Nature.

[32] Weinberger L, Burnett J, Toettcher J, Arkin A, Schaffer D (2005). Stochastic gene expression in a lentiviral positive-feedback loop: HIV-1 Tat fluctuations drive phenotypic diversity. Cell.

[33] Ho Y (2013). Replication-competent noninduced proviruses in the latent reservoir increase barrier to HIV-1 cure. Cell.

[34] Rouzine I, Razooky B, Weinberger L (2014). Stochastic variability in HIV affects viral eradication. Proc. Natl Acad. Sci. USA.

[35] Mbonye U, Jonathan Karn J (2017). The molecular basis for human immunodeficiency virus latency. Annu. Rev. Virol..

[36] Shukla A, Ramirez N, D’Orso I (2020). HIV-1 proviral transcription and latency in the new era. Viruses.

[37] Tyagi M, Pearson R, Karn J (2010). Establishment of HIV latency in primary CD4+ cells is due to epigenetic transcriptional silencing and P-TEFb restriction. J. Virol..

[38] Jiang G (2015). Synergistic reactivation of latent HIV expression by Ingenol-3-Angelate, PEP005, targeted NF-kB signaling in combination with JQ1 induced p-TEFb activation. PLoS Pathog..

[39] Boireau S (2007). The transcriptional cycle of HIV-1 in real-time and live cells. J. Cell Biol..

[40] Fusco D (2003). Single mRNA molecules demonstrate probabilistic movement in living mammalian cells. Curr. Biol..

[41] Yedavalli VS, Benkirane M, Jeang K (2003). Tat and trans-activation-responsive (TAR) RNA-independent induction of HIV-1 long terminal repeat by human and murine cyclin T1 requires Sp1. J. Biol. Chem..

[42] Barboric M, Nissen R, Kanazawa S, Jabrane-Ferrat N, Peterlin B (2001). NF-kappaB binds P-TEFb to stimulate transcriptional elongation by RNA polymerase II. Mol. Cell.

[43] West M, Lowe A, Karn J (2001). Activation of human immunodeficiency virus transcription in T cells revisited: NF-kappaB p65 stimulates transcriptional elongation. J. Virol..

[44] Larson D, Zenklusen D, Wu B, Chao J, Singer RH (2011). Real-time observation of transcription initiation and elongation on an endogenous yeast gene. Science.

[45] Desponds J (2016). Precision of Readout at the hunchback Gene: Analyzing Short Transcription Time Traces in Living Fly Embryos. PLoS Comput Biol..

[46] Coulon A, Larson D (2016). Fluctuation analysis: dissecting transcriptional kinetics with signal theory. Methods Enzymol..

[47] Corrigan A, Tunnacliffe E, Cannon D, Chubb J (2016). A continuum model of transcriptional bursting. Elife.

[48] Lammers N (2020). Multimodal transcriptional control of pattern formation in embryonic development. Proc. Natl Acad. Sci. USA.

[49] D’Orso I, Frankel AD (2010). RNA-mediated displacement of an inhibitory snRNP complex activates transcription elongation. Nat. Struct. Mol. Biol..

[50] Vispé S (2009). Triptolide is an inhibitor of RNA polymerase I and II-dependent transcription leading predominantly to down-regulation of short-lived mRNA. Mol. Cancer Ther..

[51] Wong VC (2018). NF-kB-chromatin interactions drive diverse phenotypes by modulating transcriptional noise. Cell Rep..

[52] Shao W, Alcantara S, Zeitlinger J (2019). Reporter-ChIP-nexus reveals strong contribution of the *Drosophila* initiator sequence to RNA polymerase pausing. Elife.

[53] Rittner K, Churcher HJ, Gait MJ, Karn J (1995). The human immunodeficiency virus long terminal repeat includes a specialised initiator element which is required for Tat-responsive transcription. J. Mol. Biol..

[54] Aoi Y (2020). NELF regulates a promoter-proximal step distinct from RNA Pol II pause-release. Mol. Cell.

[55] Palangat M, Landick R (2001). Roles of RNA:DNA hybrid stability, RNA structure, and active site conformation in pausing by human RNA polymerase II. J. Mol. Biol..

[56] Wissink EM, Ihervaara A, Tippens ND, Lis JT (2019). Nascent RNA analyses: tracking transcription and its regulation. Nat. Rev. Genet.

[57] Demarchi F, D’Agaro P, Falaschi A, Giacca M (1992). In vivo footprinting analysis of constitutive and inducible protein-DNA interactions at the long terminal repeat of human immunodeficiency virus type 1. J. Virol..

[58] Izeddin, I. et al. Single-molecule tracking in live cells reveals distinct target-search strategies of transcription factors in the nucleus. *Elife* **3**, e02230 (2014). 10.7554/eLife.02230 PMC4095940 24925319

[59] Lu H (2018). Phase-separation mechanism for C-terminal hyperphosphorylation of RNA polymerase II. Nature.

[60] Molle D (2007). A real-time view of the TAR:Tat:P-TEFb complex at HIV-1 transcription sites. Retrovirology.

[61] Garriga J (1998). Upregulation of cyclin T1/CDK9 complexes during T cell activation. Oncogene.

[62] Ghose R, Liou LY, Herrmann CH, Rice AP (2001). Induction of TAK (cyclin T1/P-TEFb) in purified resting CD4(+) T lymphocytes by combination of cytokines. J. Virol..

[63] Pimmett, V. L. et al. Quantitative imaging of transcription in living Drosophila embryos reveals the impact of core promoter motifs on promoter state dynamics. *Nat. Commun.*10.1038/s41467-021-24461-6 (2021). 10.1038/s41467-021-24461-6 PMC8302612 34301936

[64] Mali P (2013). RNA-guided human genome engineering via Cas9. Science.

[65] Majello B, Napolitano G, Giordano A, Lania L (1999). Transcriptional regulation by targeted recruitment of cyclin-dependent CDK9 kinase in vivo. Oncogene.

[66] Chen B (2013). Dynamic imaging of genomic loci in living human cells by an optimized CRISPR/Cas system. Cell.

[67] Tsanov N (2016). smiFISH and FISH-quant—a flexible single RNA detection approach with super-resolution capability. Nucleic Acids Res..

[68] Mueller F (2013). FISH-quant: automatic counting of transcripts in 3D FISH images. Nat. Methods.

[69] Ouyang W, Mueller F, Hjelmare M, Lundberg E, Zimmer C (2019). ImJoy: an open-source computational platform for the deep learning era. Nat. Methods.

