# Supplementary material for: Stochastic pausing at latent HIV-1 promoters generates transcriptional bursting
Source: Nat Commun. 2021 Jul 23;12:4503. doi: 10.1038/s41467-021-24462-5 (PMC8302722; doi:10.1038/s41467-021-24462-5)
Supplement: Supplementary file 1 — Supplementary Information [file 41467_2021_24462_MOESM1_ESM.pdf]

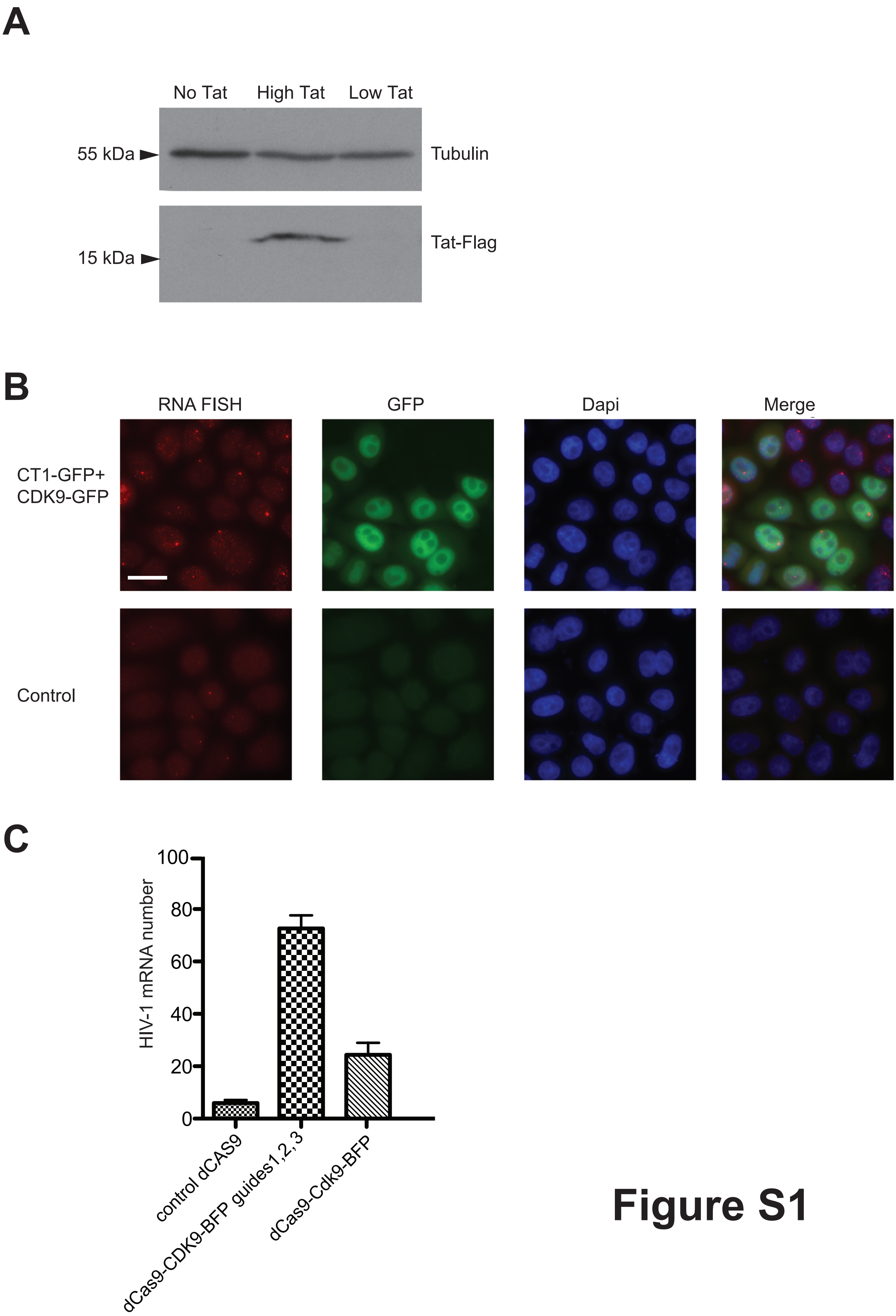
A
No Tat
High Tat
Low Tat
55 kDa
Tubulin
15 kDa
Tat-Flag
B
RNA FISH
GFP
Dapi
Merge
CT1-GFP+
CDK9-GFP
Control
C
HIV-1 mRNA number
100
80
60
40
20
0
control dCAS9
dCas9-CDK9-BFP guides1,2,3
dCas9-Cdk9-BFP

Figure S1

Supplementary Figure S1. Transcriptional activation of HIV-1 128xMS2 reporter in Hela Flp-in cells is P-TEFb dependent.

A- Western blot of the extracts of HIV-1 128xMS2 Hela cell lines with no, low and high Tat expression. Tat-Flag was detected with anti-Flag antibodies; loading control is tubulin. This experiment has been done three times with similar results. Source data are provided as a Source Data file.

B- CDK9-GFP and cyclinT1-GFP activate transcription of the HIV-1 reporter. Fluorescent microscopy images of Hela Flp-in cells with the HIV-1 128xMS2 reporter, not expression Tat nor MCP-GFP, and co-transfected with plasmids encoding for CDK9-GFP and cyclinT1-GFP (24h after transfection). First row from the left: RNA of HIV-1 reporter detected by smFISH with Cy3 probes against 128xMS2 tag; second row: GFP signal corresponding to the cells transfected with CDK9-GFP and cyclinT1-GFP; third row: nuclear staining with dapi; last row: merge. Top panel: cells transfected with CDK9-GFP and cyclinT1-GFP. Bottom panel: control transfection with pBluescript. The scale bar is 10 μm. This experiment has been done twice with similar results.

C- Tethering of CDK9 to the HIV-promoter using dCas9 leads to transcriptional activation. The histogram shows the results of mRNA counting on smFISH images 24h after transfection the Hela Flp-in HIV-1 128xMS2 no Tat cells (without MCP-GFP) with dCas9-CDK9-BFP fusion and 3 RNA guides targeting the CDK9 fusion specifically to the HIV-1 promoter (middle bar); dCas9-CDK9-BFP fusion without guides (right bar) or dCas9-BFP alone (left bar) were transfected in control experiments. The y axis represents the mRNA copy number. Error bars are standard errors of the mean and n is the number of cells quantified (27, 36 and 13 for dCas9, dCas9-CDK9+guide RNAs, and dCas9-CDK9, respectively). Source data are provided as a Source Data file.

A

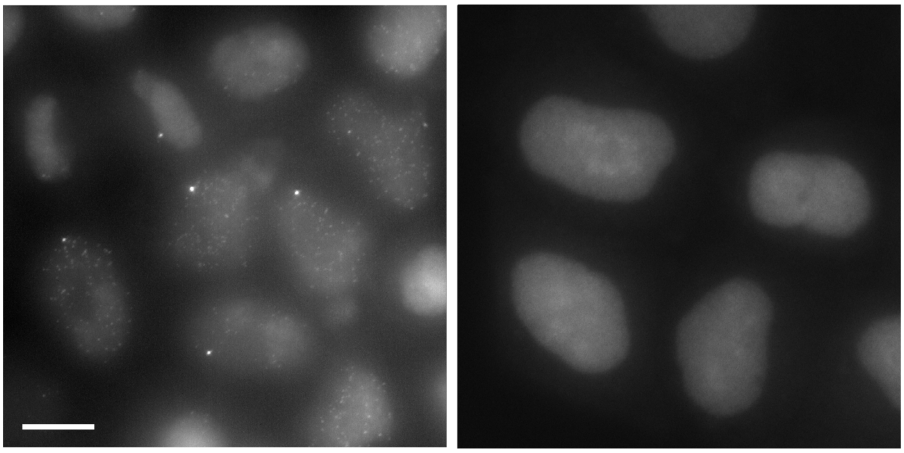

No Tat, -KM

No Tat,+KM, 4h

B

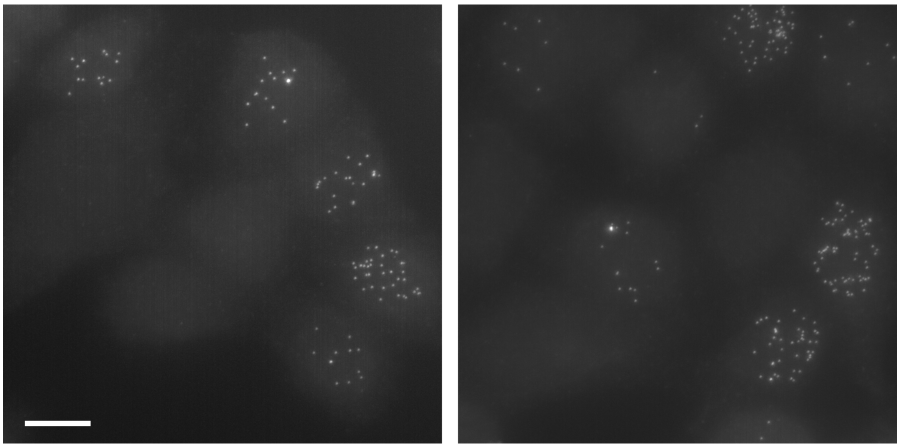

No Tat, control

No Tat, +Bay, 16h

NF-kB inhibition in no Tat cells

C

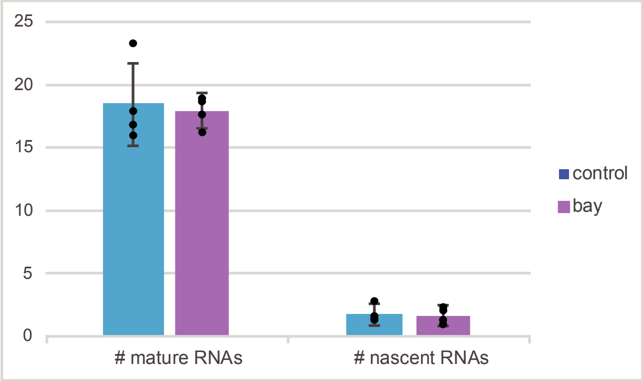

NF-kB inhibition in no Tat cells

Figure S2

Supplementary Figure S2. Transcriptional activation of HIV-1 reporter in the absence of Tat depends on enzymatic activity of CDK9 and is independent of NF-κB pathway.

A- CDK9 inhibitor KM05283 inhibits HIV-1 transcription. Images of Hela Flp-in HIV-1 128xMS2 MCP-GFP no Tat cells treated with 100 μM KM05382 for 4 h, using GFP filter. Left – non-treated control; right – 4 hours of KM05382 treatment. The scale bar is 10 μm. This experiment has been performed three times with similar results.

B- NF-κB inhibitor BAY11-7082 does not affect HIV-1 reporter transcription. Left panel: Images of smFISH with Cy3 labeled probes of the cells Hela Flp-in HIV-1 128xMS2 MCP-GFP no Tat. Left - non-treated control; right -16h treatment with 2 μM BAY11-7082. The scale bar is 10 μm. This experiment has been performed twice with similar results.

C- Histogram showing the quantification of mature and nascent RNA number on the smFISH images after 16h inhibition of NF-kB with 2 μM BAY11-7082. On y axis is the RNA number. Error bars are standard deviations from technical replicates (n=4). Source data are provided as a Source Data file.

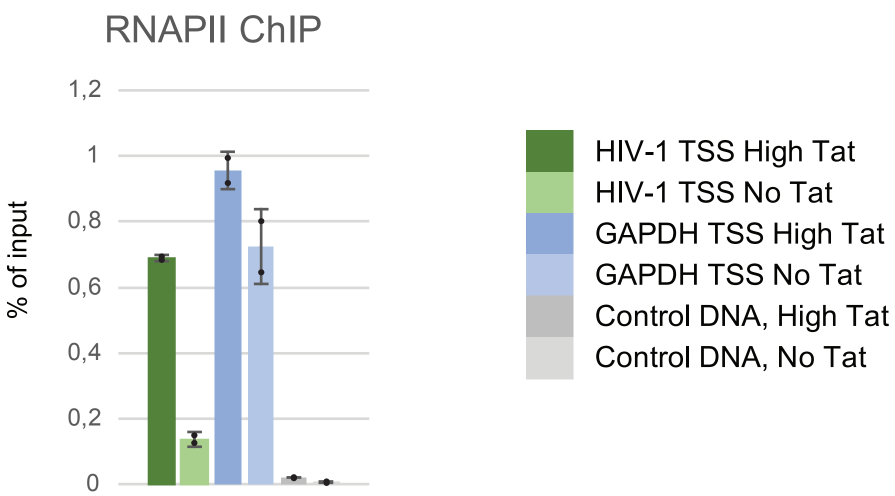
RNAPII ChIP
% of input
1,2
1
0,8
0,6
0,4
0,2
0
HIV-1 TSS High Tat
HIV-1 TSS No Tat
GAPDH TSS High Tat
GAPDH TSS No Tat
Control DNA, High Tat
Control DNA, No Tat

Figure S3

Supplementary Figure S3. RNA Pol II ChIP in presence and absence of Tat.

The graph depicts the RNA polymerase II ChIP signals at HIV-1 and GAPDH loci for the High Tat and No Tat cell lines. GAPDH TSS: transcription start site of the human GAPDH gene; HIV-1 TSS: transcription start site of the HIV-1 promoter; Control DNA: a non-transcribed genomic locus. ChIP signals were measure by qPCR and values are expressed as percent of input (y axis). Values are averaged from two independent experiments and source data are provided as a Source Data file.

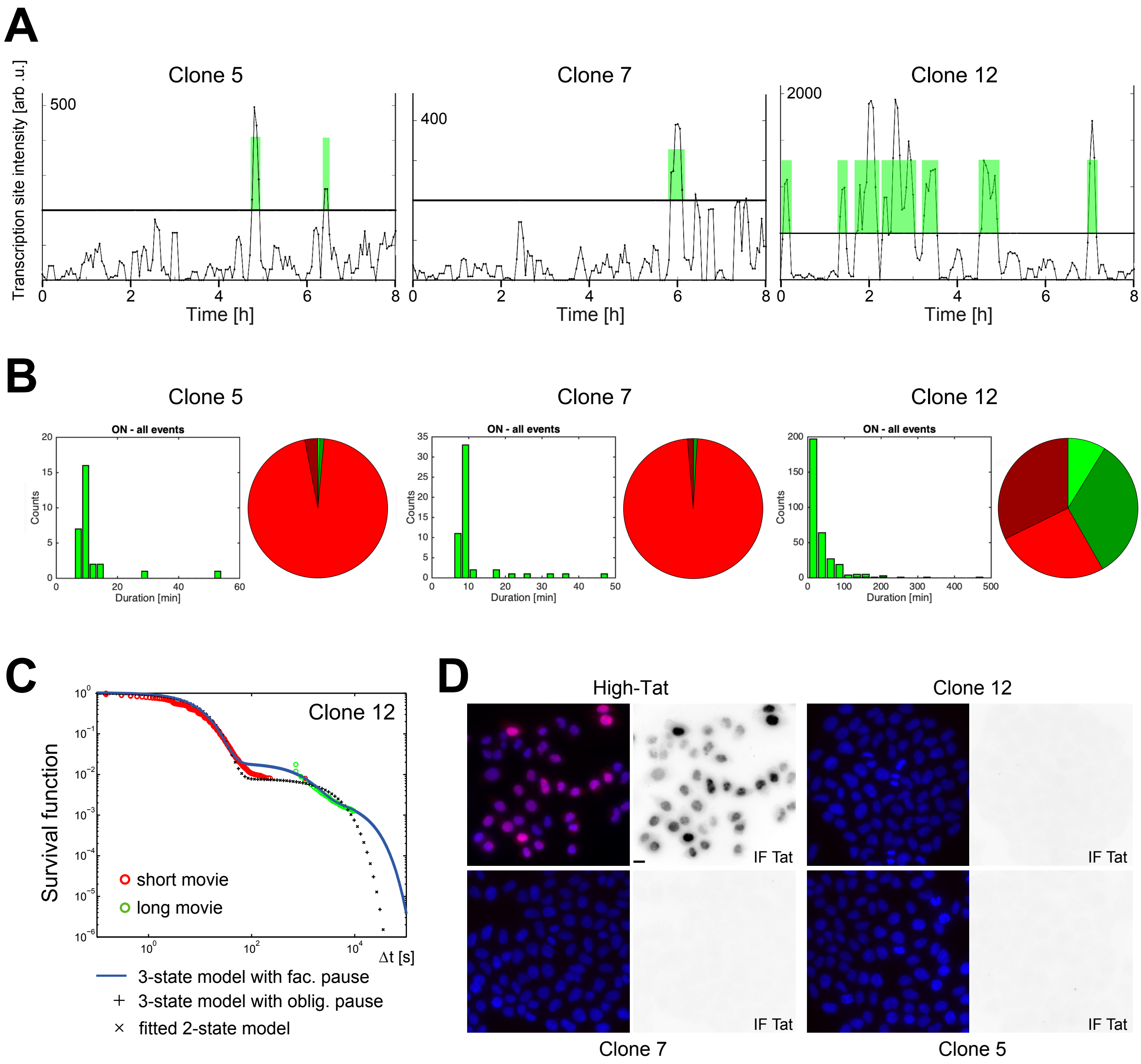

E

Cell Line	kini (m^{-1})	k1p (h^{-1})	k2p (h^{-1})	k1m (h^{-1})	k2m (h^{-1})	% ON	% OFF1	% OFF2	RNA predict.	RNA obs.
Low Tat	6.0	0.5	4.3	1.1	10.1	19	38	43	50	48
High Tat	10.2	4.0	35.3	0.1	14.4	69	2	28	319	538
No Tat	3.8	0.2	3.2	1.3	10.8	10	57	33	17	18
Clone 12	6.0	0.2	3.6	0.9	5.8	16	59	25	43	24

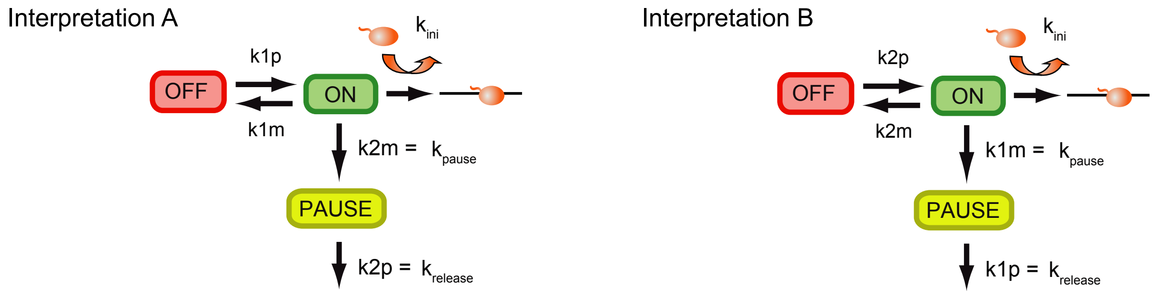

Figure S4

Supplementary Figure S4. Bursting characteristics of the latently infected HeLa cell clones.

A- Fluctuations of HIV-1 transcription over 8 hours, with one image stack recorded every 3 minutes, for the three latently infected HeLa clones. The x-axis represents the time (in hours) and y-axis represents the intensity of a transcription site, expressed in arbitrary units. Periods of HIV-1 promoter activity are colored in green.

B- Analysis of the active periods of the HIV-1 promoter, for the three latently infected HeLa clones. The graphs are histograms with the duration of active (ON) periods on the x axis, and the number of events in the y axis. The pie-charts represent the total duration of OFF periods in red, and of ON periods in green. Light red and light green represent incomplete events, initiated before the movie started or interrupted by the movie end.

C- Fits of the experimental survival function for the clone 12. The graph represents the survival functions reconstructed from the live cell data for the clone 12, with the part deriving from the short and long movies in red and green, respectively. Blue line: fit of the 3-state model with a facultative pause; "+": fit of the 3-state model with an obligatory pause; "x": fit with a facultative pause. x-axis: time intervals between successive initiations events, in seconds and in $\log_{10}$ scale. y-axis: probability of $\Delta t > x$ ($\log_{10}$ scale).

D- Expression of Tat in latently infected HeLa cells. Panels are microscopy images of the indicated cell line where Tat is detected by immunofluorescence with an anti-FLAG antibody (the version of Tat used carries a FLAG tag at its C-terminus). Blue: Dapi; red: smFISH signal; scale bar:15 microns. This experiment was performed twice with similar results.

E- Fitted parameters of the facultative pausing model for the indicated cell lines. The graphs at the bottom depict the two possible interpretation of the model parameters.

Supplemental Notes : Deconvolution Method for Reverse Engineering of Transcriptional Bursting Models

1 Introduction

1.1 Summary of the method

We use machine learning to derive characteristics of single cell transcription activity from MS2 data. The output of the machine learning procedure is threefold. Using a **deconvolution method** and high resolution movies, we generate a **time map of transcription events** indicating, for each cell, the moments when different RNAP molecules start producing mRNA. This direct readout of transcriptional events in a cell population, represents a unique feature of our method, not available in other methods that fit directly a particular transcription model to the MS2 data such as methods based on the autocorrelation function [2, 5, 4], or maximal likelihood estimate [1] or on Bayesian inference [9, 8]. The map can be used for direct characterisation of transcription features, such as polymerase convoys and various statistics of inter-event times. A second output of the approach is a **multiscale cumulative distribution function** of the waiting time separating succesive transcription events (or the complementary function, called survival function). We provide both non-parametric Kaplan-Meyer and parametric multi-exponential estimates of the multiscale distribution function. This distribution, obtained by combining short, high-resolution and long, low-resolution movies, covers timescales from second to 10 hours. The dynamical range of our method supersedes those of other extant methods that are based on much smaller sampling rates and/or much shorter movie lengths. The waiting time distribution is model-free, but can be further used to identify various models of transcription dynamics. The third output of our method is the **model parameter identification**, simultaneously for several transcription models that fit the data equally well. Although we focus on discrete transcription models based on Markovian transitions among hidden promoter states (for different number of states and for a rich collection of transition graph topologies), our method can be extended to the identification of more general models, including continuous or hybrid ones [6]. Contrary to

other methods that need separate fitting procedures for different models, in our method a single parametric fit of the multiscale waiting time distribution function is enough for identifying simultaneously a large collection of models that are all compatible with data and perform equally well. Another novelty with respect to other model fitting methods is the use of exact symbolic solutions, relating the parameters of the multiscale distributions to kinetic parameters of the model. For several models there is one-to-one relation between parameters of the distribution and kinetic parameters of the model. In this situation, the model kinetic parameters can be obtained analytically from the parameters of the multiscale distribution. Our method also leads to uncertainty estimates of the model parameters, based on optimal and close-to-optimal parametric fits of the multiscale distributions. As a matter of fact, models that are structurally different (different numbers of promoter states, or different transitions between states) but fit equally well, can differ in their parametric uncertainties. Therefore, parametric uncertainty can be used as a model selection criterion that favor precise and reject uncertain models. The symbolic part of our method also identifies situations when parametric uncertainty results from degeneracy, more precisely when there are manifolds of parameters that lead all to exactly the same goodness of fit. This situation arises when the relation between parameters of the multiscale distribution and the model parameters is one to many. Model and/or parameter uncertainty can be ultimately lifted by direct measurements of one or several kinetic parameters.

1.2 Discrete Markovian models for transcription dynamics

A Markovian model of transcription dynamics includes stochastic transitions between several ON and OFF promoter states (Figure S5). Rather generally there is a ON state and several OFF states. The promoter transcribes only in the state ON when it can trigger several departures of RNAP molecules along DNA. The departure of one RNAP is when the model reaches the state EL. It is considered that immediately after departure the operator site becomes free (the transition from EL to ON is instantenous). The transitions define a continuous time Markov chain characterized by a set of positive parameters k_{ij} representing the transition probability per unit time (or equivalently the inverse mean transition time) from state i to state j. Given the number of states N, the structure of the Markov chain is defined by the directed graph $G = \{(i,j)|1 \leq i \leq N, 1 \leq j \leq N, k_{i,j} \neq 0\}$; several possible structures with $N = 3$ are shown in Figure S5. We show in the sequel how the parameters k_{ij} of a model can be adjusted to reproduce the transcriptional bursting and RNA synthesis observed in the live cells experiments. The parameter estimates are performed simultaneously for several possible model structures.

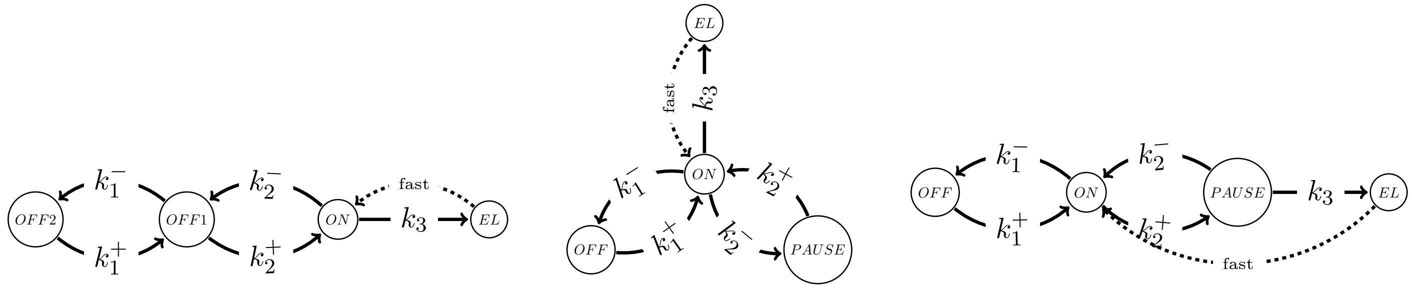

a) M1 (no pause, two non-productive states)

b) M2 (eventual pause, systematic abortion)

c) M3 (systematic pause, eventual abortion)

Figure S5: Three state, three exponential models of transcription dynamics. Transcription dynamics is represented as transitions between two OFF and one ON promoter states. ON represents the productive state. EL represents the elongation state which immediately liberates the promoter (from EL there is always fast return to ON). ON may not lead systematically to EL, for instance if there is transcription pausing. In these models, the pausing state is represented as one of the OFF state. If the pause leads systematically to transcription abortion the pause state leads to ON (model M2); otherwise it leads both to ON and to EL, with different probabilities (model M3). In model M1 the inactive state OFF1 can lead to another inactive state OFF2. The represented topologies differ by the connections between different states. The constants k_i are inverses of transition times, as such a) M1: k_3 is the initiation rate; b) M2: k_3 is the initiation rate, k_2^+ is the abortion rate, k_2^- is the pause enter rate; c) M3: k_3 is the pause exit rate and k_2^- is the abortion rate. All the represented models have 3 states (excepting the final elongation state), 5 kinetic parameters and their stochastic transcription activity can be described by a three exponential survival function.

1.3 The machine learning procedure

This procedure was initially designed for MS2 data obtained from human cell cultures but it has also been applied to in vivo study of Drosophila embryo development [3].

The machine learning procedure has several steps:

a) The first step is the **numerical deconvolution of the signal and Pol II Positioning**. The signal from each cell is a convolution between the contribution of a single polymerase and the point process (set of time points) describing all the start transcription events. For each cell, we reconstruct the start events by a least square optimization method performed by a genetic algorithm. We prefer optimization to Fourier

transform based deconvolution in order to avoid the Gibbs phenomenon (the signal produced by a single polymerase is discontinuous).

b) The second step is the **non-parametric estimate of the survival function**. The data resulting at a) (positions of transcription start events) is used to estimate the survival function which is the complementary cumulative distribution function of the inter-event (waiting) times. Further complexity is brought in at this step by the utilization of two types of movies with short and long time resolution. Only short movies data undergoe deconvolution, the long movies are used to obtain long waiting times directly. This results into two distribution functions that are joined together (by affine transformations corresponding to the law of total probability) to cover many decades of timescales (second to ten hours). In certain applications, the hours scale is not reachable because of biological constraints. For instance, in developmental biology, the studied developmental stage may be too short. In this case, we use only short movies and the joining step is not needed.

c) The third step is the **multi-exponential regression of the survival function**, performed by gradient optimization with random starting guesses, uniformly distributed in logarithmic scale (this choice is dictated by the multiscale nature of the signal). Usually, two or three exponentials (i.e. two or three time scales) are enough to describe our data. Chosing more than three exponentials is justified when this improves the fit without increasing parameter uncertainty. Conversely, choosing less exponentials is justified if this reduces uncertainty without diminishing the fit too much. The first three steps of our procedure are model-free because they make no assumption about the mechanism of the transcription regulation.

d) The last step of the procedure is the **symbolic reverse engineering of transcription models** from the survival function. We consider that the transcription machinery has several discrete states among which only one is productive (state ON in Figure S5). Then, the waiting time between successive transcription start events is the first return time to the productive state ON. The distribution of this waiting time satisfies a system of ODEs whose solution can be expressed as a sum of exponentials. The inverse problem consists in computing model's kinetic parameters from the parameters of the multi-exponential regression. We have developed a symbolic solution to perform this step. Our symbolic solution also tackles the ill-posed character of the inverse problem. Indeed, although the same distribution function can be produced by several models with different structures, the significance and the value of each parameter are different in different models. Moreover, we know precisely how to pass from one model to another by changing the

parameter values. It is therefore enough to perform a direct independent experimental measurement of a single parameter in order to discriminate between different models. In the case of redundant parameters (parameters not influencing independently the observed distribution function) and parameter uncertainty, some parameters may remain independent and can be used for model discrimination.

2 Numerical deconvolution of short movies

2.1 Description of the problem

The experimental data obtained from short movies is shown in the Figure S6 for the HIV-1 promoter. The signal intensity from the mRNA MS2 reporter is represented as a function of time for each active transcription site. We are interested in reconstructing from this signal the sequence of waiting times between successive transcription start events (see Figure S7), for each transcription site.

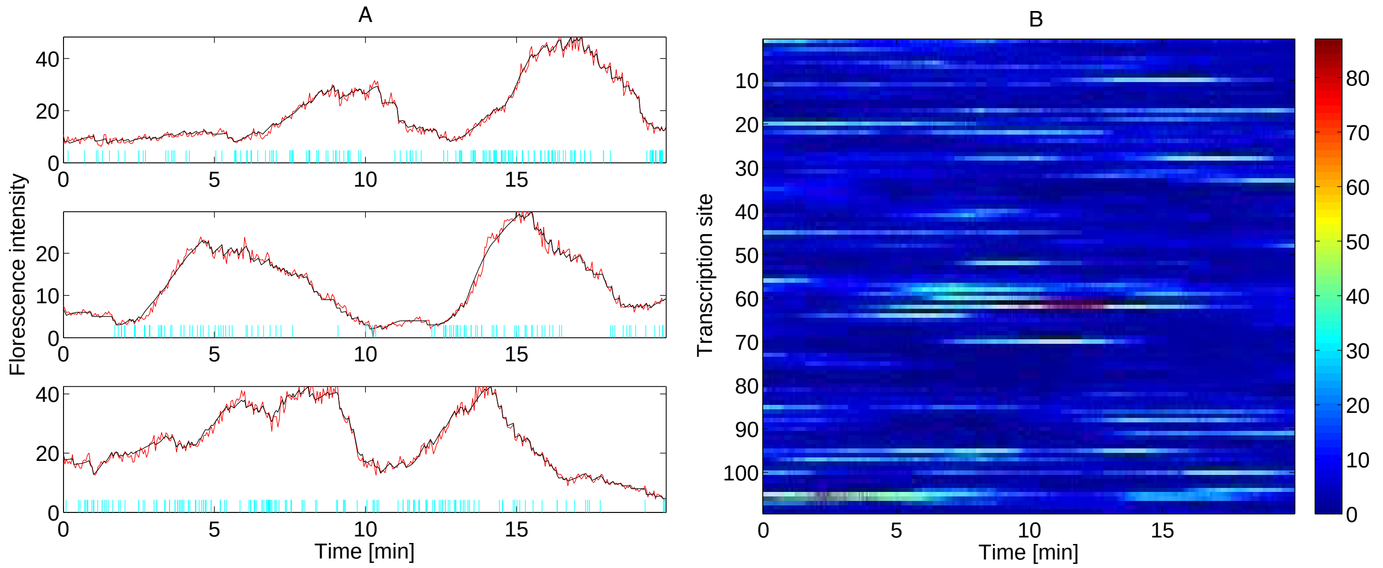

Figure S6: Short movie data for a HIV-1 promoter (no tat condition). A) fluorescence intensity vs. time for several transcription sites (red line); the reconstructed polymerase positions and signal are indicated as vertical cyan bars and black line, respectively. B) colormap of intensity for all transcription sites in a short movie.

Transcription events can not be straightforwardly detected from local features of the intensity signal because at a given time and for the same transcription site, more than one polymerase transcribe simultaneously. Furthermore, the signal from one polymerase does not appear immediately after initiation (see below).

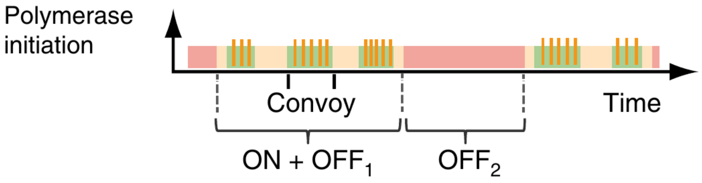

Figure S7: Dynamics of the promoter changing states. Start events are represented as red bars. During a ON period several polymerases start, forming a convoy. For this signal, two types of non-productive states, short (OFF1) and long (OFF2) can be obseved.

One should thus consider that experimental data is a a convolution between the sequence of start events $\{t_i, 1 \leq i \leq N_{pol}\}$ and the signal $h(t)$ from a polymerase molecule:

$$S(t) = \sum_{i=1}^{N_{pol}} h(t - t_i), \tag{1}$$

where N_{pol} is the number of polymerases contributing to the signal. N_{pol} is not known and will be determined by the optimization procedure (see below). The parameters t_i are the initial polymerase positions on the DNA, indicating the transcription start events.

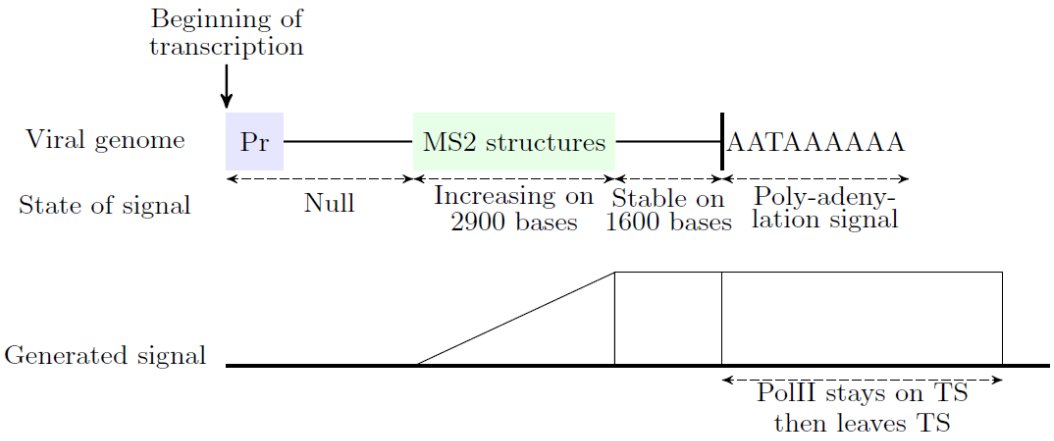

Figure S8: Representation of the signal from one polymerase for the HIV1-promoter. The parameters are indicative and can change for other applications.

The polymerase signal $h(t)$ can be described as follows (see Figure S8):

i) Transcription begins when the RNA polymerase II leaves the promoter. However, no signal will be generated

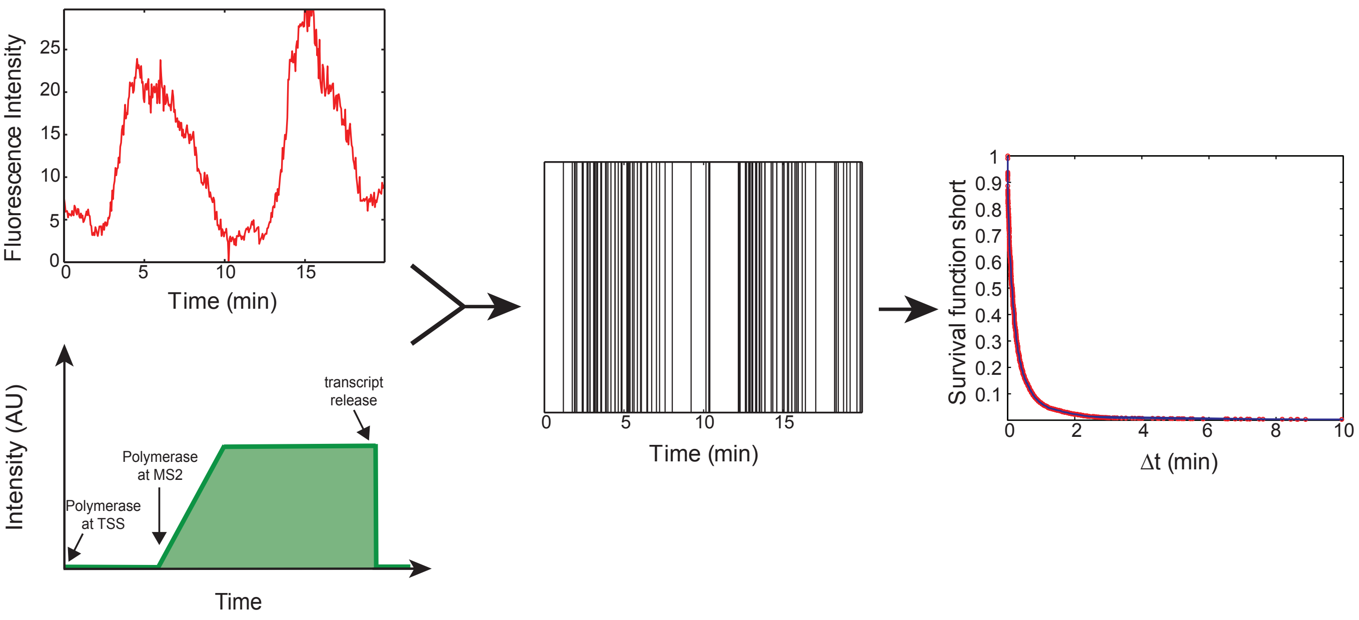

Figure S9: Distribution of transcription initiation events can be reconstructed by deconvolution.

yet.

ii) The fluorescence signal is generated as soon as the polymerase reaches the MS2 sequence. During the transcription of the MS2 sequence the signal can be represented as a linear ramp-up.

iii) The signal will stay constant from the end of the MS2 sequence until when the polymerase leaves the transcription site, when the signal falls abruptly.

In order to compute the times corresponding to the three stages we use the length (expressed in base pairs) of the three sequences PRE, SEQ and POST (before MS2, MS2 and post MS2). These lengths depend on the MS2 construction (the values in our HIV-1 experiments are PRE=700bp , SEQ=2900bp, POST=1600bp). The sequence lengths are divided by the polymerase speed V_{pol} to be transformed into times. For our HIV-1 promoter we have used $V_{pol} = 67bp/s$ (see main text). An extra time $P_{poly} = 100s$ is added to POST, corresponding to the polyadenylation signal (during this time the polymerase has finished transcription and waits on the transcription site).

After signal calibration the unit of fluorescence represents the amplitude of the signal from one polymerase. Using this model we want to reconstruct the sequence of initiation events by deconvolution (see Figure S9).

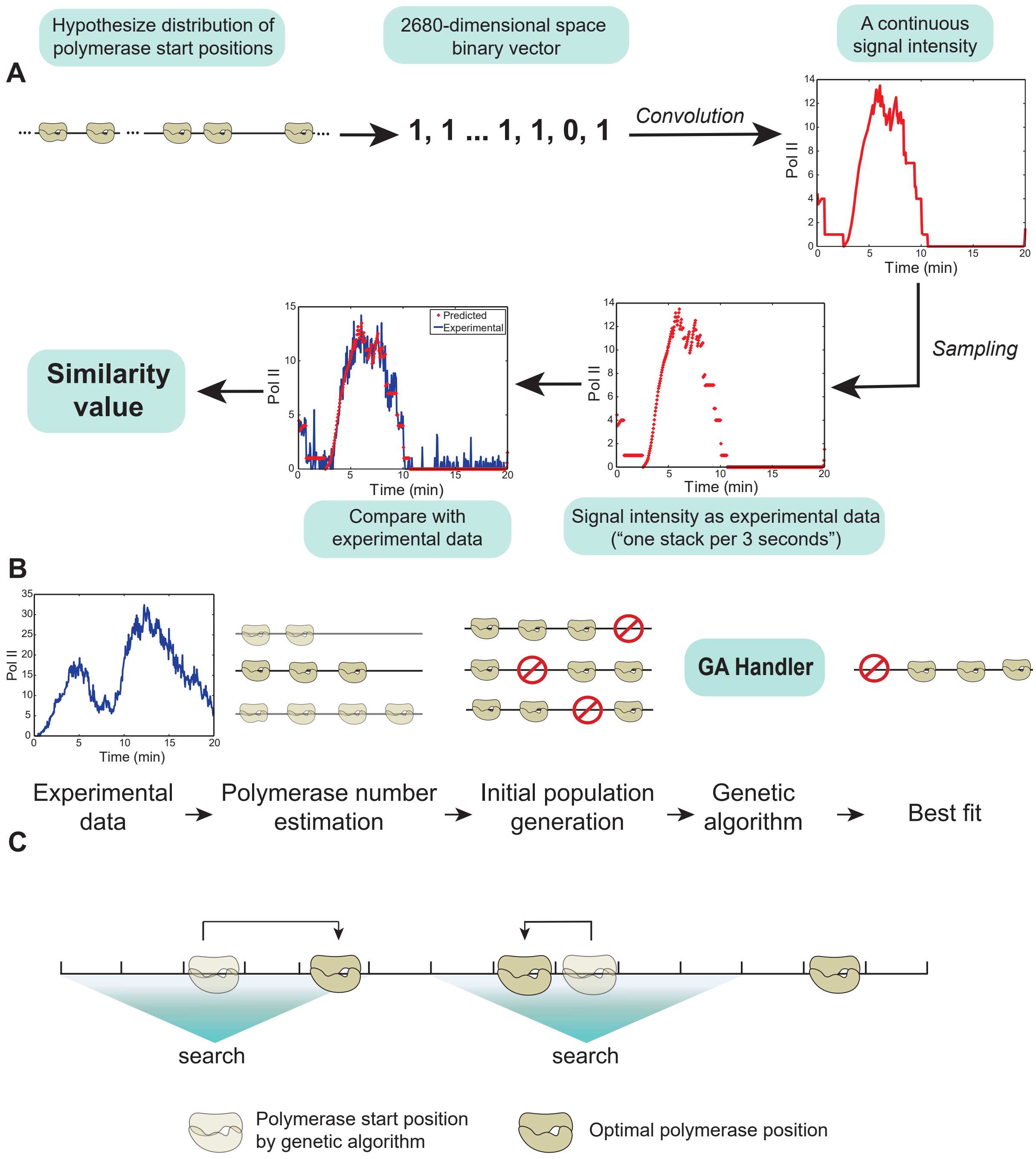

Figure S10: A. Flowchart of the numerical deconvolution method. B. Genetic algorithm step. C. Local optimization step.

More precisely, we will determine N_{pol} and $t_i, 1 \le i \le N_{pol}$ that minimize the following objective function:

$$\mathcal{O}_1 = \sum_{k=1}^{N_{exp}} [S(k\delta) - S_{exp}(k\delta)]^2, \quad (2)$$

where δ is the time step (inverse frame rate), N_{exp} is the number of frames, S is described by (1) and S_{exp} is the experimental signal. For a short movie, the frame rate is $1/3s^{-1}$, thus $\delta = 3s$ and $N_{exp} = 400$ for a movie length of $T_{max} = 20$min.

2.2 Discretization of the optimization problem

It is useful to use a dual representation of the polymerase positions t_i in terms of seconds and base pairs on the DNA sequence. Although the polymerase positions are in principle continuous variables, for computation reasons we discretize them. In this dual representation, it is natural to consider that possible polymerase positions are multiples of the minimum distance d_{min} between two polymerases ($d_{min} = 30$bp in our program). The precise value of d_{min} is not needed. Generally, d_{min} should be chosen as small as possible to guarantee precision of the polymerase positions. It should be smaller than the real minimum distance between polymerases and larger than a value dictated by the computation costs (the computation costs increase when d_{min} decreases).

Using this discretization, polymerase positions are coded as a binary vector. Every possible polymerase position will have either 1 or 0 value, which represents if there is a polymerase in the current position or not. Considering that the polymerase speed is constant, the polymerase positions are all we need to determine the signal. For a movie of length $T_{max} = 20$min and for $V_{poly} = 67$bp/s, the polymerase positions are represented as a binary vector of length $N = T_{max}V_{poly}/d_{min} = 2680$. Considering discretization steps larger than d_{min} is also possible. In this case binary vectors are shorter and computation is faster, however the precision may be reduced.

2.3 Solve the deconvolution problem by a genetic algorithm followed by local optimization

Every binary vector of dimension N represent the polymerase start positions and determine a value of the objective function (2). The opposite of the objective function is the *fitness*. The deconvolution problem represents finding the minimum of this objective function (maximum of fitness) in the N dimensional binary space (Figure S10). In order to solve this hard combinatorial problem, we apply first a global optimization genetic algorithm (GA).

As shown in Figure S10 B, GA follows three steps: estimating the amount of polymerases, generating an initial population and applying genetic algorithm. We estimate the number of polymerases N_{pol} from the signal integral intensity, as the ratio of integral intensities of the experimental signal and of the single polymerase signal. The resulting amount is not an accurate number, and it is a rough estimation which can be used to accelerate next steps. Then we prepare an initial population according to the estimation of polymerase amount. Starting with a vector with N '0's, we randomly pick N_{pol} positions and change them into '1's. After the preparation of initial population, we use the genetic algorithm implemented in the GA solver provided by Matlab global optimization toolbox. Mutation, crossover and selection are processed by the MATLAB built-in function ga (MATLAB, version (R2013b), Natick, Massachusetts: The MathWorks Inc.). At each step, the genetic algorithm solver selects individuals at random from the current population to be parents and uses them to produce the children for the next generation. Over successive generations, the population "evolves" toward an optimal solution.

In order to verify this method, we implemented a test using an artificial experimental signal. We deconvolved the artificial signal, for which we know exactly the polymerase start positions. The simulation of genetic algorithm, as in the example of Figure S11, shows that the genetic algorithm can approximately reconstruct the signal. However, the global minimum is not precisely reached and the polymerase start positions of simulation are not exactly the same as the artificial ones (Figure S11).

There are various reasons why polymerases were not exactly placed into right positions as follows: the limitation of the maximum number of iterations, the limitation of population size, the initial error in the estimated number of polymerases, the noise generated by the algorithm, etc.. Although GA can not give a precise result (or it is time consuming to get a precise result), it provides a solution not far from the optimal result.

With this in mind, we use a local exhaustive search to accomplish local optimization. The idea is to "move" a polymerase left or right relative to the GA found position to see if this improves the fitness function (Figure S10 C). For every polymerase we find the best position which has the highest fitness value and we update the best positions for all of them. The local optimization result is shown in Figure S11. By this method, practically all the polymerases were arranged into the correct positions. The local optimisation method has limitations, for instance it does not allow correction of the total number of polymerases; we suppose that this number has already been found by the GA.

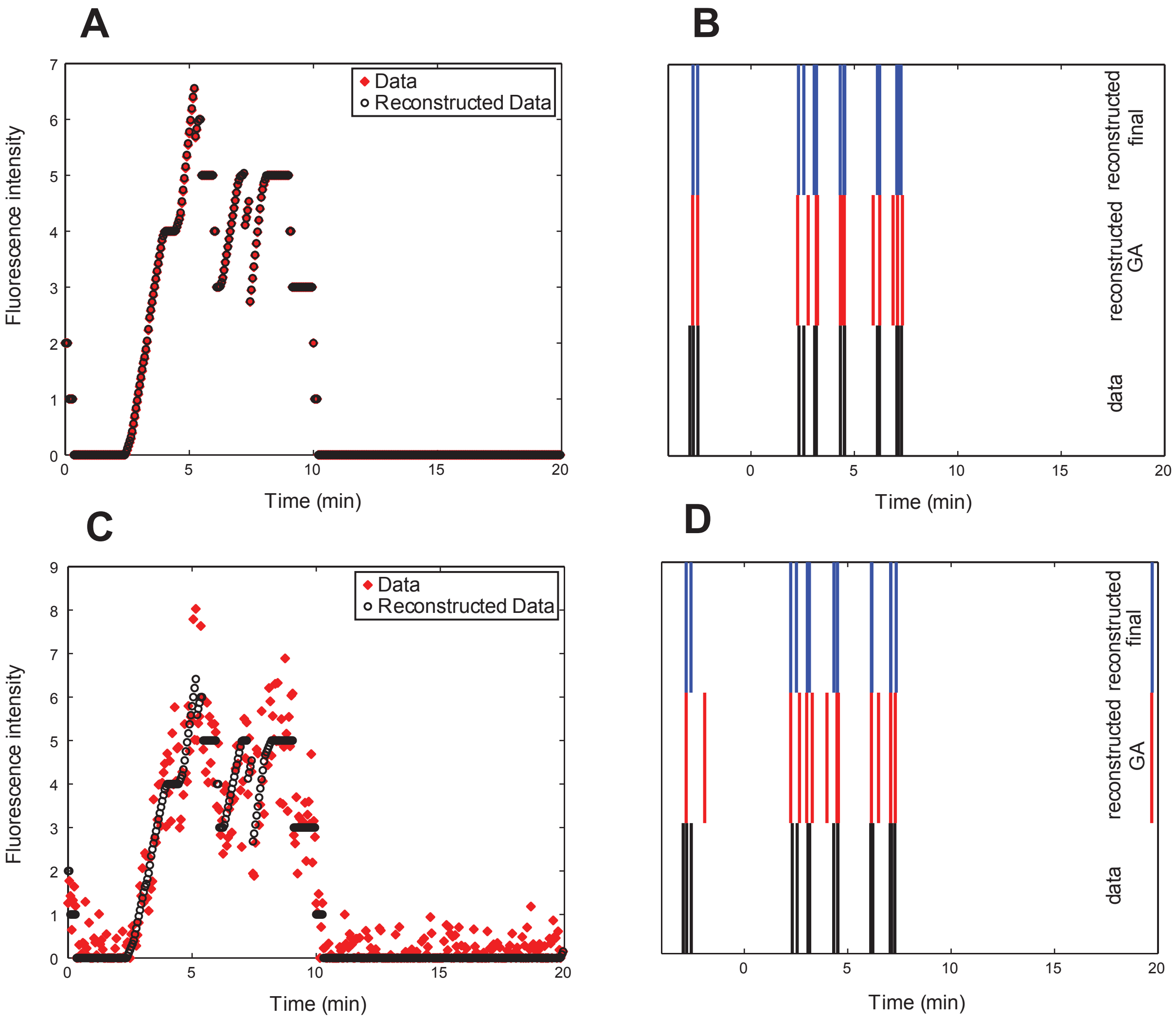

Figure S11: Result of the deconvolution step on artifical data. A) Signal generated artificially (no noise). B) Original polymerase positions compared to positions resulting from the genetic algorithm step and to the positions corrected by local optimisation (no noise). C) Signal generated artificially (noise added according to the procedure described in Section 8). D) Original polymerase positions compared to positions resulting from the genetic algorithm step and to the positions corrected by local optimisation (noise added).

3 Multi-exponential regression of the distribution function

From the numerical deconvolution step, we obtain the series of polymerase start positions, for each active transcription site detected in the short movies.

From each transcription site we compute waiting times defined as time interval between successive positions.

When the position is the last one in the movie, the waiting time is defined as the distance to the end of the movie. Considering that all transcription sites are statistically equivalent, we gather the waiting times from all sites that are active in the same short movie.

Long movies also provide waiting times by a different method, without numerical deconvolution (see below).

We consider that the transcription events form a renewal process with independent, identically distributed waiting times Δ. This property is valid at stationarity, but not only. For instance, in Markovian models, the property is true if after every transcription event the system allways returns to the same state. Given that non-Markovian models can be made Markovian by adding hidden states, we believe that the property is quite general. All the models from Figure S5 satisfy this property, because from the EL state one can only go to the ON state.

We want to estimate the *complementary cumulative distribution function* (also called *survival function* in survival analysis) of the waiting times defined as:

$$S(t) = \mathbb{P}[\Delta > t]\,. \tag{3}$$

3.1 Waiting times from short movies: Δ_s

Outliers handling

Several observed transcription sites had abnormal behaviour (too many or too few events). The decision was made to take them off the data set as follows

1. Compute the amount of events of transcription that happened during the movie (Ev).

2. Compute the 1st and the 3rd quartile (respectively Q1 and Q3) of the distribution of Ev .

3. Only consider the transcription sites where

$$Q1 - 2.5(Q3 - Q1) < Ev < Q3 + 2.5(Q3 - Q1)$$

Affine transformation parameter p_s, the probability that the waiting time is shorter than the short movie length.

The Δ_s are the waiting times deduced from the short movies, therefore they all satisfy the condition $\Delta_s < T_{max}$, where T_{max} is the movie length. Therefore, this data does not reconstruct the full survival function, but the conditional survival function $S_{<T_{max}}(t) = \mathbb{P}[\Delta > t | \Delta < T_{max}]$.

In order to compute the relation between $S(t)$ and $S_{<T_{max}}(t)$ we use the total probability theorem:

$$\mathbb{P}[\Delta > t] = \mathbb{P}[\Delta > t|\Delta < T_{max}]\,(1 - \mathbb{P}[\Delta > T_{max}]) + \mathbb{P}[\Delta > t|\Delta > T_{max}]\,\mathbb{P}[\Delta > T_{max}]\,. \tag{4}$$

Let us note that for $t < T_{max}$, one has $\mathbb{P}[\Delta > t|\Delta > T_{max}] = 1$. Hence, from (4) it follows that

$$S_s(t) = (1 - p_s)S_{<T_{max}}(t) + p_s, \text{for } t < T_{max}, \tag{5}$$

where $p_s = \mathbb{P}[\Delta > T_{max}]$ is the probability that the waiting time is longer than the length of the short movie.

In other words, for short movies, the survival function is obtained from the conditional survival function by an affine transformation.

3.2 Waiting times from long movies: Δ_l

Active and inactive periods, threshold parameter

The frame rate for long movies is $1/3min^{-1}$ and the typical length is $9h$. Let us notice that the deconvolution procedure is not possible for long movies, because the number of polymerases is too large. Therefore, long waiting times are obtained directly from the signal. For long movies, there is no need to calibrate the fluorescence intensity, nor to deconvolve the signal. An intensity threshold is defined and a given transcription site is considered active in a given frame if its intensity is larger than the threshold, inactive if not, see Figure S12.

Outliers handling

We define the fraction of inactivity (FI) as the ratio of cumulative total inactivity time to the total cumulative time in the long movie and for all the transcription sites.

Some transcription sites in long movies data set also show unusual behaviours being active (FI=0) or inactive (FI=1) during the entire movie. We exclude these outliers as we did it for the short movies, but based on the fraction of inactivity for each transcription site.

We will only consider the transcription sites from the long movies where

$$Q1 - 2.5(Q3 - Q1) < FI < Q3 + 2.5(Q3 - Q1)$$

Corrected waiting times, parameter Δ_0

In the long movies the waiting times Δ_l between successive transcription initiations correspond roughly to the inactive periods Δ_I. As a matter of fact, these waiting times can be longer than the inactive periods by a time varying between 0 and 6min (because the signal needs about 3min to vanish and starts about 3min before

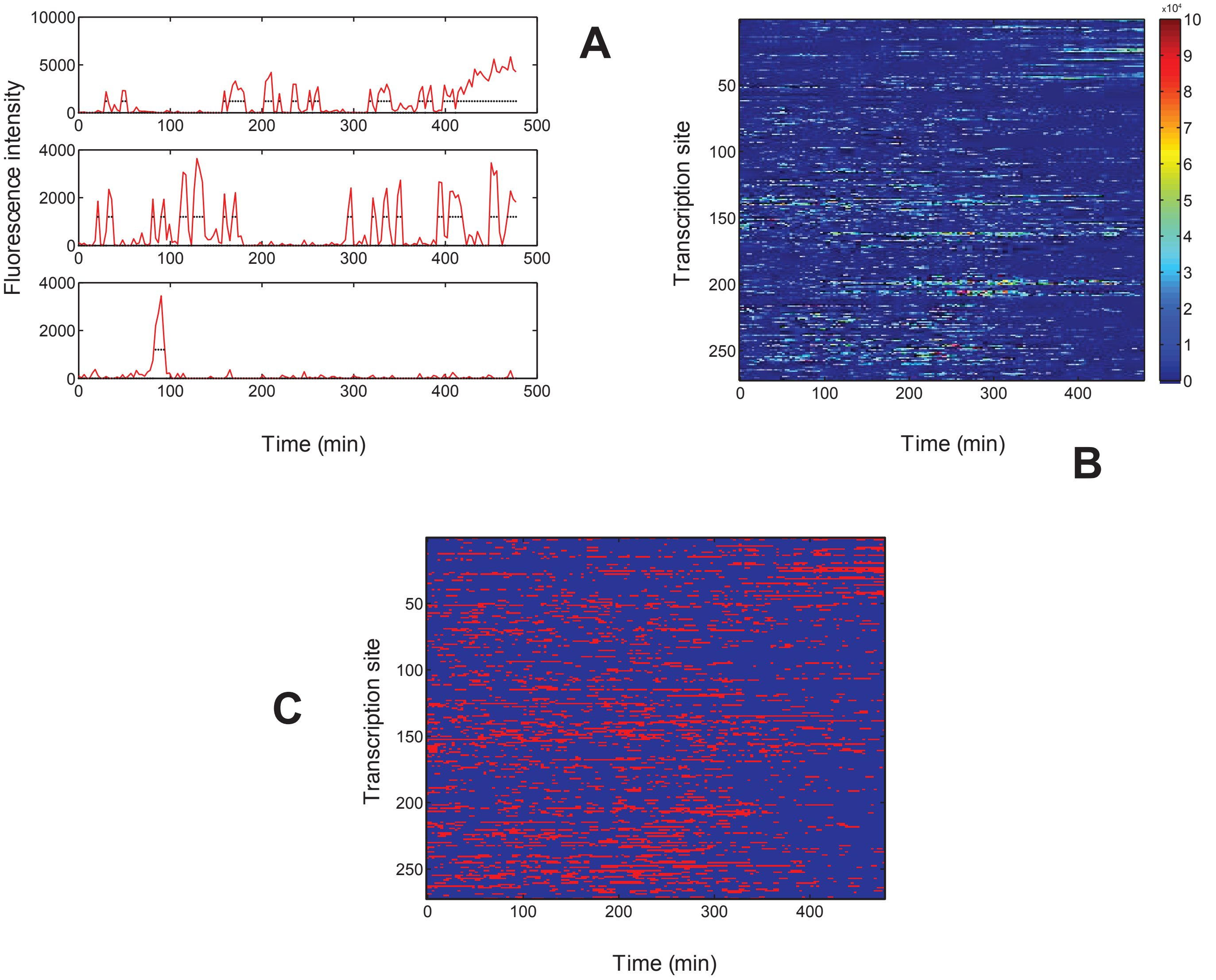

Figure S12: Long movie data for a HIV-1 promoter (no tat condition). A) intensity of fluorescence vs. time for several transcription sites; the real valued intensity are transformed into a binary signal (dots) by thresholding (here the threshold value is 1200). B) intensities for all transcription sites. C) Binary valued intensities for all transcription sites.

it is detected). This unknown time is a parameter of the method and its values are discretized to $\Delta_0 = 0, 3, 6$. All waiting times are computed as $\Delta_l = \Delta_I + \Delta_0$.

Affine transformation parameter p_l, the probability that the waiting time is larger than the long movie resolution time.

The signal from a single polymerase lasts roughly 3min (see Figure S8). In this case, waiting times shorter than T_{min} where T_{min} is the time resolution of the long movie (roughly 3min) can not be observed. The observed conditional survival function is now $S_{>T_{min}}(t) = \mathbb{P}[\Delta > t | \Delta > T_{min}]$.

We can note that for $t > T_{min}$, one has $\mathbb{P}[\Delta < t | \Delta < T_{min}] = 1$.

Once again, from the total probability theorem (4), it follows:

$$S_l(t) = p_l S_{>T_{min}}(t), \text{for } t > T_{min}, \tag{6}$$

where $p_l = \mathbb{P}[\Delta > T_{min}]$ is the probability that the waiting time is longer than T_{min}, S_l is the survival function for long movies.

Estimate of parameter p_l

The probability p_l is estimated by combining information extracted from the long and the short movies. p_l is precisely the probability that waiting times are observed as inactive periods in the long movie. Let $N_{inactive}$ and N_{active} be the number of waiting times observed as inactive periods, and hidden within active periods of the long movie, respectively. $N_{inactive}$ can be determined directly from the long movie, it represents the number of observed inactive periods. N_{active} is obtained as the ratio $P_{active}/\mathbb{E}[\Delta | \Delta < T_{min}]$ where P_{active} is the cumulative time of all active periods in the long movie and $\mathbb{E}[\Delta | \Delta < T_{min}]$ is the conditional expectancy of the waiting time provided that this is smaller than T_{min} therefore undetectable by the long movie. By definition one has

$$\mathbb{E}[\Delta | \Delta < T_{min}] = \frac{\int_0^{T_{min}} u f(u)\, du}{\mathbb{P}[\Delta < T_{min}]},$$

where f is the probability density function of Δ. Taking the derivative of $S(t) = \mathbb{P}[\Delta > t] = \int_t^\infty f(u)\, du$ we get $f(t) = -S'(t)$. Using the integral by parts formula we find

$$\mathbb{E}[\Delta | \Delta < T_{min}] = \frac{-T_{min} S(T_{min}) + \int_0^{T_{min}} S(u)\, du}{1 - S(T_{min})}.$$

Summarizing, we find

$$p_l = \frac{N_{inactive}}{N_{inactive} + N_{active}} = \frac{N_{inactive}}{N_{inactive} + \frac{P_{active}(1 - S(T_{min}))}{-T_{min} S(T_{min}) + \int_0^{T_{min}} S(u)\, du}}. \tag{7}$$

Both $S(t)$ and the integral above are computed using the survival function of the short movie. $N_{inactive}$ and P_{active} are determined from the long movie data.

Estimate of parameter p_s

p_s is estimated by optimization. We look for the value of p_s that minimizes the square distance between the solutions (5) and (6) on the overlap interval $[T_{min}, T_{max}]$.

The survival functions after calculation of p_l, p_s and affine transformations are shown in Figure S13.

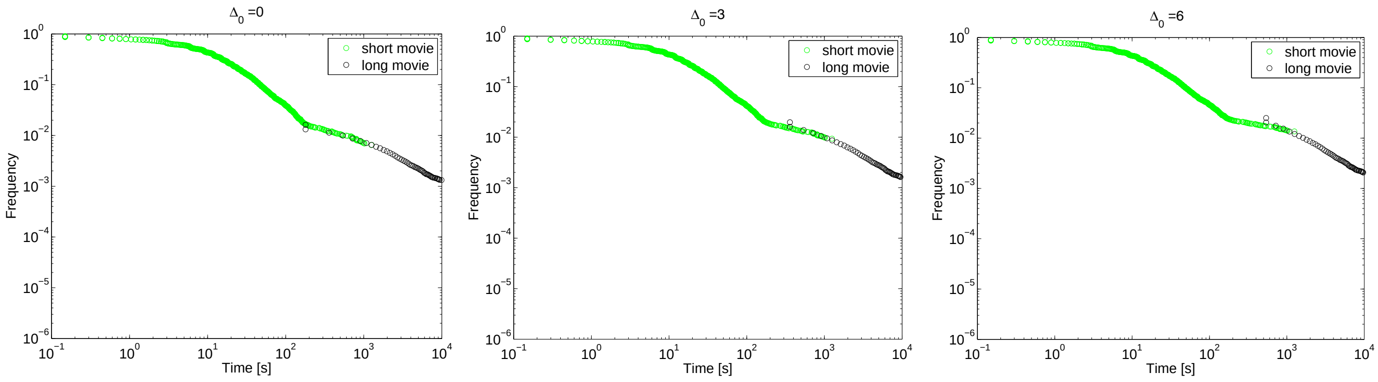

Figure S13: Survival functions of the waiting time after affine transformations for several values of the shift Δ_0 (HIV promoter, no tat condition, see text).

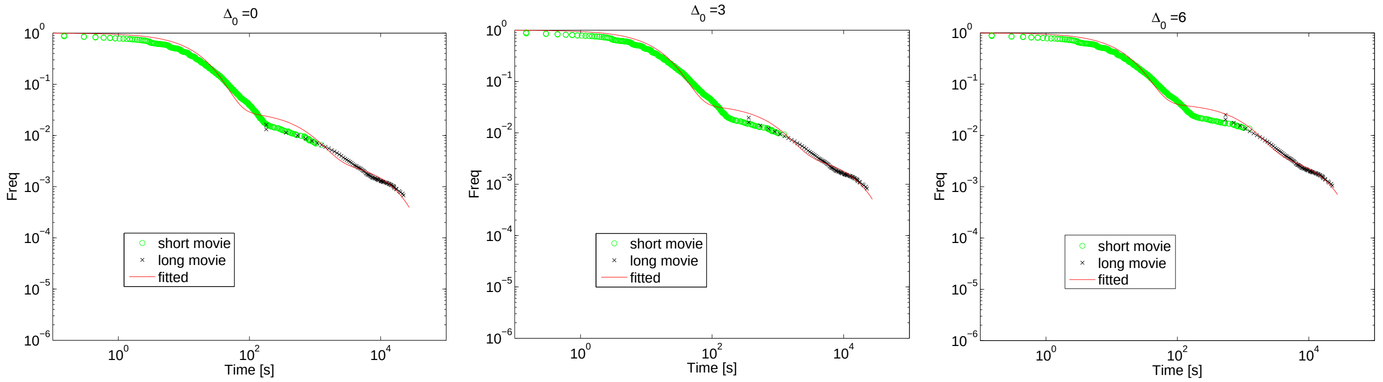

Figure S14: Multi-exponential regression ($N = 3$) for several values of the shift Δ_0 (HIV promoter, no tat condition, see text).

3.3 Multiexponential regression

The previously determined survival function function (5), (6) is modelled by a multiexponential function:

$$S(t) = A_1 \exp(\lambda_1 t) + A_2 \exp(\lambda_2 t) + \ldots + (1 - A_1 - A_2 - \ldots - A_{n-1}) \exp(\lambda_n t), \tag{8}$$

where $A_1, \ldots, A_{n-1}$, $\lambda_1, \ldots, \lambda_n$ are $2n - 1$ parameters.

Because $\lim_{t\to\infty} S(t) = 0$, these parameters must satisfy the constraints $\lambda_i < 0, 1 \leq i \leq n$. For practical reasons we can consider that all λ_i are distinct. Degenerate cases, when two or more λ_i are equal can be uniformly approximated by formula (8) with distinct λ_i (see the section 4.2). Up to relabelling we can consider that $|\lambda_1| > |\lambda_2| > \ldots |\lambda_n|$. Furthermore, because the complementary distribution function is always decreasing we have

$$S'(t) < 0, \forall t \geq 0. \tag{9}$$

The condition (76) implies that $\sum_{i=1}^{n} A_i\lambda_i < 0$, where $A_n = 1 - \sum_{i=1}^{n} A_i$ (follows from $S'(0) < 0$) and that $A_n > 0$ (this follows from $\lim_{t\to\infty} S'(t)exp(-\lambda_n t) = A_n\lambda_n < 0$ and $\lambda_n < 0$). The hyperplanes $\sum_{i=1}^{n} A_i\lambda_i = 0$, $A_n = 0$ together with other manifolds delineate the domain of valid parameters A_i. This domain depends on the exponents λ_i as illustrated for $n = 3$ in Figure S15.

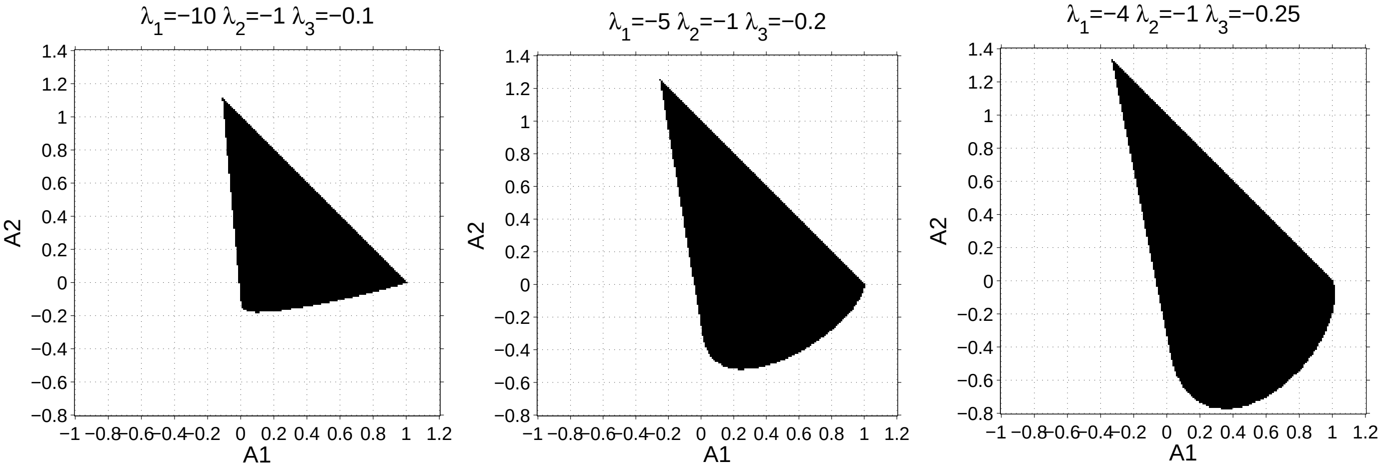

Figure S15: Permitted values of $A_i, 1 \leq i \leq n$ for $n = 3$ are represented in black for various λ_i. These parameters values are defined by the condition $S'(t) = \sum_{i=1}^{3} \lambda_i A_i \exp(\lambda_i t) < 0$, $\forall t \geq 0$, where $A_3 = 1 - A_1 - A_2$. The permitted values are limited at the top right by the line $A_1 + A_2 = 1$ and at the left by the line $\lambda_1 A_1 + \lambda_2 A_2 + \lambda_3(1 - A_1 - A_2) = 0$.

Like usually in machine learning, the choice of n is guided by a parcimony principle. One can start with $n = 2$ and progressively increase n until the goodness of fit stops improving (at equal goodness of fit, one favors the model with lowest complexity, lowest n and/or with lowest parameter uncertainty).

The objective function is defined as follows:

$$\mathcal{O}_2 = \frac{\alpha}{n_s}\sum_{i=1}^{n_s}(S(t_i^s) - S_s(t_i^s))^2 + \frac{\alpha}{n_l}\sum_{i=1}^{n_l}(S(t_i^l) - S_l(t_i^l))^2 + \frac{1-\alpha}{n_s}\sum_{i=1}^{n_s}(\log(S(t_i^s)) - \log(S_s(t_i^s)))^2 + \\ + \frac{1-\alpha}{n_l}\sum_{i=1}^{n_l}(\log(S(t_i^l)) - \log(S_l(t_i^l)))^2, \qquad (10)$$

where $S(t)$ is defined by (8); S_s and S_l are computed by (5),(6), respectively; t_i^s, t_i^l are sampling times for short and long movies, respectively; α is a positive weight representing the relative importance of the linear scale compared to the logarithmic scale in the representation of the survival function.

We minimize (10) by local optimization (Levenberg-Marquardt algorithm implemented in the Matlab function *lsqnonlin*) starting with N_p (in our program $N_p = 100$) random values of the regression parameters $A_1, \ldots, A_{n-1}$, $\lambda_1, \ldots, \lambda_n$. The initial parameters $A_1, \ldots, A_{n-1}$ are chosen uniformly distributed in the cube $[-M, M]^{n-1}$ (we used $M = 2$), whereas the initial parameters $\lambda_1, \ldots, \lambda_n$ are all negative and log-uniformly distributed in absolute value. More precisely, $\log(|\lambda_i|)$ are uniform in a cube $(l_1, l_2, \ldots, l_n) + [-K, K]^n$, where $l_1 < l_2 < \ldots < l_n$.

The optimization is repeated for all values of Δ_0 and each time repeated N_p times with different initial parameters (Figure8). We keep the lowest value $\mathcal{O}_2^{min}$ of (10) as well as sub-optimal solutions with $\mathcal{O}_2 < 1.5\mathcal{O}_2^{min}$. The suboptimal parameters are utilized to estimate the parameter uncertainty. For each parameter we compute an uncertainty interval defined by the minimum and the maximum values over the set of all optimal and suboptimal parameters. Uncertain parameters have large uncertainty intervals.

An example of multi-exponential fit is given in Figure S16.

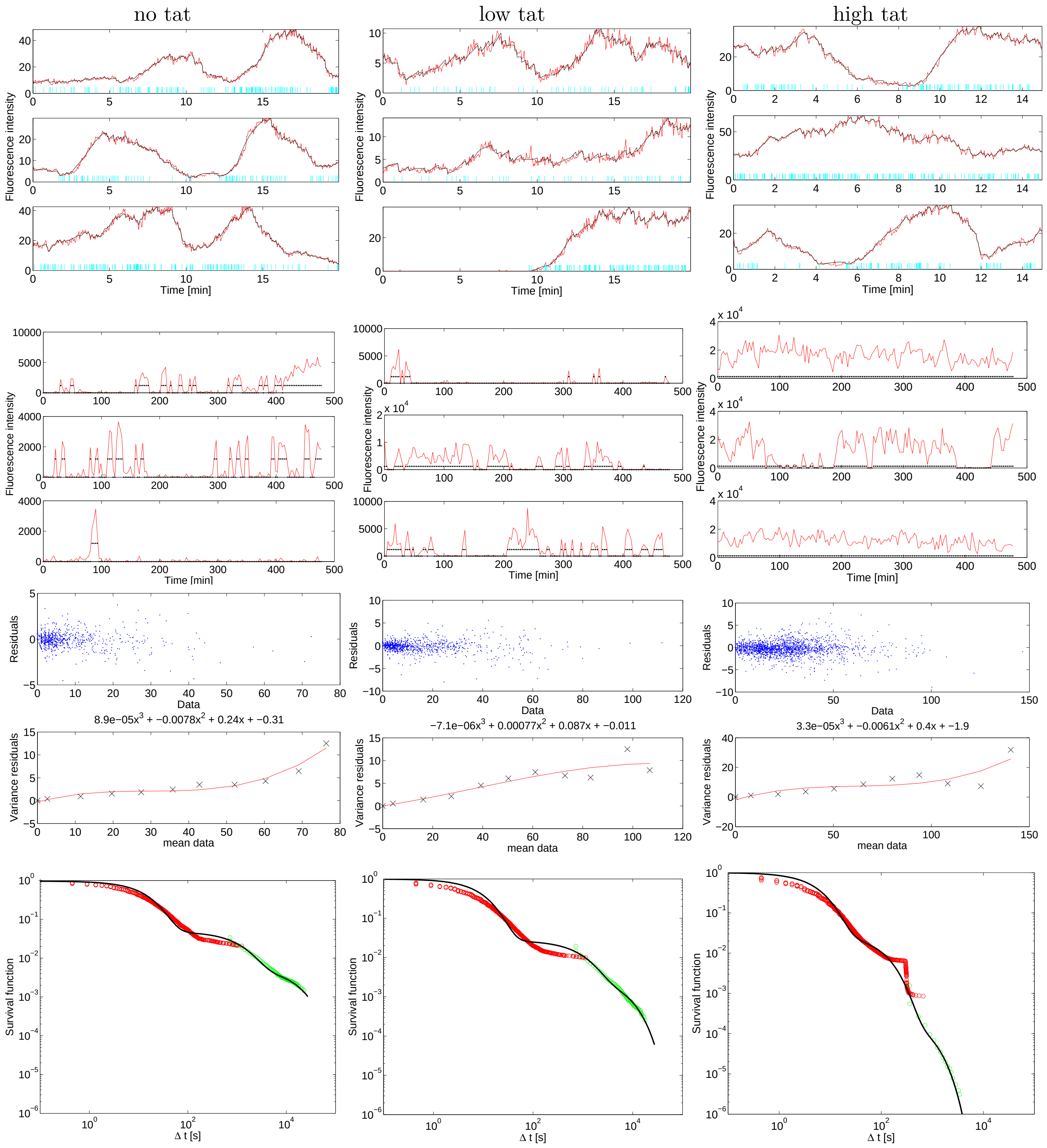

Figure S16: Results of the unconstrained three-exponential fit. First row: short movie data with reconstructed polymerase positions. Second row: long movie data. Third row: noise interpolation. Fourth row: most optimal fit for $\alpha = 0.30$.

4 Symbolic solution to the inverse problem

4.1 General model and waiting time distribution

We consider a continuous time Markov chain promoter model with N states $P_i, \quad i \in [1, N]$. One of these N states, P_o, is the "ON" state from which polymerase can start transcription, and all the other states are "OFF" states (non-processive). A supplementary state P_{N+1}, designates the start of processive elongation. From P_{N+1}, there is systematic return to P_o. The models have parameters $k_{i,j}, 1 \leq i, j \leq N+1$ indicating the transition rates from the promoter state i to the promoter state j. We consider that processive elongation immediately frees the operator and the promoter returns to the "ON" state. In mathematical terms

$$k_{N+1,o} = \infty. \tag{11}$$

We also consider that only one state, denoted X_N, can lead to processive elongation X_{N+1}: $k_{N,N+1} \neq 0$, $k_{i,N+1} = 0$, for $1 \leq i \leq N-1$. X_N is not necessarily X_o, for instance it can be a paused transcription state.

Because the movies are always started when transcription sites are in the active state and supposing that after each transcription initiation there is return to the active state, we model the experimental waiting time as the first time when the promoter reaches the state P_{N+1} starting from P_o. This is a first hitting time (or first passage time) problem. Because the lifetime of the state P_{N+1} is zero and P_{N+1} is always followed by P_o (see (11)), the same waiting time is also the first return time to P_o.

In order to compute the distribution of the first hitting time we use the following standard method.

Let $M(t)$ be the state of the Markov chain at the time t. For the purposes of this calculation, we can consider that $M(t)$ stops when it reaches P_{N+1}. Let $X_i = \mathbb{P}[M(t) = P_i | M(0) = P_o]$. Because $M(t)$ is stopped in P_{N+1}, one has $X_{N+1} = \mathbb{P}[M(t) = P_{N+1} | M(0) = P_o] = \mathbb{P}[\mathit{\Delta} \leq t]$. Thus, X_{N+1} is the cumulative distribution function of the waiting time $\mathit{\Delta}$ to reach P_{N+1} from P_o. The survival function of $\mathit{\Delta}$ is $S(t) = 1 - X_{N+1}(t)$.

The variables $X_i(t)$, $1 \leq i \leq N+1$, satisfy the following system of linear differential equations (the master equation):

$$\frac{d\boldsymbol{X}}{dt} = Q\boldsymbol{X}, \tag{12}$$

with the initial conditions $X_i(0) = \delta_{i,o}$, where δ is the Kronecker symbol; Q is the transpose transition rate matrix whose elements are defined by $Q_{j,i} = k_{i,j}$, $Q_{i,i} = -\sum_{j \neq i} k_{i,j}$.

Because $M(t)$ is stopped in P_{N+1}, the last column of the matrix Q is zero, namely $Q_{i,N+1} = 0$.

Let $\tilde{\boldsymbol{Q}}$ the $N \times N$ matrix obtained by eliminating the last line and the last column of the $(N+1) \times (N+1)$ matrix $\boldsymbol{Q}$.

Then $\tilde{\boldsymbol{X}} = (X_1, \ldots, X_N)$ is the solution of the reduced equation

$$\frac{d\tilde{\boldsymbol{X}}}{dt} = \tilde{\boldsymbol{Q}}\tilde{\boldsymbol{X}}, \tag{13}$$

with initial conditions $X_i = \delta_{i,o}$ and reads

$$\tilde{\boldsymbol{X}}(t) = \sum_{i=1}^{N} C_i \boldsymbol{u}_i e^{\lambda_i t}, \tag{14}$$

where λ_i and $\boldsymbol{u}_i$, $i \in [1, N]$ are eigenvalues and eigenvectors of $\tilde{\boldsymbol{Q}}$, respectively.

Although (14) is written with the non-degenerate case in mind, when $\lambda_1 \neq \lambda_2 \neq \ldots \neq \lambda_N$ (in general (14) is valid when $\tilde{Q}$ is diagonalizable), our final results, relating parameters of the survival function and kinetic parameters, can be extended to the degenerate case by continuous extension (see Section 4.2).

Furthermore, X_{N+1} can be obtained from the equation

$$\frac{dX_{N+1}}{dt} = k_{N,N+1} X_N, \tag{15}$$

with the initial condition $X_{N+1}(0) = 0$.

Without restricting generality, all eigenvectors $\boldsymbol{u}_i$ can be chosen such that their o-th coordinate is $u_o^i = 1$. Therefore, from (14), it follows

$$X_o = \sum_{i=1}^{N} C_i e^{\lambda_i t},$$

and from (15) it follows

$$X_{N+1}(t) = k_{N,N+1} \sum_{i=1}^{N} C_i u_N^i \frac{e^{\lambda_i t} - 1}{\lambda_i}. \tag{16}$$

From $\lim_{t\to\infty} X_{N+1}(t) = 1$ and (16) we get $k_{N,N+1} \sum_{i=1}^{N} \frac{C_i u_N^i}{\lambda_i} = -1$. Using again (16) we find

$$S(t) = 1 - X_{N+1}(t) = -k_{N,N+1} \sum_{i=1}^{N} \frac{C_i u_N^i e^{\lambda_i t}}{\lambda_i} = \sum_{i=1}^{N} A_i e^{\lambda_i t}. \tag{17}$$

Hence

$$A_i = -\frac{k_{N,N+1} u_N^i C_i}{\lambda_i}, \; 1 \leq i \leq N. \tag{18}$$

In particular, when $N = o$

$$A_i = -\frac{k_{o,N+1} C_i}{\lambda_i}, \; 1 \leq i \leq N. \tag{19}$$

Eq(17) implies that in the non-degenerate case the survival function is a combination of exponential functions, implying that the waiting time has a mixed exponential distribution. However, although the mixture coefficients satisfy $\sum_{i=1}^{N} A_i = 1$, they are not guaranteed positive in general (see Figure S15).

4.2 Degenerate case

The matrix $\tilde{\boldsymbol{Q}}$ is a linear function in the model parameters k_{ij}. According to classical results (see [7], Chap.2), the eigenvalues of this matrix are branches of analytic functions in the parameters with only algebraic singularities. Moreover, the number of distinct eigenvalues is constant with the exception of a zero measure set of parameter values where this number is different. Excluding the permanently degenerate case when the matrix $\tilde{\boldsymbol{Q}}$ has a number of distinct eigenvalues smaller than N almost everywhere, we may consider that $\tilde{\boldsymbol{Q}}$ has N distinct eigenvalues except in a finite number of parameter values where it is degenerate, i.e. where $\lambda_i = \lambda_j$ for at least two distinct indices $i \neq j$.

Each degenerate case is arbitrarily close in the parameter space to a non-degenerate case. The solutions of the linear differential system (12) are continuous in the transition rates parameters $k_{i,j}$, therefore the survival function computed in a degenerate case can be approximated by survival functions computed for non-degenerate cases. Because all the survival functions are monotone, by the second Dini's theorem, this approximation is uniform for $t \in [0, T]$, for any T. Using the inequality $|A_i \exp(\lambda_i t) - A_i' \exp(\lambda_i' t)| < C \exp(\lambda_i' T), \forall t > T$, where $\lambda_i \leq \lambda_i' < 0$, we can show that the uniform approximation is valid for all times.

Let us now compute the survival function in the degenerate case.

When, in spite of having degenerate eigenvalues (there are N independent eignevectors), the matrix $\tilde{\boldsymbol{Q}}$ is diagonalizable, then Eqs.(14),(16),(17) hold. Therefore, in the diagonalizable case with degenerate eigenvalues the survival function is a sum of less than N exponentials.

When the matrix $\tilde{\boldsymbol{Q}}$ is not diagonalizable (there are less than N independent eigenvectors), (14) no longer holds.

Let $g_i \leq m_i$ be the geometric multiplicity (number of independent eigenvectors) of the eigenvalue λ_i. Here m_i is the algebraic multiplicity of the eigenvalue λ_i, representing the number of times this eigenvalue occurs as a root of the characteristic polynomial ($\det(\tilde{\boldsymbol{Q}} - \lambda_i \boldsymbol{I}) \sim (\lambda - \lambda_i)^{m_i}$) and one has $\sum_i' m_i = N$, where the sum is over all distinct eigenvalues. Let us consider that $g_i < m_i$ for at least one i. In this situation, $\tilde{\boldsymbol{Q}}$ is not diagonalizable but can be reduced to a Jordan normal form. For each eigenvalue, there are g_i Jordan blocks. After reindexing the eigenvalues and Jordan blocks we have $N = \sum_{i=1}^{p} m_i$, where p is the total number of Jordan blocks $p = \sum_i' g_i$

(the sum is over distinct λ_i) and m_i is the dimension of a block i.

Let us remind that a generalized eigenvector $\boldsymbol{v}$ is any vector from the kernel $Ker((\tilde{\boldsymbol{Q}}-\lambda_i)^{m_i})$. The subspace $Ker((\tilde{\boldsymbol{Q}}-\lambda_i)^{m_i})$ corresponds to a Jordan block and is generated by a chain of generalized eigenvectors $\boldsymbol{u}_i$, $(\tilde{\boldsymbol{Q}}-\lambda_i)\boldsymbol{u}_i, \ldots, (\tilde{\boldsymbol{Q}}-\lambda_i)^{m_i-1}\boldsymbol{u}_i$, where $\boldsymbol{u}_i$ is a generalized vector that satisfies $(\tilde{\boldsymbol{Q}}-\lambda_i)\boldsymbol{u}_i \neq 0, \ldots, (\tilde{\boldsymbol{Q}}-\lambda_i)^{m_i-1}\boldsymbol{u}_i \neq 0$. Furthermore, the solution of (13) starting from any generalized vector $\boldsymbol{v}$ reads:

$$\tilde{\boldsymbol{X}}(t) = \exp(\lambda_i t)\sum_{j=1}^{m_i} \frac{t^{j-1}}{(j-1)!}(\tilde{\boldsymbol{Q}}-\lambda_i)^{j-1}\boldsymbol{v}. \tag{20}$$

Let us consider that

$$\tilde{\boldsymbol{X}}(0) = \sum_{i=1}^{p}\sum_{j=1}^{m_i} C_{i,j}(\tilde{\boldsymbol{Q}}-\lambda_i)^{j-1}\boldsymbol{u}_i.$$

By the definition of the generalized eigenvectors, $(\tilde{\boldsymbol{Q}}-\lambda_i)^{m_i}\boldsymbol{u}_i = 0$.

Therefore, in the non-diagonalizable case, (14) must be replaced by:

$$\tilde{\boldsymbol{X}}(t) = \sum_{i=1}^{p}\sum_{j=1}^{m_i}\sum_{k=1}^{m_i+1-j} C_{i,j}\exp(\lambda_i t)\frac{t^{k-1}}{(k-1)!}(\tilde{\boldsymbol{Q}}-\lambda_i)^{j+k-2}\boldsymbol{u}_i. \tag{21}$$

Then, (16) should be replaced by

$$X_{N+1} = k_{N,N+1}\int_0^t X_N(s)\,ds = k_{N,N+1}\sum_{i=1}^{p}\sum_{j=1}^{m_i}\sum_{k=1}^{m_i+1-j} C_{i,j}u_N^{i,j+k-2}\frac{\gamma(k,-\lambda_i t)}{(-\lambda_i)^k(k-1)!}, \tag{22}$$

where $u_N^{i,j}$ is the Nth coordinate of $(\tilde{\boldsymbol{Q}}-\lambda_i)^j\boldsymbol{u}_i$, and

$$\gamma(k,x) = \int_0^x u^{k-1}\exp(-u)\,du$$

is the incomplete gamma function.

It follows that (17) should be replaced by

$$S(t) = 1 - X_{N+1}(t) = k_{N,N+1}\sum_{i=1}^{p}\sum_{j=1}^{m_i}\sum_{k=1}^{m_i+1-j}\frac{C_{i,j}u_N^{i,j+k-2}}{(-\lambda_i)^k}\left[1-\frac{\gamma(k,-\lambda_i t)}{(k-1)!}\right]. \tag{23}$$

Eq. (23) implies that in the non-diagonalizable case, the survival function is a combination of gamma functions, implying that the waiting time has a mixed gamma distribution (of shape parameters $1 \leq a_i \leq m_i$ and scale parameters $b_i = -1/\lambda_i$). As a reminder, the cumulative density function of a gamma distribution of shape parameter a and scale parameter $b > 0$ is $\gamma(a, t/b)/(a-1)!$.

As an example illustrating the non-diagonalisable case let us consider the irreversible chain $P_1 \xrightarrow{k} P_2 \xrightarrow{k} P_3 \xrightarrow{k_{ini}} P_4$ where P_3 is the ON state and P_4 is the EL state. in this case we have

$$\tilde{\boldsymbol{Q}} = \begin{bmatrix} -k & 0 & 0 \\ k & -k & 0 \\ 0 & k & -k_{ini} \end{bmatrix}.$$

There are two distinct eigenvalues $\lambda_1 = -k$ and $\lambda_2 = -k_{ini}$. Each eigenvalue contributes with one Jordan block of dimensions 2 and 1, respectively. The chains of generalized eigenvectors are

$$\boldsymbol{u}_1 = \begin{bmatrix} -\frac{k-k_{ini}}{k} \\ 1 \\ 0 \end{bmatrix}, (\tilde{\boldsymbol{Q}} - \lambda_1)\boldsymbol{u}_1 = \begin{bmatrix} 0 \\ k_{ini} - k \\ k \end{bmatrix}, \boldsymbol{u}_2 = \begin{bmatrix} 0 \\ 0 \\ 1 \end{bmatrix}.$$

Suppose we want to compute the distribution of the waiting time to reach P_4 starting from P_1. Then

$$\tilde{\boldsymbol{X}}(0) = \begin{bmatrix} 1 \\ 0 \\ 0 \end{bmatrix} = -\frac{k}{k-k_{ini}} \begin{bmatrix} -\frac{k-k_{ini}}{k} \\ 1 \\ 0 \end{bmatrix} - \frac{k}{(k-k_{ini})^2} \begin{bmatrix} 0 \\ k_{ini} - k \\ k \end{bmatrix} + \frac{k^2}{(k-k_{ini})^2} \begin{bmatrix} 0 \\ 0 \\ 1 \end{bmatrix},$$

and

$$\tilde{\boldsymbol{X}}(t) = \exp(\tilde{\boldsymbol{Q}}t) \begin{bmatrix} 1 \\ 0 \\ 0 \end{bmatrix} = -\frac{k}{k-k_{ini}} e^{-kt} [\begin{bmatrix} -\frac{k-k_{ini}}{k} \\ 1 \\ 0 \end{bmatrix} + t \begin{bmatrix} 0 \\ k_{ini} - k \\ k \end{bmatrix}] - \frac{k}{(k-k_{ini})^2} e^{-kt} \begin{bmatrix} 0 \\ k_{ini} - k \\ k \end{bmatrix} + \frac{k^2}{(k-k_{ini})^2} e^{-k_{ini}t} \begin{bmatrix} 0 \\ 0 \\ 1 \end{bmatrix}.$$

$X_4(t)$ is the solution of $\frac{dX_4}{dt} = k_{ini} X_3(t)$ and read

$$X_4(t) = -\frac{k_{ini}}{k-k_{ini}} \gamma(2, kt) - \frac{k_{ini}k}{(k-k_{ini})^2}(1 - \exp(-kt)) + \frac{k^2}{(k-k_{ini})^2}(1 - \exp(-k_{ini}t)).$$

The survival function reads

$$S(t) = 1 - X_4(t) = A_1 [1 - \gamma(2, kt)] + A_2 \exp(-kt) + A_3 \exp(-k_{ini}t),$$

where $A_1 = -k_{ini}/(k - k_{ini})$, $A_2 = -k_{ini}k/(k - k_{ini})^2$, $A_3 = k^2/(k - k_{ini})^2$ satisfy $A_1 + A_2 + A_3 = 1$.

The waiting time is distributed according to a mixture of gamma and exponential distributions. If $k_{ini} >> k$, then $A_1 \approx 1$, $A_2, A_3 \approx 0$, meaning that the waiting time is distributed according to a gamma distribution of shape parameter 2 and scale parameter $1/k$.

If in the previous model we make $k_{ini} = k$,

$$\tilde{\boldsymbol{Q}} = \begin{bmatrix} -k & 0 & 0 \\ k & -k & 0 \\ 0 & k & -k \end{bmatrix}.$$

Then, $\tilde{\boldsymbol{Q}}$ has only one eigenvalue $\lambda = -k$ and one Jordan block of dimension 3. The chain of generalized eigenvectors is

$$\boldsymbol{u} = \begin{bmatrix} 1 \\ 0 \\ 0 \end{bmatrix}, (\tilde{\boldsymbol{Q}} + k\boldsymbol{I})\boldsymbol{u} = \begin{bmatrix} 0 \\ k \\ 0 \end{bmatrix}, (\tilde{\boldsymbol{Q}} + k\boldsymbol{I})^2\boldsymbol{u} = \begin{bmatrix} 0 \\ 0 \\ k^2 \end{bmatrix}$$

The survival function reads

$$S(t) = 1 - \frac{\gamma(3, kt)}{2!},$$

meaning that the waiting time is distributed according to a gamma distribution with scale parameter $1/k$ and shape parameter 3. This result is obvious from the structure of the model. If $k = k_{ini}$, in order to reach P_4 from P_1 one needs three exponentially distributed steps of equal mean time $1/k$; a sum of three independent equally distributed exponential variables is a gamma distribution of shape parameter 3.

In general, if the chain contains n limiting steps of constant k, the waiting time to reach the end of the chain starting from the beginning is distributed approximately according to a gamma distribution with shape parameter n and scale parameter $1/k$.

4.3 Inverse problem

In order to formulate a well posed inverse problem, we have to choose a structure of the model. The structure is defined by the directed graph G whose vertices are the promoter states and such that there is an edge from i to j if and only if $k_{i,j} \neq 0$. Thus the model structure specifies which transitions are allowed between the promoter states. We also need to specify which one of the promoter states is ON.

Given a model structure, the inverse problem consists in computing the kinetic constants $k_{i,j}, 1 \leq i, j \leq N$ and $k_{N,N+1}$ from the $2N-1$ parameters of the survival function. This is possible only if there are at most $2N-1$, kinetic constants. Uniqueness and thus well-posedness of the solution is possible only if there are exactly $2N-1$ parameters. However, not all models with $2N-1$ parameters have unique solutions of the inverse problem (an example is the model M3, see Figure S5 and Section 4.6).

In order to solve the inverse problem, we must write down the equations relating the parameters $k_{i,j}$, A_i and λ_i.

Let us consider that all the nonzero kinetic parameters are the $2N-1$ elements of a vector $\boldsymbol{k} \in \mathbb{R}^{2N-1}$.

Vieta's formulas

Let us introduce the elementary symmetric polynomials of eigenvalues

$$L_1 = \sum_{i=1}^{N} \lambda_i \tag{24}$$

$$L_2 = \sum_{i<j} \lambda_i \lambda_j \tag{25}$$

$$\vdots \tag{26}$$

$$L_N = \lambda_1 \lambda_2 \dots \lambda_N \tag{27}$$

The characteristic polynomial of $\tilde{\boldsymbol{Q}}$ is

$$P(\lambda) = det(\tilde{\boldsymbol{Q}} - \lambda \boldsymbol{I}) = (-1)^N \lambda^N + a_{N-1}(\boldsymbol{k})\lambda^{N-1} + \ldots + a_1(\boldsymbol{k})\lambda + a_0(\boldsymbol{k}) \tag{28}$$

where the coefficients a_i are multivariate polynomial functions of the kinetic constants.

The coefficients of the characteristic polynomial are related to the symmetric polynomials of eigenvalues by the so-called Vieta's formulas. We have the following N equations for the kinetic constants:

$$L_j = (-1)^{N-j} a_{N-j}(\boldsymbol{k}), \quad j \in [1, N] \tag{29}$$

Eigenvectors

The eigenvectors of $\tilde{\boldsymbol{Q}}$ are solutions of the system of linear equations $(\tilde{\boldsymbol{Q}} - \lambda \boldsymbol{I})\boldsymbol{u} = 0$ and are chosen of the form $\boldsymbol{u} = (u_1(\lambda, \boldsymbol{k}), \ldots, u_{o-1}(\lambda, \boldsymbol{k}), 1, u_{o+1}(\lambda, \boldsymbol{k}), \ldots, u_N(\lambda, \boldsymbol{k}))$, where $u_n(\lambda, \boldsymbol{k}), n \in [1, N-1]$ are rational functions (ratios of polynomials) of λ and $\boldsymbol{k}$.

The initial conditions satisfied by the variables X_i provide a linear system of equations for the constants C_i:

$$\sum_{j=1}^{N} u_i(\lambda_j, \boldsymbol{k}) C_j = \delta_{i,o}, \quad i \in [1, N] \tag{30}$$

Let $C_i(\boldsymbol{\lambda}, \boldsymbol{k}), \quad i \in [1, N]$ be the unique solution of (30).

From (18),(19) we get $N-1$ equations for the kinetic constants $\boldsymbol{k}$:

$$C_i(\boldsymbol{\lambda}, \boldsymbol{k}) = -A_i \lambda_i / (u_o^i k_{N,N+1}), \quad i \in [1, N-1] \tag{31}$$

Inverse problem

The solution of the inverse problem is the solution of the system of $2N-1$ equations (29) and (31).

In the next sections we solve this system symbolically. When a solution of the inverse problem exists, the kinetic parameters $k_{i,j}$ can be expressed as functions in λ_i and A_i. These functions are symmetric in the pairs (λ_i, A_i) and homogeneous of degree -1 in λ_i. These functions are not always rational. For instance, they can have branching singularities, allowing, eventually, to pass from one solution to another, equivalent one. In general, multiple solutions are equivalent with respect to symmetries of the model. For instance, the model M2 in the Figure S5 is symmetric with respect to the permutation of the two lateral chains. In this case there are two solutions of the inverse problem, one solution being obtained from the other by permuting the parameters $k_1^{\pm}$ with $k_2^{\pm}$. The general solutions will be presented elsewhere. In the sequel we provide full solutions for some models with $N \leq 4$.

Recursion relations for eigenvectors

The eigenvector components $u_i(\lambda_j, \boldsymbol{k})$ can be obtained by recursion along the structure digraph.

We consider models such that any state of the promoter is connected to the ON state, in both directions, by directed paths on the structure digraph.

In the sequel, we discuss two representative cases.

The type I (*single chain*) model is a reversible chain ending with the P_N state:

$$P_1 \underset{k_1^-}{\overset{k_1^+}{\rightleftharpoons}} P_2 \underset{k_2^-}{\overset{k_2^+}{\rightleftharpoons}} P_3 \ldots P_{N-1} \underset{k_{N-1}^-}{\overset{k_{N-1}^+}{\rightleftharpoons}} P_N \overset{k_N}{\rightarrow}$$

For this model, an eigenvector $(b_1, b_2, \ldots, b_N)$ satisfies the equations

$$k_1^- b_2 - (k_1^+ + \lambda) b_1 = 0, \tag{32}$$

$$k_{n-1}^+ b_{n-1} + k_n^- b_{n+1} - (k_n^+ + k_{n-1}^- + \lambda) b_n = 0, \text{for } 2 \leq n \leq N-1. \tag{33}$$

We can choose $b_1 = 1$ and then from (32) $b_2 = \frac{k_1^+ + \lambda}{k_1^-}$. Threfore, b_n satisfy the recursion

$$\begin{aligned} b_1 &= 1, b_2 = \frac{k_1^+ + \lambda}{k_1^-} \\ b_{n+1} &= \frac{k_n^+ + k_{n-1}^- + \lambda}{k_n^-} b_n - \frac{k_{n-1}^+}{k_n^-} b_{n-1}, 2 \leq n \leq N-1. \end{aligned} \tag{34}$$

In order to have $u_o^i = 1$ for all $1 \leq i \leq N$, we define

$$u_n(\lambda, \boldsymbol{k}) = \frac{b_n(\lambda, \boldsymbol{k})}{b_o(\lambda, \boldsymbol{k})}, \quad n \in [1, N], \tag{35}$$

where b_n are rational functions of λ and $\boldsymbol{k}$ computed with the recursion (34).

The type II model is a reversible chain with the P_N state inside the chain:

$$P_1 \underset{k_1^-}{\overset{k_1^+}{\rightleftharpoons}} P_2 \dots P_{N-r} \underset{k_{N-r}^-}{\overset{k_{N-r}^+}{\rightleftharpoons}} P_N \overset{k_N}{\uparrow} \underset{k_{N-r+1}^-}{\overset{k_{N-r+1}^+}{\rightleftharpoons}} P_{N-r+1} \dots P_{N-2} \underset{k_{N-1}^-}{\overset{k_{N-1}^+}{\rightleftharpoons}} P_{N-1}$$

The type II model can be also described as two reversible chains branching from the P_N state. We can call this type *two chain model*.

For this model, an eigenvector $(b_1, b_2, \dots, b_N)$ satisfies the recursion

$$k_1^- b_2 - (k_1^+ + \lambda) b_1 = 0, \tag{36}$$

$$k_{n-1}^+ b_{n-1} + k_n^- b_{n+1} - (k_n^+ + k_{n-1}^- + \lambda) b_n = 0, \text{for } 2 \le n \le N - r, \tag{37}$$

$$k_n^+ b_{n-1} + k_{n+1}^- b_{n+1} - (k_{n-1}^+ + k_n^- + \lambda) b_n = 0, \text{for } N - r + 1 \le n \le N - 2, \tag{38}$$

$$k_{N-1}^+ b_{N-2} - (k_{N-1}^- + \lambda) b_{N-1} = 0, \tag{39}$$

The recursion (36),(37),(38),(39) can be solved in the following way:

i) Choose $b_1 = 1$ and compute b_2 from (36).

ii) Use (37) to compute b_n, $3 \le n \le N - r$ and b_N.

iii) Use (39) and (38) to compute b_n, $N - r + 1 \le n \le N - 1$ and b_N as multiples of b_{N-1}.

iv) Determine b_{N-1} from b_N, already computed at step ii).

Below we study several examples of type I and type II models.

4.4 Symbolic solution to the inverse problem for the M1 model ($N = 3$)

This model is described by the transitions $P_1 \underset{k_1^-}{\overset{k_1^+}{\rightleftharpoons}} P_2 \underset{k_2^-}{\overset{k_2^+}{\rightleftharpoons}} P_3 \overset{k_3}{\rightarrow}$. It is a type I model. In this case P_3 is the ON state. The matrix of kinetic rates reads

$$\tilde{Q} = \begin{bmatrix} -k_1^+ & k_1^- & 0 \\ k_1^+ & -(k_2^+ + k_1^-) & k_2^- \\ 0 & k_2^+ & -(k_3 + k_2^-) \end{bmatrix}.$$

The characteristic polynomial of $\tilde{\boldsymbol{Q}}$ is $P(\lambda) = det(\tilde{\boldsymbol{Q}} - \lambda \boldsymbol{I}) = -\lambda^3 - (k_3 + k_1^- + k_2^- + k_1^+ + k_2^+)\lambda^2 - (k_3 k_1^- + k_3 k_1^+ + k_3 k_2^+ + k_1^- k_2^- + k_2^- k_1^+ + k_1^+ k_2^+)\lambda - k_3 k_1^+ k_2^+$.

The Vieta formulas read

$$k_3 k_1^+ k_2^+ = -L_3 \tag{40}$$

$$k_3 k_1^- + k_3 k_1^+ + k_3 k_2^+ + k_1^- k_2^- + k_2^- k_1^+ + k_1^+ k_2^+ = L_2 \tag{41}$$

$$k_3 + k_1^- + k_2^- + k_1^+ + k_2^+ = -L_1 \tag{42}$$

The solution of the recursion (34) is

$$b_1 = 1 \tag{43}$$

$$b_2 = \frac{k_1^+ + \lambda}{k_1^-} \tag{44}$$

$$b_3 = \frac{k_1^+ k_2^+ + (k_1^- + k_1^+ + k_2^+)\lambda + \lambda^2}{k_1^- k_2^-} \tag{45}$$

The system (30) has the solution

$$\begin{aligned}
C_1 &= \frac{k_1^+ k_2^+ + (k_1^- + k_1^+ + k_2^+)\lambda_1 + \lambda_1^2}{(\lambda_1 - \lambda_2)(\lambda_1 - \lambda_3)} \\
C_2 &= \frac{k_1^+ k_2^+ + (k_1^- + k_1^+ + k_2^+)\lambda_2 + \lambda_2^2}{(\lambda_2 - \lambda_1)(\lambda_2 - \lambda_3)} \\
C_3 &= \frac{k_1^+ k_2^+ + (k_1^- + k_1^+ + k_2^+)\lambda_3 + \lambda_3^2}{(\lambda_3 - \lambda_1)(\lambda_3 - \lambda_2)}
\end{aligned}$$

The unique solution of (29) and (31) is

$$k_3 = -S_1, \tag{46}$$

$$k_2^+ = \frac{S_2^2 - S_1 S_3}{S_1(-S_1^2 + S_2)}, \tag{47}$$

$$k_2^- = S_1 - \frac{S_2}{S_1}, \tag{48}$$

$$k_1^+ = \frac{L_3(-S_1^2 + S_2)}{S_2^2 - S_1 S_3}, \tag{49}$$

$$k_1^- = \frac{A_1 A_2 A_3 S_1 (\lambda_1 - \lambda_2)^2 (\lambda_1 - \lambda_3)^2 (\lambda_2 - \lambda_3)^2}{(-S_1^2 + S_2)(S_2^2 - S_1 S_3)}, \tag{50}$$

where

$$
\begin{aligned}
L_1 &= \lambda_1 + \lambda_2 + \lambda_3, & (51)\\
L_2 &= \lambda_1\lambda_2 + \lambda_1\lambda_3 + \lambda_2\lambda_3, & (52)\\
L_3 &= \lambda_1\lambda_2\lambda_3, & (53)\\
S_1 &= A_1\lambda_1 + A_2\lambda_2 + A_3\lambda_3, & (54)\\
S_2 &= A_1\lambda_1^2 + A_2\lambda_2^2 + A_3\lambda_3^2, & (55)\\
S_3 &= A_1\lambda_1^3 + A_2\lambda_3^3 + A_3\lambda_3^3. & (56)
\end{aligned}
$$

4.5 Symbolic solution to the inverse problem for the M2 model ($N = 3$)

This model is described by the transitions $P_1 \underset{k_1^-}{\overset{k_1^+}{\rightleftharpoons}} P_3 \overset{k_3}{\uparrow} \underset{k_2^+}{\overset{k_2^-}{\rightleftharpoons}} P_2$. In this case P_3 is the ON state. Model M2 is a type II model. It has a matrix of kinetic rates

$$
\tilde{Q} = \begin{bmatrix} -k_1^+ & 0 & k_1^- \\ 0 & -k_2^+ & k_2^- \\ k_1^+ & k_2^+ & -(k_3 + k_1^- + k_2^-) \end{bmatrix}.
$$

The characteristic polynomial of $\tilde{\boldsymbol{Q}}$ is $P(\lambda) = det(\tilde{\boldsymbol{Q}} - \lambda \boldsymbol{I}) = -\lambda^3 - (k_3 + k_1^- + k_2^- + k_1^+ + k_2^+)\lambda^2 - (k_3k_1^+ + k_3k_2^+ + k_1^-k_2^+ + k_2^-k_1^+ + k_1^+k_2^+)\lambda - k_3k_1^+k_2^+$.

The Vieta formulas read

$$k_3k_1^+k_2^+ = -L_3 \tag{57}$$

$$k_3k_1^+ + k_3k_2^+ + k_1^-k_2^+ + k_2^-k_1^+ + k_1^+k_2^+ = L_2 \tag{58}$$

$$k_3 + k_1^- + k_2^- + k_1^+ + k_2^+ = -L_1 \tag{59}$$

The solution of the recursion (36),(37),(38),(39) reads

$$b_1 = 1 \tag{60}$$

$$b_2 = \frac{k_2^-(k_1^+ + \lambda)}{k_1^-(k_2^+ + \lambda)} \tag{61}$$

$$b_3 = \frac{k_1^+ + \lambda}{k_1^-} \tag{62}$$

The system (30) has the solution

$$
\begin{aligned}
C_1 &= \frac{k_1^+ k_2^+ + (k_1^+ + k_2^+)\lambda_1 + \lambda_1^2}{(\lambda_1 - \lambda_2)(\lambda_1 - \lambda_3)} \\
C_2 &= \frac{k_1^+ k_2^+ + (k_1^+ + k_2^+)\lambda_2 + \lambda_2^2}{(\lambda_2 - \lambda_1)(\lambda_2 - \lambda_3)} \\
C_3 &= \frac{k_1^+ k_2^+ + (k_1^+ + k_2^+)\lambda_3 + \lambda_3^2}{(\lambda_3 - \lambda_1)(\lambda_3 - \lambda_2)}
\end{aligned}
$$

Up to the permution symmetry $P_1 \leftrightarrow P_2$ the solution of (29) and (31) is unique and described by

$$
\begin{aligned}
k_3 &= -S_1, && (63) \\
k_2^+ &= \frac{1}{2}\left[-L_1 + \frac{S_2}{S_1} - \frac{\sqrt{(S_1L_1 - S_2)^2 - 4L_3S_1}}{S_1}\right], && (64) \\
k_2^- &= \frac{1}{2}\left[S_1 - \frac{S_2}{S_1} + \frac{-S_1^2L_1 + S_1S_2 + S_1L_2 - L_3 + \frac{S_2^2}{S_1} - S_3}{\sqrt{(S_1L_1 - S_2)^2 - 4L_3S_1}}\right], && (65) \\
k_1^+ &= \frac{1}{2}\left[-L_1 + \frac{S_2}{S_1} + \frac{\sqrt{(S_1L_1 - S_2)^2 - 4L_3S_1}}{S_1}\right], && (66) \\
k_1^- &= \frac{1}{2}\left[S_1 - \frac{S_2}{S_1} - \frac{-S_1^2L_1 + S_1S_2 + S_1L_2 - L_3 + \frac{S_2^2}{S_1} - S_3}{\sqrt{(S_1L_1 - S_2)^2 - 4L_3S_1}}\right], && (67)
\end{aligned}
$$

4.6 Symbolic solution to the inverse problem for the M3 model ($N = 3$)

The chain without P_4 is the same as the model M1. Like M1, M3 is a type I model. The difference is the position of the ON state which is in the middle of the chain (P_2 is the ON state).

The matrix $\tilde{Q}$ and its characteristic polynomial are the same as in the section 4.4. In particular, the Vieta relations remain the same:

$$
\begin{aligned}
k_3 k_1^+ k_2^+ &= -L_3, \\
k_3k_1^- + k_3k_1^+ + k_3k_2^+ + k_1^-k_2^- + k_2^-k_1^+ + k_1^+k_2^+ &= L_2, \\
k_3 + k_1^- + k_2^- + k_1^+ + k_2^+ &= -L_1. \qquad (68)
\end{aligned}
$$

However, instead of computing the waiting time for reaching P_4 starting from P_3, we compute the waiting time for reaching P_4 starting from P_2. In this model, the significance of the states P_2 and P_3 is ON and PAUSE, respectively. The observed waiting time is from ON to EL, therefore from P_2 to P_4.

We look for solutions of the master equation (13) with initial conditions $\boldsymbol{X}(0) = (0, 1, 0, 0)$.

Like in section in order to compute solutions of (13) we need the eigenvectors of $\tilde{Q}$. For the new initial conditions it is convenient to impose the normalization condition $u_2 = 1$, where u_i, $1 \leq i \leq 3$ are the components of the eigenvector $\boldsymbol{u}$. We get

$$u_1 = k_1^-/(k_1^+ + \lambda), \tag{69}$$

$$u_2 = 1, \tag{70}$$

$$u_3 = k_2^+/(k_3 + k_2^- + \lambda). \tag{71}$$

A solution of the (13) reads $\boldsymbol{X}(t) = C_1\boldsymbol{u}_1 \exp(\lambda_1 t) + C_2\boldsymbol{u}_2 \exp(\lambda_2 t) + C_3\boldsymbol{u}_3 \exp(\lambda_3 t)$. From the initial conditions, it follows

$$\begin{gathered} C_1\tfrac{k_1^-}{k_1^+ + \lambda_1} + C_2\tfrac{k_1^-}{k_1^+ + \lambda_2} + C_3\tfrac{k_1^-}{k_1^+ + \lambda_3} = 0, \\ C_1 + C_2 + C_3 = 1, \\ C_1\tfrac{k_2^+}{k_3 + k_2^- + \lambda_1} + C_2\tfrac{k_2^+}{k_3 + k_2^- + \lambda_2} + C_3\tfrac{k_2^+}{k_3 + k_2^- + \lambda_3} = 0. \end{gathered} \tag{72}$$

The system (72) has the solution

$$\begin{aligned} C_1 &= \frac{(k_1^+ + \lambda_1)(k_3 + k_2^- + \lambda_1)}{(\lambda_1 - \lambda_2)(\lambda_1 - \lambda_3)}, \\ C_2 &= -\frac{(k_1^+ + \lambda_2)(k_3 + k_2^- + \lambda_2)}{(\lambda_1 - \lambda_2)(\lambda_2 - \lambda_3)}, \\ C_3 &= \frac{(k_1^+ + \lambda_3)(k_3 + k_2^- + \lambda_3)}{(\lambda_1 - \lambda_3)(\lambda_2 - \lambda_3)}. \end{aligned} \tag{73}$$

X_4 obeys $\frac{dX_4}{dt} = k_3 X_3$ and the survival function is

$$s(t) = \sum_{i=1}^{3} A_i exp(\lambda_i t) = 1 - X_3 = -\sum_{i=1}^{3} \frac{C_i k_2^+}{k_3\lambda_i(k_3 + k_2^- + \lambda_i)} exp(\lambda_i t).$$

The relation between C_i and A_i reads:

$$-\lambda_i A_i = k_3 C_i u_3(\lambda_i) = k_3 C_i k_2^+/(k_3 + k_2^- + \lambda_i). \tag{74}$$

By definition $s(0) = 1$, therefore

$$A_1 + A_2 + A_3 = 1. \tag{75}$$

Using (74) and (29) we can show that

$$A_1\lambda_1 + A_2\lambda_2 + A_3\lambda_3 = 0. \tag{76}$$

Eq.76 is very important. It implies that in this case, instead of 5 independent parameters, the survival function has only 4 independent parameters A_1, λ_1, λ_2, λ_3. Using (76) and (75) we can compute the remaining parameters as

$$\begin{aligned} A_2 &= -\frac{\lambda_3 + A_1(\lambda_1 - \lambda_3)}{\lambda_2 - \lambda_3}, \\ A_3 &= \frac{\lambda_2 + A_1(\lambda_1 - \lambda_2)}{\lambda_2 - \lambda_3}. \end{aligned} \tag{77}$$

If the condition (76) is not satisfied, then the system formed from eqs. (68) and (74) (with $i = 1, 2$) is incompatible.

If the condition (76) is satisfied, then the system (68), (74) is indeterminate and has an infinity of solutions. In this case, all the solutions can be expressed as functions of a free parameter. In the sequel we will choose k_3 as free parameter. This choice leads to the following symmetric expressions:

$$\begin{aligned} k_1^+ &= L_3/S_2, \\ k_2^+ &= -S_2/k_3, \\ k_2^- &= \frac{S_2 - 2k_3^2 - k_3 L_1 \pm \sqrt{k_3(k_3(L_1^2 - 4L_2 - 4S_2) - 2S_3 - 2L_3) + S_2^2}}{2k3}, \\ k_1^- &= -k_3 - S_3/S_2 + S_2/k_3 - k_2^-. \end{aligned} \tag{78}$$

4.7 Symbolic solution to the inverse problem for the two state ON-OFF model ($N = 2$)

The two states ON-OFF model (telegraph model) reads $P_1 \underset{k_1^-}{\overset{k_1^+}{\rightleftharpoons}} P_2 \overset{k_2}{\rightarrow}$.

In order to identify this model we use a two exponential fit of the survival function $S(t) = A_1 \exp(\lambda_1 t) + A_2 \exp(\lambda_2 t)$. Without restricting the generality, we can consider that $\lambda_1 < \lambda_2 < 0$. Then, from $S'(t) \leq 0$ it follows $\frac{\lambda_2}{\lambda_2 - \lambda_1} \leq A_1 \leq 1$, $A_2 = 1 - A_1$.

From the parameters of the survival function we can compute the model parameters as follows

$$\begin{aligned} S_1 &= A_1\lambda_1 + A_2\lambda_2, \\ S_2 &= A_1\lambda_1^2 + A_2\lambda_2^2, \\ S_3 &= A_1\lambda_1^3 + A_2\lambda_2^3, \\ k_2 &= -S_1, \\ k_1^- &= S_1 - S_2/S_1, \\ k_1^+ &= (S_3 S_1 - S_2^2)/S_1/(S_1^2 - S_2). \end{aligned} \tag{79}$$

4.8 Symbolic solution to the inverse problem for the four state chain model ($N = 4$)

This model is described by the transitions

$$P_1 \underset{k_1^-}{\overset{k_1^+}{\rightleftharpoons}} P_2 \underset{k_2^-}{\overset{k_2^+}{\rightleftharpoons}} P_3 \underset{k_3^-}{\overset{k_3^+}{\rightleftharpoons}} P_4 \overset{k_4}{\to} .$$

In this case P_4 is the ON state. The model is of type I.

We have

$$\tilde{Q} = \begin{bmatrix} -k_1^+ & k_1^- & 0 & 0 \\ k_1^+ & -(k_2^+ + k_1^-) & k_2^- & 0 \\ 0 & k_2^+ & -(k_3^+ + k_2^-) & k_3^- \\ 0 & 0 & k_3^+ & -(k_4 + k_3^-) \end{bmatrix}.$$

The Vieta formulas read

$$k_4 k_1^+ k_2^+ k_3^+ = L_4 \tag{80}$$

$$k_4 k_1^- k_2^- + k_4 k_2^- k_1^+ + k_4 k_1^- k_3^+ + k_4 k_1^+ k_2^+ + k_4 k_1^+ k_3^+ + k_4 k_2^+ k_3^+ + k_1^- k_2^- k_3^- + k_2^- k_3^- k_1^+ + k_3^- k_1^+ k_2^+ +$$
$$+ k_1^+ k_2^+ k_3^+ = -L_3 \tag{81}$$

$$k_4 k_1^- + k_4 k_2^- + k_4 k_1^+ + k_4 k_2^+ + k_4 k_3^+ + k_1^- k_2^- + k_1^- k_3^- + k_2^- k_3^- + k_2^- k_1^+ + k_1^- k_3^+ + k_3^- k_1^+ + k_3^- k_2^+ +$$
$$+ k_1^+ k_2^+ + k_1^+ k_3^+ + k_2^+ k_3^+ = L_2 \tag{82}$$

$$k_4 + k_1^- + k_2^- + k_3^- + k_1^+ + k_2^+ + k_3^+ = -L_1 \tag{83}$$

The solution of the recursion (34) is

$$b_1 = 1 \tag{84}$$

$$b_2 = \frac{k_1^+ + \lambda}{k_1^-} \tag{85}$$

$$b_3 = \frac{k_1^+ k_2^+ + (k_1^- + k_1^+ + k_2^+)\lambda + \lambda^2}{k_1^- k_2^-} \tag{86}$$

$$b_4 = \frac{\lambda^3 + (k_1^- + k_2^- + k_1^+ + k_2^+ + k_3^+)\lambda^2 + (k_1^- k_2^- + k_2^- k_1^+ + k_1^- k_3^+ + k_1^+ k_2^+ + k_1^+ k_3^+ + k_2^+ k_3^+)\lambda + k_1^+ k_2^+ k_3^+}{k_1^- k_2^- k_3^-} \tag{87}$$

The system (30) has the solution

$$C_1 = \frac{\lambda_1^3 + (k_1^- + k_2^- + k_1^+ + k_2^+ + k_3^+)\lambda_1^2 + (k_1^- k_2^- + k_2^- k_1^+ + k_1^- k_3^+ + k_1^+ k_2^+ + k_1^+ k_3^+ + k_2^+ k_3^+)\lambda_1 + k_1^+ k_2^+ k_3^+}{(\lambda_1 - \lambda_2)(\lambda_1 - \lambda_3)(\lambda_1 - \lambda_4)}$$

$$C_2 = \frac{\lambda_2^3 + (k_1^- + k_2^- + k_1^+ + k_2^+ + k_3^+)\lambda_2^2 + (k_1^- k_2^- + k_2^- k_1^+ + k_1^- k_3^+ + k_1^+ k_2^+ + k_1^+ k_3^+ + k_2^+ k_3^+)\lambda_2 + k_1^+ k_2^+ k_3^+}{(\lambda_2 - \lambda_1)(\lambda_2 - \lambda_3)(\lambda_2 - \lambda_4)}$$

$$C_3 = \frac{\lambda_3^3 + (k_1^- + k_2^- + k_1^+ + k_2^+ + k_3^+)\lambda_3^2 + (k_1^- k_2^- + k_2^- k_1^+ + k_1^- k_3^+ + k_1^+ k_2^+ + k_1^+ k_3^+ + k_2^+ k_3^+)\lambda_3 + k_1^+ k_2^+ k_3^+}{(\lambda_3 - \lambda_1)(\lambda_3 - \lambda_2)(\lambda_3 - \lambda_4)}$$

The eqs. (29) and (31) provide

$$\begin{aligned}
k_4 &= -S_1 && (88)\\
k_3^+ &= (S_2^2 - S_1S_3)/(-S_1^3 + S_2S_1) && (89)\\
k_3^- &= S_1 - S_2/S_1 && (90)\\
k_2^+ &= -\frac{(-S_1^2 + S_2)P_9}{(-S_2^2 + S_1S_3)P_6} && (91)\\
k_2^- &= \frac{S_1P_6}{(-S_2^2 + S_1S_3)(-S_1^2 + S_2)} && (92)\\
k_1^+ &= -\frac{L_4P_6}{P_9} && (93)\\
k_1^- &= \frac{(-S_2^2 + S_1S_3)P_{12}}{P_6P_9} && (94)
\end{aligned}$$

where $S_n = \sum_{i=1}^4 A_i\lambda_i^n$,

$L_n = \sum_{1\leq i_1<i_2<\ldots<i_n\leq 4} \lambda_{i_1}\lambda_{i_2}\ldots\lambda_{i_n}$,$P_6 = L_3S_1^3 - L_2S_1^2S_2 + L_1S_1^2S_3 - L_4S_1^2 - 2S_1S_2S_3 - L_3S_1S_2 + S_2^3 + L_2S_2^2 - L_1S_2S_3 + L_4S_2 + S_3^2$,

$P_9 = L_1^2S_2^2S_3 - L_1L_2S_1S_2S_3 - L_1L_2S_2^3 + L_1L_3S_1^2S_3 + L_1L_3S_1S_2^2 - L_1L_4S_1S_3 - L_1L_4S_2^2 - 2L_1S_2S_3^2 + L_2^2S_1S_2^2 - 2L_2L_3S_1^2S_2 + 2L_2L_4S_1S_2 + L_2S_1S_3^2 + L_2S_2^2S_3 + L_3^2S_1^3 - 2L_3L_4S_1^2 - 3L_3S_1S_2S_3 + L_3S_2^3 + L_4^2S_1 + L_4S_1^2S_3 - L_4S_1S_2^2 + 2L_4S_2S_3 + S_3^3$,

$P_{12} = L_1^3S_2^3S_3 - 2L_1^2L_2S_1S_2^2S_3 - L_1^2L_2S_2^4 + L_1^2L_3S_1^2S_2S_3 + L_1^2L_3S_1S_2^3 + L_1^2L_3S_2^2S_3 + L_1^2L_4S_1^3S_3 - 3L_1^2L_4S_1S_2S_3 - L_1^2L_4S_2^3 - 3L_1^2S_2^2S_3^2 + L_1L_2^2S_1^2S_2S_3 + 2L_1L_2^2S_1S_2^3 - L_1L_2L_3S_1^3S_3 - 3L_1L_2L_3S_1^2S_2^2 - L_1L_2L_3S_1S_2S_3 - L_1L_2L_3S_2^3 - L_1L_2L_4S_1^3S_2 + L_1L_2L_4S_1^2S_3 + 5L_1L_2L_4S_1S_2^2 + L_1L_2L_4S_2S_3 + 4L_1L_2S_1S_2S_3^2 + 2L_1L_2S_2^3S_3 + L_1L_3^2S_1^3S_2 + L_1L_3^2S_1^2S_3 + L_1L_3^2S_1S_2^2 + L_1L_3L_4S_1^4 - 4L_1L_3L_4S_1^2S_2 - 2L_1L_3L_4S_1S_3 - L_1L_3L_4S_2^2 - L_1L_3S_1^2S_3^2 - 4L_1L_3S_1S_2^2S_3 + L_1L_3S_2^4 - 2L_1L_3S_2S_3^2 - L_1L_4^2S_1^3 + 3L_1L_4^2S_1S_2 + L_1L_4^2S_3 - L_1L_4S_1^2S_2S_3 - L_1L_4S_1S_2^3 + 3L_1L_4S_1S_3^2 + 5L_1L_4S_2^2S_3 + 3L_1S_2S_3^3 - L_2^3S_1^2S_2^2 + 2L_2^2L_3S_1^3S_2 + L_2^2L_3S_1S_2^2 - 2L_2^2L_4S_1^2S_2 - L_2^2L_4S_2^2 - L_2^2S_1^2S_3^2 - 2L_2^2S_1S_2^2S_3 - L_2L_3^2S_1^4 - 2L_2L_3^2S_1^2S_2 + 2L_2L_3L_4S_1^3 + 4L_2L_3L_4S_1S_2 + 5L_2L_3S_1^2S_2S_3 - L_2L_3S_1S_2^3 + L_2L_3S_1S_3^2 + L_2L_3S_2^2S_3 - L_2L_4^2S_1^2 - 2L_2L_4^2S_2 - 2L_2L_4S_1^3S_3 + 3L_2L_4S_1^2S_2^2 - 4L_2L_4S_1S_2S_3 - 2L_2L_4S_2^3 - L_2L_4S_3^2 - 2L_2S_1S_3^3 - L_2S_2^2S_3^2 + L_3^3S_1^3 - 3L_3^2L_4S_1^2 - L_3^2S_1^3S_3 - 3L_3^2S_1S_2S_3 + L_3^2S_2^3 + 3L_3L_4^2S_1 - L_3L_4S_1^3S_2 + 5L_3L_4S_1^2S_3 - L_3L_4S_1S_2^2 + 3L_3L_4S_2S_3 + 3L_3S_1S_2S_3^2 - L_3S_2^3S_3 + L_3S_3^3 - L_4^3 - L_4^2S_1^4 + 4L_4^2S_1^2S_2 - 4L_4^2S_1S_3 - 2L_4^2S_2^2 - 2L_4S_1^2S_3^2 + 4L_4S_1S_2^2S_3 - L_4S_2^4 - 4L_4S_2S_3^2 - S_3^4$.

Using the relation $A_1 + A_2 + A_3 + A_4 = 1$, the above expressions can be simplified to

$P_6 = -(\lambda_1 - \lambda_3)(\lambda_2 - \lambda_3)(\lambda_1 - \lambda_4)(\lambda_2 - \lambda_4)(\lambda_3 - \lambda_4)^2A_3A_4(A_1 + A_2) - (\lambda_1 - \lambda_2)(\lambda_3 - \lambda_2)(\lambda_1 - \lambda_4)(\lambda_3 - \lambda_4)(\lambda_2 - \lambda_4)^2A_2A_4(A_1 + A_3) - (\lambda_1 - \lambda_2)(\lambda_4 - \lambda_2)(\lambda_1 - \lambda_3)(\lambda_4 - \lambda_3)(\lambda_2 - \lambda_3)^2A_2A_3(A_1 + A_4) - (\lambda_2 - \lambda_1)(\lambda_3 -$

$\lambda_1)(\lambda_2-\lambda_4)(\lambda_3-\lambda_4)(\lambda_1-\lambda_4)^2A_1A_4(A_2+A_3)-(\lambda_2-\lambda_1)(\lambda_4-\lambda_1)(\lambda_2-\lambda_3)(\lambda_4-\lambda_3)(\lambda_1-\lambda_3)^2A_1A_3(A_2+A_4)-(\lambda_3-\lambda_1)(\lambda_4-\lambda_1)(\lambda_3-\lambda_2)(\lambda_4-\lambda_2)(\lambda_1-\lambda_2)^2A_1A_2(A_3+A_4),$

$P_{12}=-A_1A_2A_3A_4(\lambda_1-\lambda_2)^2(\lambda_1-\lambda_3)^2(\lambda_1-\lambda_4)^2(\lambda_2-\lambda_3)^2(\lambda_2-\lambda_4)^2(\lambda_3-\lambda_4)^2.$

4.9 Symbolic solution to the inverse problem for the four state model with branching ($N = 4$)

This model is described by the transitions $P_1 \underset{k_1^-}{\overset{k_1^+}{\rightleftharpoons}} P_2 \underset{k_2^-}{\overset{k_2^+}{\rightleftharpoons}} P_4 \underset{k_3^+}{\overset{k_3^-}{\rightleftharpoons}} P_3$ (with $\xrightarrow{k_4}$ leaving P_4 upward). In this case P_4 is the ON state. The model is of type II.

We have

$$\tilde{Q}=\begin{bmatrix} -k_1^+ & k_1^- & 0 & 0 \\ k_1^+ & -(k_2^+ + k_1^-) & 0 & k_2^- \\ 0 & 0 & k_3^+ k_3^- & \\ 0 & k_2^+ & k_3^+ & -(k_4+k_3^-+k_2^-) \end{bmatrix}.$$

The Vieta formulas read

$$k_4k_1^+k_2^+k_3^+ = L_4 \tag{95}$$

$$k_4k_1^-k_3^+ + k_4k_1^+k_2^+ + k_4k_1^+k_3^+ + k_4k_2^+k_3^+ + k_1^-k_2^-k_3^+ + k_2^-k_1^+k_3^+ + k_3^-k_1^+k_2^+ + \\ +k_1^+k_2^+k_3^+ = -L_3 \tag{96}$$

$$k_4k_1^- + k_4k_1^+ + k_4k_2^+ + k_4k_3^+ + k_1^-k_2^- + k_1^-k_3^- + k_2^-k_1^+ + k_1^-k_3^+ + k_3^-k_1^+ + k_2^-k_3^+ + k_3^-k_2^+ + \\ +k_1^+k_2^+ + k_1^+k_3^+ + k_2^+k_3^+ = L_2 \tag{97}$$

$$k_4 + k_1^- + k_2^- + k_3^- + k_1^+ + k_2^+ + k_3^+ = -L_1 \tag{98}$$

The eigenvectors of $\tilde{Q}$ are

$$u_1 = \frac{k_1^-k_2^-}{k_1^+k_2^+ + k_1^-\lambda + k_1^+\lambda + k_2^+\lambda + \lambda^2} \tag{99}$$

$$u_2 = \frac{k_2^-(k_1^+ + \lambda)}{k_1^+k_2^+ + k_1^-\lambda + k_1^+\lambda + k_2^+\lambda + \lambda^2} \tag{100}$$

$$u_3 = \frac{k_3^-}{k_3^+ + \lambda} \tag{101}$$

$$u_4 = 1 \tag{102}$$

The system (30) has the solution

$$
\begin{aligned}
C_1 &= -\frac{k_1^- \lambda_1^2 + k_1^+ \lambda_1^2 + k_2^+ \lambda_1^2 + k_3^+ \lambda_1^2 + \lambda_1^3 + k_1^+ k_2^+ k_3^+ + k_1^- k_3^+ \lambda_1 + k_1^+ k_2^+ \lambda_1 + k_1^+ k_3^+ \lambda_1 + k_2^+ k_3^+ \lambda_1}{(\lambda_1-\lambda_2)(\lambda_1\lambda_3+\lambda_1\lambda_4-\lambda_3\lambda_4-\lambda_1^2)} \\
C_2 &= -\frac{(k_3^+ + \lambda_2)(k_1^+ k_2^+ + k_1^- \lambda_2 + k_1^+ \lambda_2 + k_2^+ \lambda_2 + \lambda_2^2)}{(\lambda_1-\lambda_2)(\lambda_2-\lambda_3)(\lambda_2-\lambda_4)} \\
C_3 &= \frac{(k_3^+ + \lambda_3)(k_1^+ k_2^+ + k_1^- \lambda_3 + k_1^+ \lambda_3 + k_2^+ \lambda_3 + \lambda_3^2)}{(\lambda_3-\lambda_4)(\lambda_1\lambda_2-\lambda_1\lambda_3-\lambda_2\lambda_3+\lambda_3^2)} \\
C_4 &= -\frac{(k_3^+ + \lambda_4)(k_1^+ k_2^+ + k_1^- \lambda_4 + k_1^+ \lambda_4 + k_2^+ \lambda_4 + \lambda_4^2)}{\lambda_1\lambda_4^2+\lambda_2\lambda_4^2+\lambda_3\lambda_4^2-\lambda_4^3+\lambda_1\lambda_2\lambda_3-\lambda_1\lambda_2\lambda_4-\lambda_1\lambda_3\lambda_4-\lambda_2\lambda_3\lambda_4} \qquad (103)
\end{aligned}
$$

The eqs. (29) and (31) provide

$$k_4 = -S_1$$

$$k_3^- = \frac{-S_1(k_3^+)^3 - S_1(L_1-S_1)(k_3^+)^2 - S_1(L_2+S_2-L_1S_1)k_3^+ - S_1(L_3-S_3+L_1S_2-L_2S_1)}{3S_1(k_3^+)^2+(2L_1S_1-2S_2)k_3^+ + S_3 - L_1S_2 + L_2S_1},$$

$k_2^+ = (S_1^3(k_3^+)^4 + (-S_1^4 + L_1S_1^3)(k_3^+)^3 + (-L_1S_1^4 + S_1^3S_2 + L_2S_1^3 + 3S_3S_1^2 - 3S_1S_2^2)(k_3^+)^2 + (L_3S_1^3 - L_2S_1^4 -$
$S_1^3S_3 + 2S_2^3 - 2S_1S_2S_3 - 2L_1S_1S_2^2 + 2L_1S_1^2S_3 + L_1S_1^3S_2)k_3^+ + L_2S_1^2S_3 - L_2S_1S_2^2 - L_1S_1S_2S_3 + S_1S_3^2 + L_1S_2^3 -$
$S_2^2S_3)/(S_1^3(k_3^+)^3 + (S_1(2S_1^3 + L_1S_1^2 - 3S_2S_1))(k_3^+)^2 + (S_1(L_1S_1^3 - S_1^2S_2 + L_2S_1^2 - 2L_1S_1S_2 + 2S_2^2))k_3^+ - S_1(-L_3S_1^2 +$
$L_2S_1S_2 - L_1S_2^2 + S_3S_2)),$

$k_2^- = (S_1^2(k_3^+)^3 + (2S_1^3 + L_1S_1^2 - 3S_2S_1)(k_3^+)^2 + (L_1S_1^3 - S_1^2S_2 + L_2S_1^2 - 2L_1S_1S_2 + 2S_2^2)k_3^+ + L_3S_1^2 - L_2S_1S_2 +$
$L_1S_2^2 - S_3S_2)/((3S_1^2)(k_3^+)^2 + (-S_1(2S_2 - 2L_1S_1))k_3^+ + S_1(S_3 - L_1S_2 + L_2S_1)),$

$k_1^+ = (S_1^3(k_3^+)^5 + (S_1(2S_1^3 + L_1S_1^2 - 3S_2S_1) - S_1^2(S_2 - L_1S_1))(k_3^+)^4 + (S_1(L_1S_1^3 - S_1^2S_2 + L_2S_1^2 - 2L_1S_1S_2 +$
$2S_2^2) + S_1^2(S_3 - L_1S_2 + L_2S_1) - (S_2 - L_1S_1)(2S_1^3 + L_1S_1^2 - 3S_2S_1))(k_3^+)^3 + ((S_3 - L_1S_2 + L_2S_1)(2S_1^3 + L_1S_1^2 -$
$3S_2S_1) - S_1(-L_3S_1^2 + L_2S_1S_2 - L_1S_2^2 + S_3S_2) - (S_2 - L_1S_1)(L_1S_1^3 - S_1^2S_2 + L_2S_1^2 - 2L_1S_1S_2 + 2S_2^2))(k_3^+)^2 +$
$((S_2 - L_1S_1)(-L_3S_1^2 + L_2S_1S_2 - L_1S_2^2 + S_3S_2) + (S_3 - L_1S_2 + L_2S_1)(L_1S_1^3 - S_1^2S_2 + L_2S_1^2 - 2L_1S_1S_2 + 2S_2^2))k_3^+ -$
$(S_3 - L_1S_2 + L_2S_1)(-L_3S_1^2 + L_2S_1S_2 - L_1S_2^2 + S_3S_2)/(S_1^3(k_3^+)^4 + (-S_1^4 + L_1S_1^3)(k_3^+)^3 + (-L_1S_1^4 + S_1^3S_2 + L_2S_1^3 +$
$3S_3S_1^2 - 3S_1S_2^2)(k_3^+)^2 + (L_3S_1^3 - L_2S_1^4 - S_1^3S_3 + 2S_2^3 - 2S_1S_2S_3 - 2L_1S_1S_2^2 + 2L_1S_1^2S_3 + L_1S_1^3S_2)k_3^+ + L_2S_1^2S_3 -$
$L_2S_1S_2^2 - L_1S_1S_2S_3 + S_1S_3^2 + L_1S_2^3 - S_2^2S_3),$

$k_1^- = -(S_1(S_3 - 2S_2k_3^+ + 3S_1(k_3^+)^2 - L_1S_2 + L_2S_1 + 2L_1S_1k_3^+)(L_1^2S_1^4(k_3^+)^3 - L_1^2S_1^3S_2(k_3^+)^2 + L_1^2S_1^3S_3k_3^+ +$
$L_1^2S_1^3(k_3^+)^4 - 3L_1^2S_1^2S_2(k_3^+)^3 + L_1^2S_1^2S_3(k_3^+)^2 + 3L_1^2S_1S_2^2(k_3^+)^2 - 2L_1^2S_1S_2S_3k_3^+ - L_1^2S_2^3k_3^+ + L_1^2S_2^2S_3 + L_1L_2S_1^4(k_3^+)^2 -$
$L_1L_2S_1^3S_2k_3^+ + 2L_1L_2S_1^3(k_3^+)^3 - 5L_1L_2S_1^2S_2(k_3^+)^2 + L_1L_2S_1^2S_3k_3^+ + 4L_1L_2S_1S_2^2k_3^+ - L_1L_2S_1S_2S_3 - L_1L_2S_2^3 +$
$L_1L_3S_1^4k_3^+ + 2L_1L_3S_1^3(k_3^+)^2 - 3L_1L_3S_1^2S_2k_3^+ + L_1L_3S_1^2S_3 + L_1L_3S_1S_2^2 + L_1S_1^5(k_3^+)^3 - L_1S_1^4S_2(k_3^+)^2 + 2L_1S_1^4(k_3^+)^4 -$

$5L_1S_1^3S_2(k_3^+)^3+L_1S_1^3S_3(k_3^+)^2+2L_1S_1^3(k_3^+)^5+4L_1S_1^2S_2^2(k_3^+)^2-L_1S_1^2S_2S_3k_3^+-6L_1S_1^2S_2(k_3^+)^4+4L_1S_1^2S_3(k_3^+)^3-L_1S_1S_2^3k_3^++6L_1S_1S_2^2(k_3^+)^3-8L_1S_1S_2S_3(k_3^+)^2+2L_1S_1S_3^2k_3^+-2L_1S_2^3(k_3^+)^2+4L_1S_2^2S_3k_3^+-2L_1S_2S_3^2+L_2^2S_1^3(k_3^+)^2-2L_2^2S_1^2S_2k_3^++L_2^2S_1S_2^2+2L_2L_3S_1^3k_3^+-2L_2L_3S_1^2S_2+L_2S_1^5(k_3^+)^2+L_2S_1^4(k_3^+)^3-4L_2S_1^3S_2(k_3^+)^2-2L_2S_1^3S_3k_3^++2L_2S_1^3(k_3^+)^4+3L_2S_1^2S_2^2k_3^+-5L_2S_1^2S_2(k_3^+)^3+3L_2S_1^2S_3(k_3^+)^2+4L_2S_1S_2^2(k_3^+)^2-4L_2S_1S_2S_3k_3^++L_2S_1S_3^2-L_2S_2^3k_3^++L_2S_2^2S_3+L_3^2S_1^3+L_3S_1^4(k_3^+)^2-L_3S_1^3S_2k_3^++2L_3S_1^3(k_3^+)^3-3L_3S_1^2S_2(k_3^+)^2+3L_3S_1^2S_3k_3^+-3L_3S_1S_2S_3+L_3S_2^3+S_1^5(k_3^+)^4-S_1^4S_2(k_3^+)^3+S_1^4S_3(k_3^+)^2+S_1^4(k_3^+)^5-4S_1^3S_2(k_3^+)^4+S_1^3(k_3^+)^6+4S_1^2S_2^2(k_3^+)^3-4S_1^2S_2S_3(k_3^+)^2-3S_1^2S_2(k_3^+)^5-2S_1^2S_3^2k_3^++3S_1^2S_3(k_3^+)^4+4S_1S_2^2S_3k_3^++3S_1S_2^2(k_3^+)^4-6S_1S_2S_3(k_3^+)^3+3S_1S_3^2(k_3^+)^2-S_2^4k_3^+-S_2^3(k_3^+)^3+3S_2^2S_3(k_3^+)^2-3S_2S_3^2k_3^++S_3^3))/((2S_1^3(k_3^+)^2+L_1S_1^3k_3^+-S_1^2S_2k_3^++S_1^2(k_3^+)^3+L_1S_1^2(k_3^+)^2+L_2S_1^2k_3^++L_3S_1^2-3S_1S_2(k_3^+)^2-2L_1S_1S_2k_3^+-L_2S_1S_2+2S_2^2k_3^++L_1S_2^2-S_3S_2)(S_1^3S_2(k_3^+)^2-L_1S_1^4(k_3^+)^2-L_2S_1^4k_3^+-S_1^4(k_3^+)^3+L_1S_1^3S_2k_3^+-S_1^3S_3k_3^++S_1^3(k_3^+)^4+L_1S_1^3(k_3^+)^3+L_2S_1^3(k_3^+)^2+L_3S_1^3k_3^++3S_1^2S_3(k_3^+)^2+2L_1S_1^2S_3k_3^++L_2S_1^2S_3-3S_1S_2^2(k_3^+)^2-2L_1S_1S_2^2k_3^+-L_2S_1S_2^2-2S_1S_2S_3k_3^+-L_1S_1S_2S_3+S_1S_3^2+2S_2^3k_3^++L_1S_2^3-S_2^2S_3))$,

where k_3^+ is the solution of the cubic equation

$$S_1(k_3^+)^3+(L_1S_1-S_2)(k_3^+)^2+(L_2S_1-L_1S_2+S_3)k_3^+L_4=0. \tag{104}$$

The equation (104) has the discriminant

$$\begin{aligned}\mathit{\Delta}=&(S_2-L_1S_1)^2(S_3-L_1S_2+L_2S_1)^2+4L_4(S_2-L_1S_1)^3\\&-27L_4^2S_1^2-4S_1(S_3-L_1S_2+L_2S_1)^3-18L_4S_1(S_2-L_1S_1)(S_3-L_1S_2+L_2S_1).\end{aligned} \tag{105}$$

When $\mathit{\Delta}<0$, there is an unique real solution

$$k_3^+=\frac{S_2-L_1S_1}{3S_1}+(\mathit{\Delta}_3)^{1/3}+\frac{\frac{(S_2-L_1S_1)^2}{9S_1^2}-\frac{S_3-L_1S_2+L_2S_1}{3S_1}}{\mathit{\Delta}_3^{1/3}}, \tag{106}$$

where $\mathit{\Delta}_3=\frac{(S_2-L_1S_1)^3}{27S1^3}-\frac{L_4}{2S_1}-\frac{(S_2-L_1S_1)(S_3-L_1S_2+L_2S_1)}{6S1^2}+\frac{\sqrt{-\mathit{\Delta}/108}}{S_1^2}$.

4.10 Symbolic solution to the inverse problem for four state chain with return in state P_3 ($N=4$)

This model has exactly the same transitions as the 4 state chain model described in the Section 4.8 with the difference that the ON state is P_3.

Using the same methods as in Section 4.6 we show that in this case

$$A_1\lambda_1+A_2\lambda_2+A_3\lambda_3+A_4\lambda_4=0. \tag{107}$$

Using (107) and $A_1 + A_2 + A_3 + A_4 = 1$ we can compute the remaining parameters as

$$\begin{aligned} A_3 &= -\frac{\lambda_4 + A_1(\lambda_1 - \lambda_4) + A_2(\lambda_2 - \lambda_4)}{\lambda_3 - \lambda_4}, \\ A_4 &= \frac{\lambda_3 + A_1(\lambda_1 - \lambda_3) + A_2(\lambda_2 - \lambda_3)}{\lambda_3 - \lambda_4}. \end{aligned} \tag{108}$$

If the condition (107) is not satisfied, then there is no solution to the inverse problem.

If the condition (107) is satisfied, then the inverse problem is not well posed and has an infinity of solutions. In this case, all the solutions can be expressed as functions of a free parameter. In the sequel we will choose k_4 as free parameter. Although we were able to obtain analytic solutions, these are too long to be displayed.

The following, simple relations are useful for the anaysis of this model:

$$\begin{aligned} k_3^+ &= -S_2/k_4, \\ k_3^- + k_2^- &= -S_3/S_2 + S_2/k_4 - k_4 \end{aligned} \tag{109}$$

5 Uncertainty estimation for the model parameters

In Section 3.3 we have used optimization with multiple initial parameters to estimate confidence intervals for each parameter of the multi-exponential survival function as lower and upper bounds of optimal and sub-optimal parameters. These intervals are presented as $A_i \in [A_i^{min}, A_i^{max}]$, $1 \leq i \leq N$ and $\lambda_i \in [\lambda_i^{min}, \lambda_i^{max}]$, $1 \leq i \leq N$.

In the sections above we have shown how to compute symbolically the kinetic parameters of various models from the parameters A_i, λ_i, $1 \leq i \leq N$ of the multi-exponential survival function. By applying the symbolic mapping to the confidence intervals $[A_i^{min}, A_i^{max}]$,$[\lambda_i^{min}, \lambda_i^{max}]$ one can get the confidence intervals of the kinetic parameters. However, finding intervals that bound the kinetic parameters from the confidence intervals of the survival function parameters is a non-convex optimization problem with constraints which may prove difficult. Therefore, in the current implementation of our software we decided to apply the symbolic mapping directly to the entire set of optimal and sub-optimal survival function parameters obtained in Section 3.3 and compute the lower and upper bounds of the resulting kinetic parameters.

6 Computing the probability of each state at stationarity

Although the computation of the distribution of waiting times does not require stationarity conditions (successive waiting times form a renewal process even without stationarity, as soon and as long as the model parameters

are constant in time) it is usefull to have estimates for the stationary probabilities of being in each of the model's state. The sojourn time in the state P_{N+1} being nil the probability of being in this state is also nil. The remaining N probabilities $p_i = \mathbb{P}[M(t) = P_i]\,, 1 \leq i \leq N$ satisfy $p_1 + p_2 + \ldots + p_N = 1$ and the following homogeneous system of linear equations:

$$\tilde{\tilde{\boldsymbol{Q}}} \begin{pmatrix} p_1 \\ p_2 \\ \vdots \\ p_N \end{pmatrix} = 0, \tag{110}$$

where $\tilde{\tilde{\boldsymbol{Q}}}$ is obtained from $\tilde{\boldsymbol{Q}}$ by setting $k_{N,N+1} = 0$.

A few examples follow.

For the model M1,

$$\tilde{\tilde{\boldsymbol{Q}}} = \begin{bmatrix} -k_1^+ & k_1^- & 0 \\ k_1^+ & -(k_2^+ + k_1^-) & k_2^- \\ 0 & k_2^+ & -k_2^- \end{bmatrix},$$

$$p_1 = \frac{k_1^- k_2^-}{k_1^+ k_2^- + k_1^- k_2^- + k_1^+ k_2^+}, \tag{111}$$

$$p_2 = \frac{k_1^+ k_2^-}{k_1^+ k_2^- + k_1^- k_2^- + k_1^+ k_2^+}, \tag{112}$$

$$p_3 = \frac{k_1^+ k_2^+}{k_1^+ k_2^- + k_1^- k_2^- + k_1^+ k_2^+}. \tag{113}$$

For the model M2,

$$\tilde{\tilde{\boldsymbol{Q}}} = \begin{bmatrix} -k_1^+ & 0 & k_1^- \\ 0 & -k_2^+ & k_2^- \\ k_1^+ & k_2^+ & -(k_1^- + k_2^-) \end{bmatrix},$$

$$p_1 = \frac{k_1^- k_2^+}{k_1^+ k_2^+ + k_1^- k_2^+ + k_1^+ k_2^-}, \tag{114}$$

$$p_2 = \frac{k_1^+ k_2^-}{k_1^+ k_2^+ + k_1^- k_2^+ + k_1^+ k_2^-}, \tag{115}$$

$$p_3 = \frac{k_1^+ k_2^+}{k_1^+ k_2^+ + k_1^- k_2^+ + k_1^+ k_2^-}. \tag{116}$$

For the four states model,

$$\tilde{\tilde{\boldsymbol{Q}}} = \begin{bmatrix} -k_1^+ & k_1^- & 0 & 0 \\ k_1^+ & -(k_2^+ + k_1^-) & k_2^- & 0 \\ 0 & k_2^+ & -(k_3^+ + k_2^-) & k_3^- \\ 0 & 0 & k_3^+ & -k_3^- \end{bmatrix},$$

$$p_1 = \frac{k_1^- k_2^- k_3^-}{k_1^+ k_2^+ k_3^+ + k_1^+ k_2^+ k_3^- + k_1^+ k_2^- k_3^- + k_1^- k_2^- k_3^-}, \quad (117)$$

$$p_2 = \frac{k_1^+ k_2^- k_3^-}{k_1^+ k_2^+ k_3^+ + k_1^+ k_2^+ k_3^- + k_1^+ k_2^- k_3^- + k_1^- k_2^- k_3^-}, \quad (118)$$

$$p_3 = \frac{k_1^+ k_2^+ k_3^-}{k_1^+ k_2^+ k_3^+ + k_1^+ k_2^+ k_3^- + k_1^+ k_2^- k_3^- + k_1^- k_2^- k_3^-}, \quad (119)$$

$$p_4 = \frac{k_1^+ k_2^+ k_3^+}{k_1^+ k_2^+ k_3^+ + k_1^+ k_2^+ k_3^- + k_1^+ k_2^- k_3^- + k_1^- k_2^- k_3^-}. \quad (120)$$

For the two states (ON-OFF) model

$$\tilde{\tilde{\boldsymbol{Q}}} = \begin{bmatrix} -k_1^+ & k_1^- \\ k_1^+ & -k_1^- \end{bmatrix},$$

$$p_1 = \frac{k_1^-}{k_1^+ + k_1^-}, \quad (121)$$

$$p_2 = \frac{k_1^+}{k_1^+ + k_1^-}. \quad (122)$$

7 Computing the mean mRNA at the steady state

The statistics of the waiting time between two successive transcription initiations can be used to compute the statistics of the number of mRNA molecules. Each elongating polymerase will generate one molecule of mRNA that will survive in the average a time $T \approx 45\,\text{min}$. Therefore the mean mRNA number at the steady state is simply:

$$mRNA = 45/w, \quad (123)$$

where w is the average waiting time.

An equivalent formula is

$$mRNA = 45 p_{ON} k_{ini}, \quad (124)$$

where p_{ON}, k_{ini} are the probability and the initiation rate of the ON state, respectively.

The straightforward calculation

$$w = -\int_0^\infty tS'(t)\,dt = \int_0^\infty S(t)\,dt = -\sum_{i=1}^{N} \frac{A_i}{\lambda_i},$$

and the Eq.123 lead to two equivalent ways to compute the mean mRNA number, from the area under curve, or from the parameters of the survival function

$$mRNA = 45/\mathrm{AUCS} = -45/\sum_{i=1}^{N} \frac{A_i}{\lambda_i}, \tag{125}$$

where AUCS is the area under curve of the survival function.

The mean mRNA number can also be related to the area under curve of the MS2 signal, that we call integral amplitude. Let AUCP be the area under curve of the time dependent signal produced by a single polymerase (in our data AUCP $= 2.425$ min) and T the length of the signal. Then the integral amplitude IA satisfies IA $= \mathrm{AUCP} \times T/w$. Using (123), it follows:

$$mRNA = \frac{45}{\mathrm{AUCP}} \times \frac{\mathrm{IA}}{T} = 18.55\frac{\mathrm{IA}}{T}. \tag{126}$$

The Eq.124 leads to the formulas:

$$mRNA = 45\frac{k_1^+ k_2}{k_1^+ + k_1^-}, \tag{127}$$

for the two state ON-OFF model,

$$mRNA = 45\frac{k_1^+ k_2^+ k_3}{k_1^- k_2^+ + k_2^- k_1^+ + k_1^+ k_2^+}, \tag{128}$$

for the 3 state, $M2$ model,

$$mRNA = 45\frac{k_1^+ k_2^+ k_3}{k_1^+ k_2^- + k_1^- k_2^- + k_1^+ k_2^+}, \tag{129}$$

for the 3 state, $M1$ model, and

$$mRNA = 45\frac{k_1^+ k_2^+ k_3^+ k_4}{k_1^+ k_2^+ k_3^+ + k_1^+ k_2^+ k_3^- + k_1^- k_2^- k_3^-}, \tag{130}$$

for the 4 state model.

Data set	b_0	b_1	b_2	b_3
Low tat	0.27	0.026	0.0022	-1.5e-5
No tat	-0.97	0.23	-0.0021	9.8e-6
High tat	0.27	0.026	0.0022	-1.5e-5

Table S2: Noise parameters for various experimental conditions in the study of the HIV-1 promoter.

8 Testing the robustness of the method using artificial data

The numerical method is based on the assumption that the instrumental noise and other sources of noise are averaged out by the algorithm and therefore can be neglected. In this subsection we use artificial data to test the consequences of releasing this assumption. Furthermore, the optimization algorithm is stochastic and include approximate steps such as the estimation of the parameters p_s and p_l, and errors resulting from the analog to digital conversion of the long movie signals. Artificially generated data with well know parameters will also allow us to test the fidelity of the parameter identification in our method.

Artificial data was generated by simulating the model M1 using the Gillespie algorithm. We use three parameter sets, similar to those identified from data in the three experimental conditions (previous subsection). The simulations generate artificial polymerase positions from which we first compute a noiseless signal using Eq. (1).

In a second step we add to the signal a centered Gaussian noise, whose variance is similar to the one in data, as follows

$$S_\eta(t) = S(t) + \eta(t), \quad (131)$$

where $S(t)$ is the noiseless signal and $\eta(t)$ is the noise.

The noise estimate is obtained from the short movies data. It is defined as the difference between the raw signal and the signal reconstructed by deconvolution (computed using Eq. (1)). We found that the noise variance is an increasing function of the signal amplitude. By using cubic polynomial interpolation we have derived analytic formulas for the variance in the three experimental conditions:

$$Var(\eta) = b_3 S^3 + b_2 S^2 + b_1 S + b_0, \quad (132)$$

where b_i, $0 \le i \le 3$ are parameters whose values can be found in the Table S2.

We applied our algorithm to a raw signal described by (131) and obtained estimates of the kinetic parameters. η is defined by (132) and Table S2. Together with η we have also tested the double 2η and four times 4η noise amplitude. These estimates were compared to the know values of the parameters that were used for simulating the artificial data. The result of the comparison is shown in Fig.S17.

The method faithfully retrieves the parameter values, at least for noise amplitudes comparable to the ones determined from the data used in this study. For larger noise amplitudes some parameters may not be faithfully retrieved. As expected, some large kinetic parameters, corresponding to small time scales are not faithfully retrieved. However, the small parameters, corresponding to large time scales are faithfully retrieved even for large noise amplitudes. This proves the robustness of the method with respect to noise.

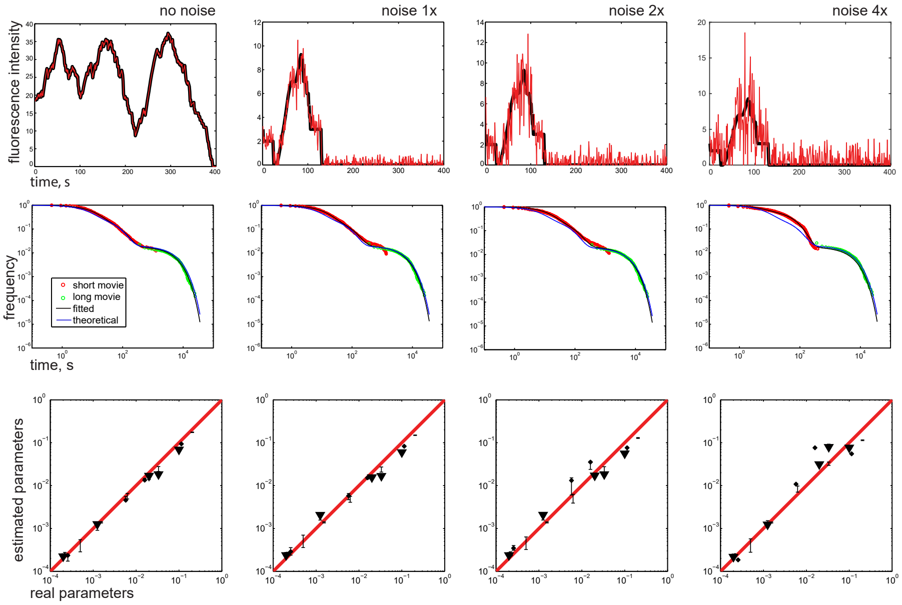

Figure S17: Testing the algorithm with artificial data for various noise amplitude. $\times 1$ represents artificial data with the same amplitude of noise as the real data. $\times 0$ is the noiseless artificial data. For each comparison we consider 3 sets of 5 parameters corresponding to the three experimental conditions in the HIV-1 data: no tat, low tat and high tat. The survival functions (middle row) and the artificial signal (upper row) are shown only for the no-tat conditions.

9 Results.

9.1 Identifying the parameters of the model M2.

Model M2 corresponds to the stochastic, facultative pausing (Figure S5). The model parameters can be identified from a unconstrained three-exponential fit (Figure S16).

The results of the fit are presented in the Table S3 and in the Figure S18.

Type	OBJ	mRNA	k_1^+	k_1^-	k_2^+	k_2^-	k_3
no tat optimal	0.028	16.7	6.0e-05	0.00035	0.00089	0.003	0.063
min		16.7	6.0e-05	0.00021	0.00089	0.00199	0.06
max		29.5	7.1e-05	0.00035	0.00130	0.003	0.063
low tat optimal	0.061	49.6	0.00015	0.00031	0.0012	0.0028	0.1
min		49.6	0.00015	0.00021	0.0012	0.0021	0.099
max		114	0.00022	0.00100	0.028	0.0180	0.15
high tat optimal	0.115	315	0.0015	4.9e-05	0.0100	0.0043	0.17
min		265	0.0014	4.9e-05	0.0052	0.003	0.16
max		315	0.0015	6.3e-05	0.0100	0.0043	0.17

Table S3: Results of the unconstrained three-exponential fit of the model M2. $\alpha = 0.30$

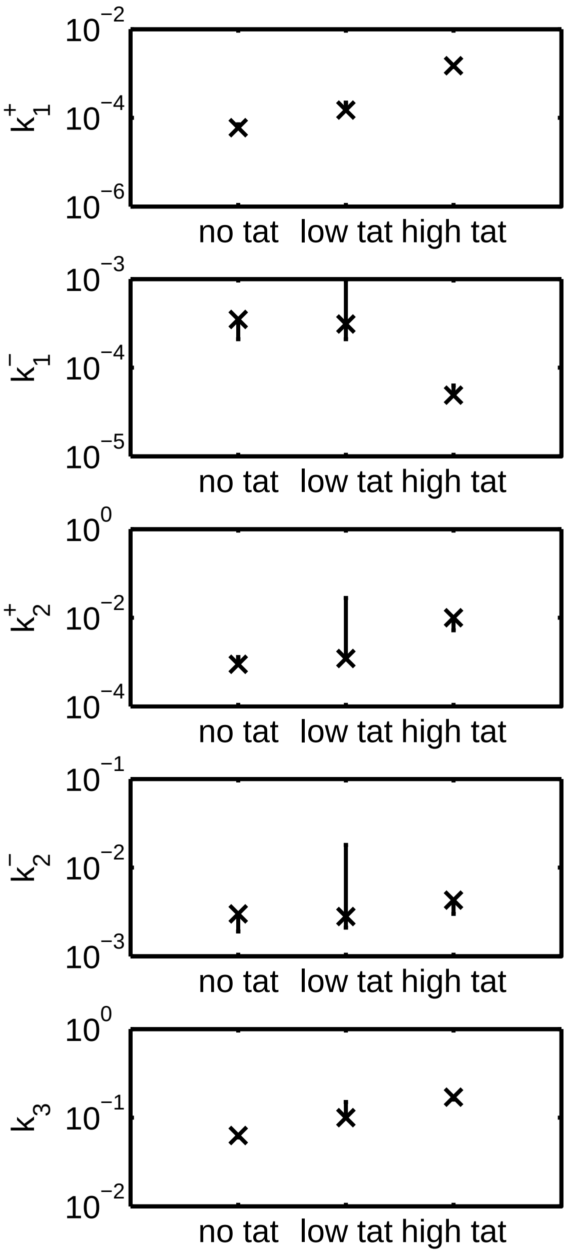

Figure S18: Results of the unconstrained three-exponential fit of the model M2. Parameter dependence on the experimental conditions for $\alpha = 0.30$. The vertical bars are uncertainty intervals.

9.2 Identifying the parameters of the two states ON-OFF model.

In order to identify this model we use a two exponential fit of the survival function $S(t) = A_1 \exp(\lambda_1 t) + A_2 \exp(\lambda_2 t)$. The model parameters are computed from the survival function parameters according to the Section 4.7.

The result of the fit is given in the Table S4 and in the Figure S19. The large values of the objective function suggest that this model is not suitable for our data.

Type	OBJ	λ_1	λ_2	A_1	A_2	$k2$	k_1^-	k_1^+	mRNA
no tat optimal	0.22046	-0.0478	-0.000141	0.984	0.0159	0.047	0.000755	0.000144	20.3
min		-0.0478	-0.000141	0.984	0.00646	0.0444	0.000287	0.000136	
max		-0.0446	-0.000135	0.994	0.0159	0.047	0.000755	0.000144	
low tat optimal	0.273	-0.0782	-0.00025	0.991	0.00943	0.0774	0.000733	0.000252	53.5
min		-0.0782	-0.00025	0.991	0.00368	0.0719	0.000264	0.000244	
max		-0.0721	-0.000243	0.996	0.00943	0.0774	0.000733	0.000252	
high tat optimal	0.64799	-0.12	-0.00222	0.996	0.00422	0.12	0.000488	0.00223	264.8
min		-0.12	-0.00222	0.00278	0.00113	0.097	0.000105	0.00218	
max		-0.0971	-0.00218	0.999	0.997	0.12	0.000488	0.00223	

Table S4: Results of the two-exponential fit, $\alpha = 0.30$. The objective function has large values compared to the three state model M_2, for the same value of α.

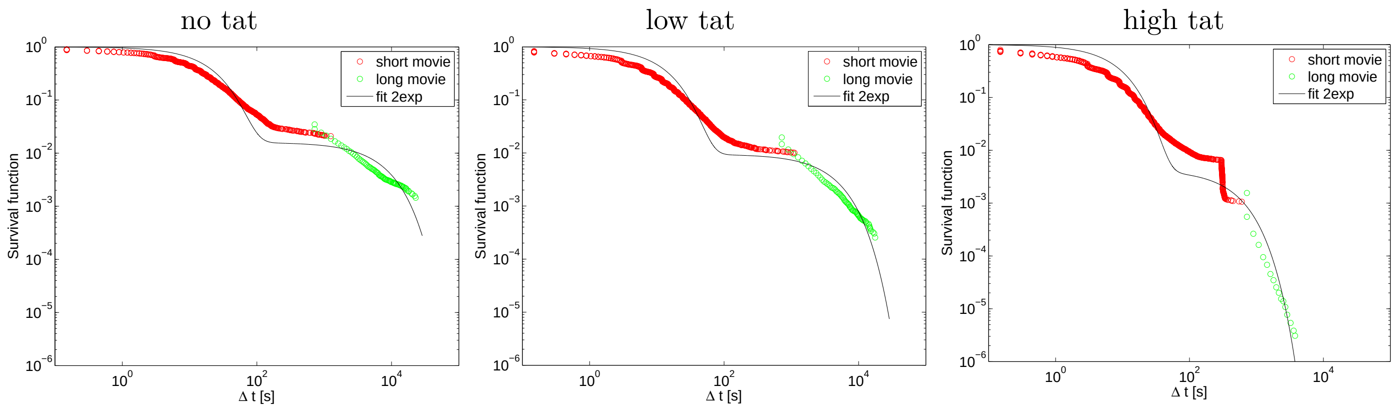

Figure S19: Results of the two-exponential fit: most optimal fit for $\alpha = 0.30$.

9.3 Identifying the parameters of the model M3

Model M3 corresponds to obligatory pausing (see Figure S5) and is identified using the constrained three-exponential fit described in the Section 4.6. However, even if (up to errors) the three-exponential fit provides a single best fit, the set of corresponding parameters of model M3 is a curve in the 5D space of parameters. The inverse problem for model M3 is not well posed as the relation between the parameters of the model M3 and the parameters of the three-exponential fit is many to one. The result of the constrained three-exponential fit is given in the Table S5.

The dependence of the parameters of the model M3 on the undetermined parameter k_3 is shown in the Figure S21. The parameters $k_2^{\pm}$ have very large values compared to all other parameters. The model $M3$ is in this case equivalent to the two states ON-OFF model and inherits the difficulty of this model to fit the data.

Type	OBJ	λ_1	λ_2	λ_3	A_1	A_2	A_3	mRNA
no tat optimal	0.22	-5660	-0.0478	-0.000141	-8.31e-06	0.984	0.0159	20.3
min		-7380	-0.0708	-0.000169	-0.00232	0.984	0.0060	
max		-2	-0.029	-0.000121	-6e-6	1.02	0.0161	
low tat optimal	0.27	-8480	-0.0782	-0.00025	-9.13e-06	0.991	0.00943	53.5
min		-12500	-0.148	-0.000278	-68.3	0.99	0.00306	
max		-0.144	-0.0556	-0.000224	-5.27e-06	69.3	0.0125	
high tat optimal	0.65	-26000	-0.12	-0.00222	-4.59e-06	0.996	0.00422	264.8
min		-54200	-0.166	-0.00239	-1.49	0.994	0.000797	
max		-0.254	-0.0692	-0.00202	-1.74e-06	2.48	0.00647	

Table S5: Results of the constrained three-exponential fit of the model M3, $\alpha = 0.30$. The objective function has large values (compared to different models and for the same α) and the fitted parameters are very uncertain.

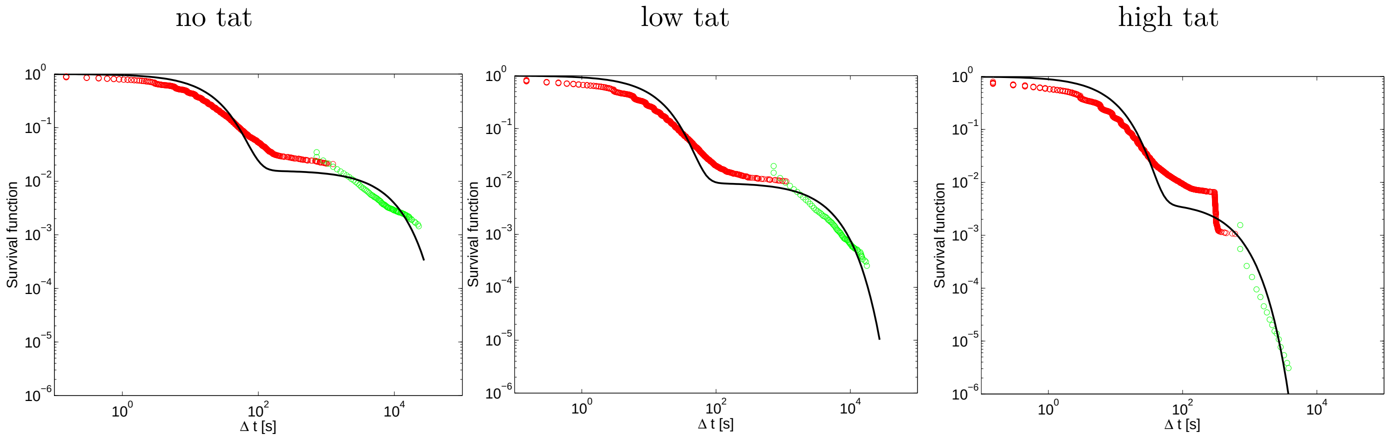

Figure S20: Results of the constrained three-exponential fit: most optimal fit for $\alpha = 0.30$.

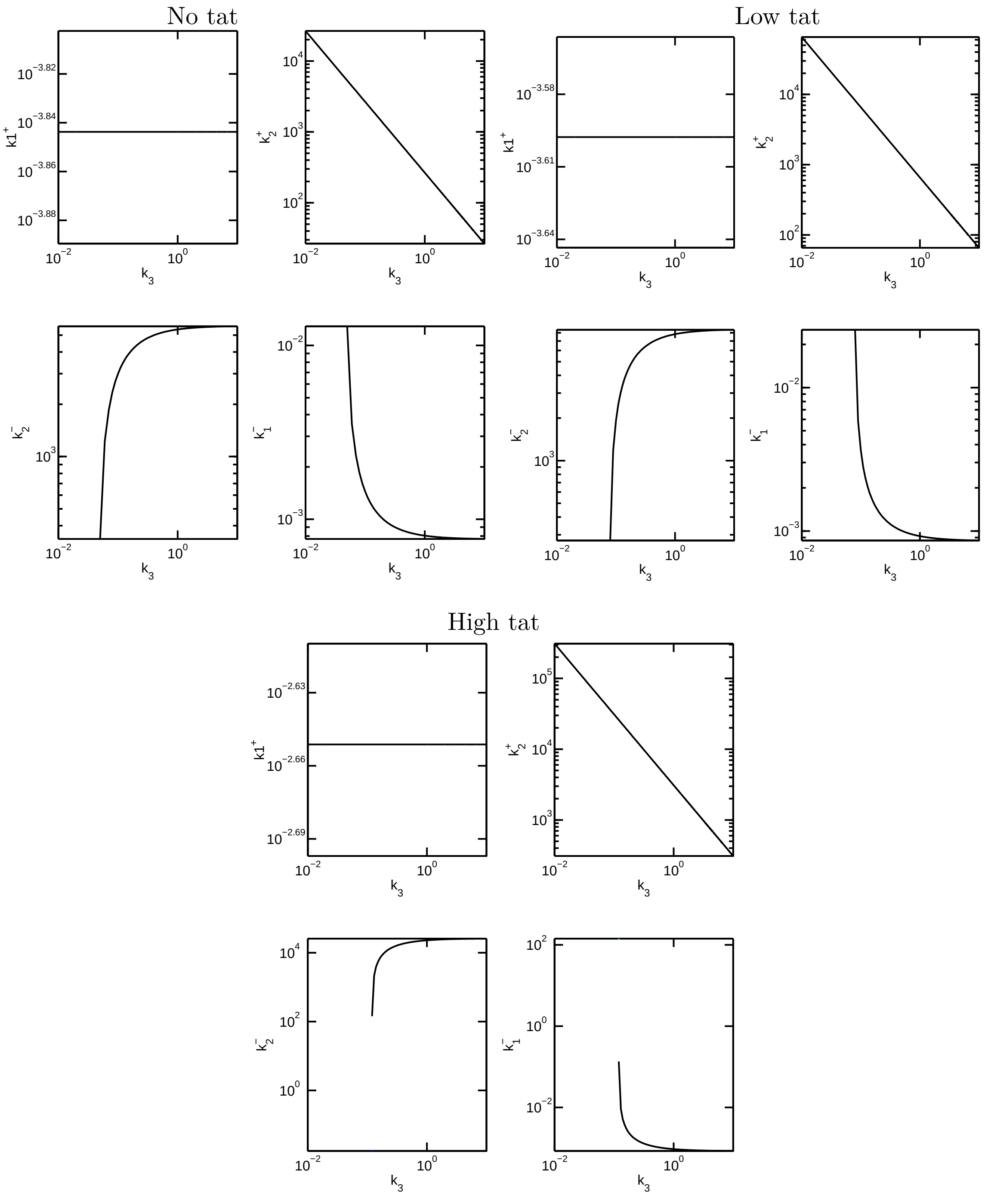

Figure S21: Results of the constrained three-exponential fit of the model M3. Parameter dependence on the undetermined parameter k_3 (pause exit rate) for $\alpha = 0.30$. The parameters $k_2^{\pm}$ have very large values compared to all other parameters and correspond to very fast processes (timescales smaller than $0.01s$). For such parameters the model $M3$ is equivalent to a two states ON-OFF model (the states ON and PAUSE can be pooled with no information loss in the model M3). In order to ensure positivity of kinetic parameters, one needs $k_3 > 0.1s^{-1}$.

9.4 Identifying the parameters of a four states model with pausing.

In order to identify four states models we use a four exponential fit of the survival function $S(t) = A_1 \exp(\lambda_1 t) + A_2 \exp(\lambda_2 t) + A_3 \exp(\lambda_3 t) + A_4 \exp(\lambda_4 t)$, where $A_1 + A_2 + A_3 + A_4 = 1$. Let us consider that $\lambda_1 < \lambda_2 < \lambda_3 < \lambda_4 < 0$. From $S'(t) \leq 0$ it follows $\lambda_1 A_1 + \lambda_2 A_2 + \lambda_3 A_3 + \lambda_4 A_4 \leq 0$, $A_4 \geq 0$.

The model M_4 is obtained by adding one more OFF state to the model M_3 (see Figure S22). It corresponds to the theoretical model described in the Section 4.10. The parameters of this model can be obtained from a constrained four exponential fit with six free parameters λ_1,λ_2,λ_3,λ_4,A_1,A_2 (see Eq.(108)). Although this model has more free parameters than the model M_2, the fit quality is lower.

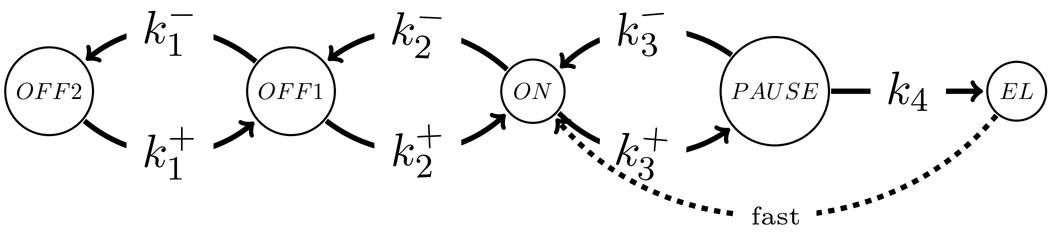

Figure S22: Model M4 with two OFF states and obligatory pausing. k_4 is the pause exit rate, k_3^- is the transcription abortion rate.

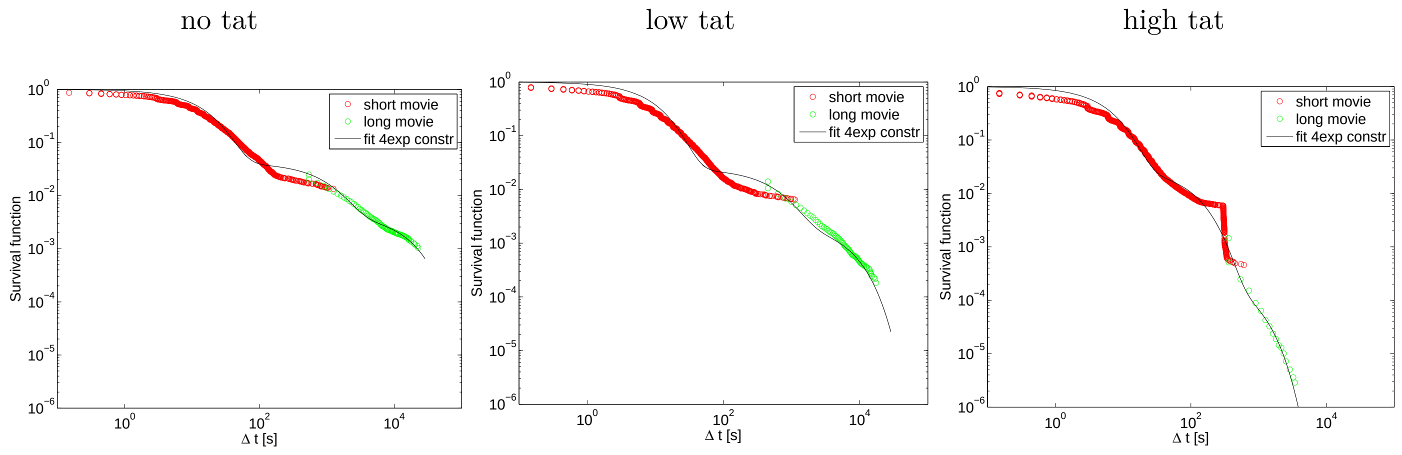

Figure S23: Results of the constrained four-exponential fit of the model M4: most optimal fit for $\alpha = 0.30$.

Type	OBJ	λ_1	λ_2	λ_3	λ_4	A_1	A_2	A_3	A_4
no tat optimal	0.036875	-8430	-0.0636	-0.00103	-6.6e-05	-7.23e-06	0.958	0.0373	0.0043
min		-19700	-0.087	-0.0121	-0.00011	-0.0698	0.869	0.0261	0.00306
max		-1.02	-0.0597	-0.00103	-6.6e-05	-3.06e-06	1.02	0.126	0.00477
low tat optimal	0.078907	-5550	-0.102	-0.0018	-0.000161	-1.79e-05	0.976	0.0221	0.00226
min		-36600	-1.74	-0.0487	-0.000228	-335	0.716	0.0198	0.00212
max		-0.202	-0.0975	-0.00157	-0.000156	-2.97e-06	336	0.453	0.00525
high tat optimal	0.11601	-9330	-0.174	-0.0101	-0.00148	-1.82e-05	0.972	0.0281	0.000288
min		-85200	-0.178	-0.0126	-0.00149	-0.0442	0.968	0.0218	0.000258
max		-4.1	-0.166	-0.00681	-0.00144	-1.91e-06	1.01	0.0323	0.000349

Table S6: Results of the constrained four-exponential fit of the model M4, $\alpha = 0.3$. The objective function shows that the fit is not better than the one of the model M2, for the same α and the fitted parameters are very uncertain.

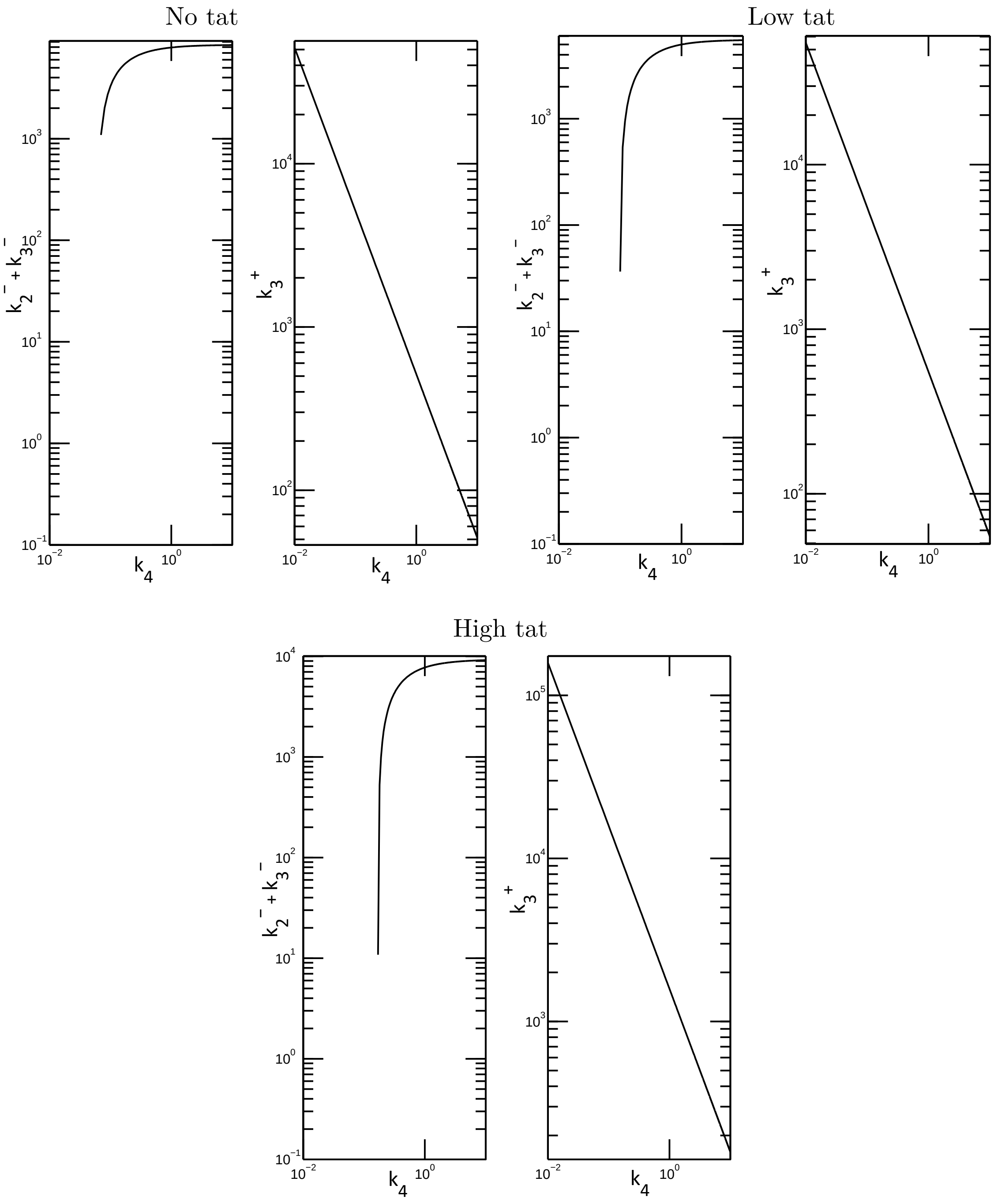

Figure S24: Results of the constrained four-exponential fit of the model M4. Parameter dependence on the undetermined parameter k_4 (pause exit rate) for $\alpha = 0.30$. The parameters k_2^m and $k_3^{\pm}$ have very large values compared to other parameters and correspond to very rapid processes (timescales smaller than $0.1s$). With such parameters the model M4 is equivalent to a three states model with the states ON and PAUSE pooled. For positivity of kinetic parameters one needs $k_4 > 0.1s^{-1}$.

References

[1] A. M. Corrigan, E. Tunnacliffe, D. Cannon, and J. R. Chubb. A continuum model of transcriptional bursting. *Elife*, 5:e13051, 2016.

[2] A. Coulon and D. R. Larson. Fluctuation analysis: dissecting transcriptional kinetics with signal theory. In *Methods in enzymology*, volume 572, pages 159–191. Elsevier, 2016.

[3] M. Dejean, V. L. Pimmet, C. Fernandez, A. Trullo, E. Bertrand, O. Radulescu, and M. Lagha. Quantitative imaging of transcription in living *Drosophila* embryos reveals the impact of core promoter motifs on promoter state dynamics. *preprint*, 2020.

[4] J. Desponds, H. Tran, T. Ferraro, T. Lucas, C. P. Romero, A. Guillou, C. Fradin, M. Coppey, N. Dostatni, and A. M. Walczak. Precision of readout at the hunchback gene: analyzing short transcription time traces in living fly embryos. *PLoS computational biology*, 12(12), 2016.

[5] M. L. Ferguson and D. R. Larson. Measuring transcription dynamics in living cells using fluctuation analysis. In *Imaging gene expression*, pages 47–60. Springer, 2013.

[6] A. Hodgkinson, M. Bellec, A. Devenyi, M. Lagha, and O. Radulescu. Mitotic Memory as Spontaneous Symmetry Breaking in the Cell. *preprint*, 2020.

[7] T. Kato. *Perturbation theory for linear operators*, volume 132. Springer Science & Business Media, 2013.

[8] N. C. Lammers, V. Galstyan, A. Reimer, S. A. Medin, C. H. Wiggins, and H. G. Garcia. Multimodal transcriptional control of pattern formation in embryonic development. *Proceedings of the National Academy of Sciences*, 117(2):836–847, 2020.

[9] J. Rodriguez, G. Ren, C. R. Day, K. Zhao, C. C. Chow, and D. R. Larson. Intrinsic dynamics of a human gene reveal the basis of expression heterogeneity. *Cell*, 176(1-2):213–226, 2019.
