# Supplementary material for: Stochastic pausing at latent HIV-1 promoters generates transcriptional bursting
Source: Nat Commun. 2021 Jul 23;12:4503. doi: 10.1038/s41467-021-24462-5 (PMC8302722; doi:10.1038/s41467-021-24462-5)
Supplement: Supplementary file 2 — Reporting Summary [file 41467_2021_24462_MOESM2_ESM.pdf]

nature research

Corresponding author(s): Edouard Bertrand, Ovidiu Radulescu, Eugenia Basyuk

Last updated by author(s): Jun 10, 2021

Reporting Summary

Nature Research wishes to improve the reproducibility of the work that we publish. This form provides structure for consistency and transparency in reporting. For further information on Nature Research policies, see our Editorial Policies and the Editorial Policy Checklist.

Statistics

For all statistical analyses, confirm that the following items are present in the figure legend, table legend, main text, or Methods section.

n/a	Confirmed	
☐	☒	The exact sample size (*n*) for each experimental group/condition, given as a discrete number and unit of measurement
☐	☒	A statement on whether measurements were taken from distinct samples or whether the same sample was measured repeatedly
☒	☐	The statistical test(s) used AND whether they are one- or two-sided<br>*Only common tests should be described solely by name; describe more complex techniques in the Methods section.*
☒	☐	A description of all covariates tested
☒	☐	A description of any assumptions or corrections, such as tests of normality and adjustment for multiple comparisons
☐	☒	A full description of the statistical parameters including central tendency (e.g. means) or other basic estimates (e.g. regression coefficient) AND variation (e.g. standard deviation) or associated estimates of uncertainty (e.g. confidence intervals)
☒	☐	For null hypothesis testing, the test statistic (e.g. *F*, *t*, *r*) with confidence intervals, effect sizes, degrees of freedom and *P* value noted<br>*Give P values as exact values whenever suitable.*
☒	☐	For Bayesian analysis, information on the choice of priors and Markov chain Monte Carlo settings
☒	☐	For hierarchical and complex designs, identification of the appropriate level for tests and full reporting of outcomes
☒	☐	Estimates of effect sizes (e.g. Cohen's *d*, Pearson's *r*), indicating how they were calculated

Our web collection on statistics for biologists contains articles on many of the points above.

Software and code

Policy information about availability of computer code

Data collection	Metamorph 7.10.3.279, Delta Vision OMX V4
Data analysis	R version 3.2.0. ; Matlab version 9.8 (2020a).<br>Matlab Software for image analysis, deconvolution and modeling is deposited on Github (https://github.com/oradules/Deconvolution_short_long ; https://github.com/oradules/Deconvolution_short), and on Zenodo (DOI: 10.5281/zenodo.4811566).

For manuscripts utilizing custom algorithms or software that are central to the research but not yet described in published literature, software must be made available to editors and reviewers. We strongly encourage code deposition in a community repository (e.g. GitHub). See the Nature Research guidelines for submitting code & software for further information.

Data

Policy information about availability of data

All manuscripts must include a data availability statement. This statement should provide the following information, where applicable:

- Accession codes, unique identifiers, or web links for publicly available datasets
- A list of figures that have associated raw data
- A description of any restrictions on data availability

Data availability statement provided in the manuscript: "The raw movies files are available under restricted access because of the file size, and access can be obtained upon request to EuB or EdB. The processed movie data and the other data generated in this study are provided in the Supplementary Information/Source Data file. This includes data from Figures 1D, 1E, 1G, 1H, 1J, 1K, 2H, 6F, 6G, 7A, 7B, 8B, S1A, S1C, S2C and S3."
A code availability statement is provided separately.

Field-specific reporting

Please select the one below that is the best fit for your research. If you are not sure, read the appropriate sections before making your selection.

- [x] Life sciences
- [ ] Behavioural & social sciences
- [ ] Ecological, evolutionary & environmental sciences

For a reference copy of the document with all sections, see nature.com/documents/nr-reporting-summary-flat.pdf

Life sciences study design

All studies must disclose on these points even when the disclosure is negative.

Sample size	no sample sized calculation was performed. Sample size was estimated from prior studies (Tantale et al. Nature Communications volume 7, Article number: 12248 (2016)), and from computer simulations. Indeed, the feasability of model and parameter reconstruction was tested using artificial data, and experimental sample size was augmented by pooling several movies until we reached values that ensure accurate reconstruction in artificial data. The accuracy obtained with the experimental sample size is illustrated in Figure 5 of the manuscript.
Data exclusions	no
Replication	All experiments were repeated as fully independent experiments at least twice. Live cell image data were acquired independetly on multiple days and pooled together.
Randomization	Randomization is not needed in our study. Genetic variability could be considered as a baseline covariate but this is minimal. On the other hand randomization would reduce the sample size and thus the accuracy of model parameter reconstruction.
Blinding	Blinding was not performed because a single experimentator was performing each experiment. It is also useless because the data gathered from the different cell lines (High Tat, No Tat, Low Tat) are very different from each other and their origin could be recognized even if blinded.

Reporting for specific materials, systems and methods

We require information from authors about some types of materials, experimental systems and methods used in many studies. Here, indicate whether each material, system or method listed is relevant to your study. If you are not sure if a list item applies to your research, read the appropriate section before selecting a response.

Materials & experimental systems

n/a	Involved in the study	
[ ]	[x]	Antibodies
[ ]	[x]	Eukaryotic cell lines
[x]	[ ]	Palaeontology and archaeology
[x]	[ ]	Animals and other organisms
[x]	[ ]	Human research participants
[x]	[ ]	Clinical data
[x]	[ ]	Dual use research of concern

Methods

n/a	Involved in the study	
[x]	[ ]	ChIP-seq
[x]	[ ]	Flow cytometry
[x]	[ ]	MRI-based neuroimaging

Antibodies

Antibodies used	anti FLAG antibodies (Sigma F7425) and secondary anti-rabbit antibody (Jackson ImmunoResearch 711-175-152).
Validation	Validation of anti-FLAG antibodies was done in the lab and included a positive control (a cell line expressing a FLAG-tagged protein) and a negative control (HeLa cells not expressing any FLAG-tagged protein). The antibody is validated by the manufacturer from IP, WB, IF and ICC. The manufacturer validation and specification can be found at https://www.sigmaaldrich.com/specification-sheets/447/086/F7425-BULK________SIGMA____.pdf and https://www.sigmaaldrich.com/FR/en/product/sigma/f7425?context=product.

Eukaryotic cell lines

Policy information about cell lines

Cell line source(s)	Hela H9 cells were a gift of S. Emiliani, Institut Cochin Paris.<br>HEK293 cells were obtained from M. Sitbon, Institut de Génétique Moléculaire de Montpellier.
Authentication	none of the cell lines were authenticated.
Mycoplasma contamination	cell lines were regularly tested for mycoplasma (once/week to once/month)

Commonly misidentified lines<br>(See ICLAC register)	no commonly misidentified cell line that we know of.
