# Supplementary figures and images for: Stochastic pausing at latent HIV-1 promoters generates transcriptional bursting
Source: Nat Commun. 2021 Jul 23;12:4503. doi: 10.1038/s41467-021-24462-5 (PMC8302722; doi:10.1038/s41467-021-24462-5)

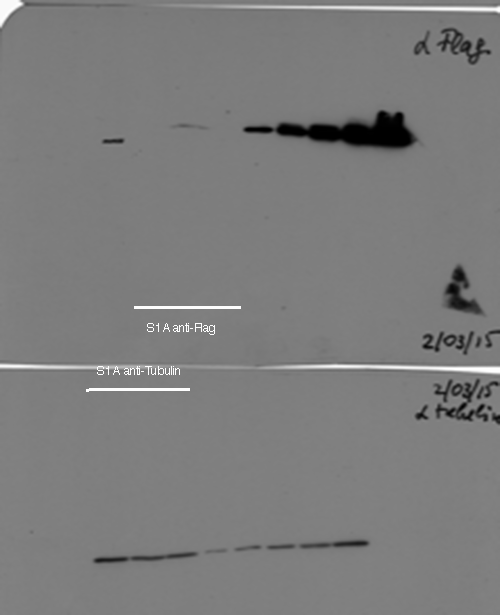

Supplement: Supplementary file 3 — Source Data [file 41467_2021_24462_MOESM3_ESM.zip › _Source_data_zip/Figure_S1A_WesternBlot.tif]
